# Mechanism Investigation of Wuwei Shexiang Pills on Gouty Arthritis via Network Pharmacology, Molecule Docking, and Pharmacological Verification

**DOI:** 10.1155/2022/2377692

**Published:** 2022-10-07

**Authors:** Jirui Lang, Li Li, Shilong Chen, Yunyun Quan, Jing Yi, Jin Zeng, Yong Li, Junning Zhao, Zhujun Yin

**Affiliations:** ^1^West China School of Pharmacy, Sichuan University, Chengdu, China; ^2^Translational Chinese Medicine Key Laboratory of Sichuan Province, Sichuan Academy of Chinese Medicine Sciences, Chengdu, China; ^3^Translational Chinese Medicine Key Laboratory of Sichuan Province, Sichuan Institute for Translational Chinese Medicine, Chengdu, China; ^4^Sichuan Fengchun Pharmaceutical Co, Ltd, Deyang, China

## Abstract

**Background:**

Gout is a common crystal-related arthritis caused by the deposition of monosodium urates (MSU). Tibetan medicine Wuwei Shexiang Pills (WSP) has been demonstrated to exhibit anti-inflammatory, antihyperuricemia, and antigout activities. However, the underlying mechanism is unknown.

**Objectives:**

To explore the mechanisms of Wuwei Shexiang Pills on gouty arthritis via network pharmacology, molecule docking, and pharmacological verification.

**Methods:**

The ingredients and targets of WSP were obtained by searching and screening in BATMAN-TCM and SwissADME. The targets involving the gout were acquired from public databases. The shared targets were put onto STRING to construct a PPI network. Furthermore, Metascape was applied for the GO and KEGG enrichment analysis to predict the biological processes and signaling pathways. And molecular docking was performed to validate the binding association between the key ingredients and the relative proteins of TNF signaling. Based on the serum pharmacology, the predicted antigout mechanism of WSP was validated in MSU-induced THP-1 macrophages. The levels of inflammatory cytokines and mRNA were measured by ELISA and qRT-PCR, respectively, and MAPK, NF-*κ*B, and NLRP3 signaling-associated proteins were determined by western blot and immunofluorescence staining.

**Results:**

48 bioactive ingredients and 165 common targets were found in WSP. The data showed that 5-Cis-Cyclopentadecen-1-One, 5-Cis-Cyclotetradecen-1-One, (−)-isoshyobunone, etc. were potential active ingredients. TNF signaling, HIF-1 signaling, and Jak-STAT signaling were predicted to be the potential pathways against gout. The molecule docking analysis found that most ingredients had a high affinity for p65, NLRP3, IL-1*β*, TNF-*α*, and p38. The data from *in vitro* experiment showed that WSP suppressed the production and gene expression of inflammatory cytokines. Furthermore, WSP could inhibit the activation of MAPK, NF-*κ*B, and NLRP3 signaling pathways.

**Conclusion:**

Our finding suggested that the antigout effect of WSP could be achieved by inhibiting MAPK, NF-*κ*B, and NLRP3 signaling pathways. WSP might be a candidate drug for gouty treatment.

## 1. Introduction

Gout is a common and systematic disease, particularly prevalent in joints caused by monosodium urate (MSU) crystals deposition, whose incidence is continuously increasing worldwide [1, 2]. Nowadays, the global prevalence of gout ranges from 0.68% to 3.90% in adults [1] while the incidence rate is about 1% in China, showing a younger trend [3]. Long-term high serum uric acid (hyperuricemia) without adequate urate-lowering treatment is a considerable risk factor for gout flares [1, 4]. Clinically, the acute episode of gouty arthritis (GA) is characterized by intolerably painful arthritis, especially the first metatarsophalangeal joint swelling and tophus [5]. Although most hyperuricemia patients have no gouty clinical manifestations and even will not develop gout for their whole life [2], symptomatic or asymptomatic hyperuricemia suffers a higher risk of diabetes mellitus [6], obesity [7], metabolic syndrome [8], hypertension [9], fatty liver [10], urinary stones [11], and kidney injury [12]. According to the 2020 guideline for the management of gout issued by the American College of Rheumatology (ACR), nonsteroidal anti-inflammatory drugs (NSAIDs), oral corticosteroids, and colchicine were recommended as the first-line antigout agents [13]. However, multiple severe toxic events have been reported in the application of these agents [1, 14]. Therefore, it is urgent and necessary to develop safer and more curative novel drugs for gout treatment.

Traditional Chinese medicine (TCM) has been applied as a valid complementary therapy for the treatment of gout and hyperuricemia in the clinic, which has gained extensive attention in the world owing to its efficiency and safety [15, 16]. Wuwei Shexiang Pills (*Qiong-e* in Tibetan, WSP), composed of *Acorus calamus* L. (Zang Chang Pu in Chinese, ZCP), *Terminalia chebula* Retz. (He Zi in Chinese, HZ), *Aucklandia lappa* Decne. (Mu Xiang in Chinese, MX), artificial Moschus (She Xiang in Chinese, SX), and *Aconitum pendulum* Busch (Tie Bang Chui in Chinese, TBC) (the details of herbal ingredients are shown in Table 1), have been used for the treatment of various arthritis among Tibetans in China for centuries, which was documented originally in the *Sibuyidian* (8^th^ century AD, a well-known Tibetan medicine classic). It has been demonstrated that WSP exhibited anti-inflammatory and analgesic actions, which was commonly used in patients with tonsillitis, pharyngitis, rheumatic arthritis, and gouty arthritis [17, 18]. Our previous studies confirmed that WSP could significantly inhibit paw edema and the release of inflammatory cytokines in MSU-induced acute gouty arthritis in rats (Supplementary file 1). However, the material basis and the pharmacological mechanisms of WSP have not been elucidated.

Currently, the application of network pharmacology, molecule docking, serum pharmacology, serum pharmacochemistry, pharmacokinetic analysis, and molecular biochromatography have shown advantages and good prospects in the exploration of the material basis and pharmacological mechanism of TCM [19, 20]. Network pharmacology, an emerging interdisciplinarity, has been used in the discovery of active compounds and the interpretation of the overall mechanisms in the TCM and its formulas, by collecting and analyzing data from bioinformational databases associated with herbs, disease targets, and pathways [21]. To verify the prediction of network pharmacology, serum pharmacology is commonly combined to explore the specific mechanisms of TCM by applying drug-containing serum *in vitro*. Pieces of literature combined network pharmacology with serum pharmacology have been successfully reported [22–24].

**Figure 1:**
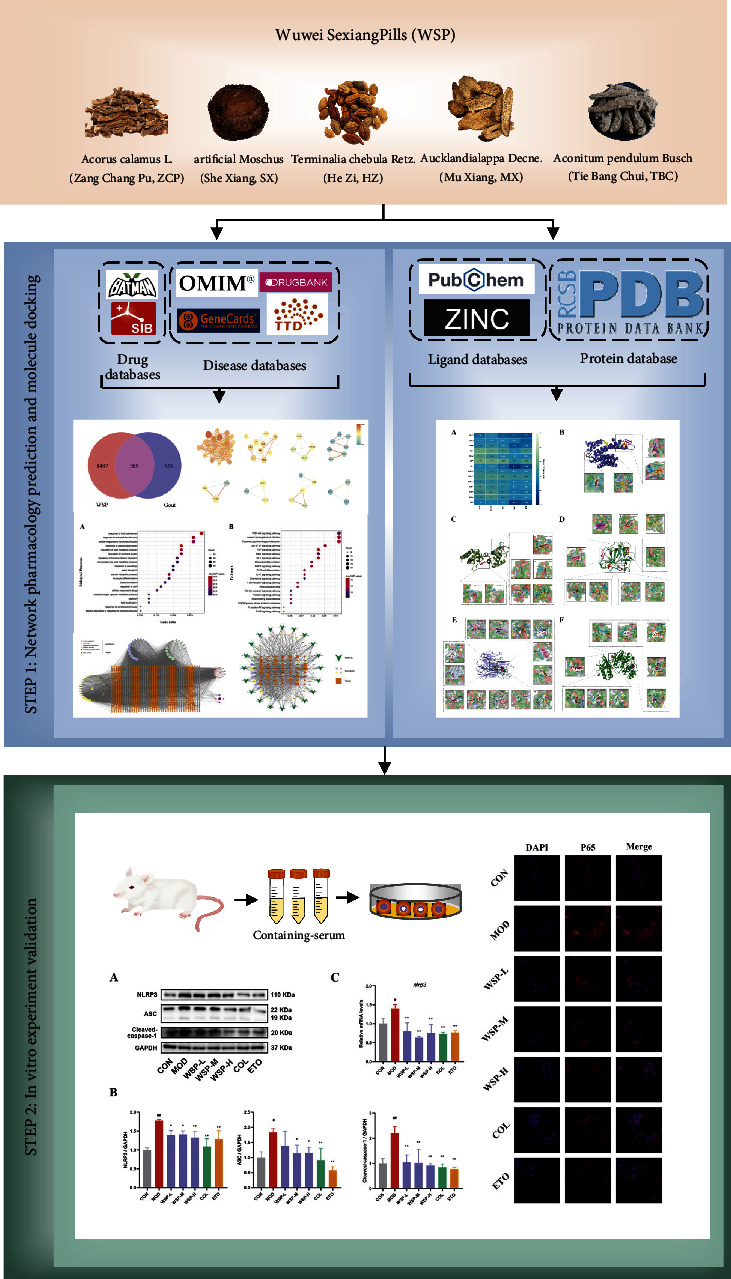
Workflow graph of this study.

To figure out the pharmacological mechanisms of WSP against gout, network pharmacology was adopted to establish a network relationship between WSP and gout via obtaining the active ingredients of herbs, potential targets, and pathways of WSP. Subsequently, molecular docking technology was performed to forecast the binding form and the affinity between the key ingredients of WSP and the vital proteins such as p65, NLRP3, IL-1*β*, TNF-*α*, and p38 of TNF signaling, which were the crucial targets closely related to gout disease. Finally, the crucial signaling pathways predicted by network pharmacology were confirmed in the MSU-induced THP-1 macrophages, thus revealing the underlying mechanisms of WSP against gout. The main workflow is shown in Figure 1.

## 2. Materials and Methods

### 2.1. Step 1: Prediction of WSP in the Treatment of Gout Combined Network Pharmacology and Molecule Docking Analysis

#### 2.1.1. Acquirement of Ingredients and Targets of WSP

The bioactive ingredients and potential targets of five herbs in WSP were obtained in the online Bioinformatics Analysis Tool for Molecular mechANism of TCM (BATMAN-TCM, http://bionet.ncpsb.org.cn/batman-tcm/) database [25]. The screening conditions were set at a score cutoff = 20, *p* < 0.05 [26, 27]. BATMAN-TCM is a bioinformatics analysis tool for the molecular mechanism of TCM with abundant herb-ingredient-target-disease association information and integration analyses of targets, which can help researchers comprehensively and systematically understand the ingredients and targets of TCM preparations. Then, the results were input onto the SwissADME (http://www.swissadme.ch/) to obtain the components with potential pharmaceutical activity. SwissADME is an online data simulation platform that allows researchers to compute physicochemical descriptors as well as to predict ADME parameters, pharmacokinetic properties, druglike nature, and medicinal chemistry friendliness of one or multiple small molecules to support drug discovery [28]. The filter criteria were set at “Pharmacokinetics: gastrointestinal absorption = high” and “quantity of ‘yes' of druglikeness more than 2” [29].

All the names of protein targets were standardized in the Universal Protein Resource (UniProt, https://www.uniprot.org/) database [30]. UniProt is a collection of information on proteins with specification and accuracy [31]. The protein names were converted to standard gene symbols filtered by “Swiss-Prot reviewed” and “*Homo sapiens*”.

#### 2.1.2. Gout Targets Collection

The human genes associated with gout were searched and screened from Online Mendelian Inheritance in Man® (OMIM®, https://omim.org/) database [32], Drugbank (https://go.drugbank.com/) database [33], GeneCards® (https://www.genecards.org/) knowledgebase [34], and Therapeutic Target Database (TTD, http://db.idrblab.net/ttd/) [35] by keywords “gout”, “gouty arthritis”, and “gout flares.” The targets of gout were obtained by integrating the above results and deleting duplicate values. And the target names were converted to standard gene symbols in UniProt as well.

#### 2.1.3. Protein-Protein Interactions (PPI) Network Construction and Analysis

According to the intersection of WSP-targets and gout targets in Excel 2019, the common targets were obtained and the Venn diagram was drawn. The common targets were uploaded to the Search Tool for the Retrieval of Interacting Genes (STRING, https://string-db.org/v.11.5) [36] with parameters filtered by “*Homo sapiens*” (interaction score >0.4), which constructed the PPI network and provided information and analyses. The PPI network was input to Cytoscape 3.7.2 software to explore the modules inside by using the MCODE plug-in.

#### 2.1.4. GO and KEGG Enrichment Analysis

Metascape (https://metascape.org/gp/index.html#/main/step1) is a reliable, productive, intuitive, and continuously updated tool that contains accurate and complete gene annotation and gene list enrichment analysis [37]. The common targets between WSP and gout were submitted to the Metascape and the conditions were set at *p* value < 0.01, minimum count of 3, and enrichment factor >1.5 filtered by “*H. sapiens*”. To investigate the specific biological processes and signaling pathways of WSP against gout, the gene ontology (GO) and Kyoto Encyclopedia of Genes and Genomes (KEGG) enrichment analysis were applied.

#### 2.1.5. Ingredient-Target-Pathway Network Construction

To unveil the pharmacological mechanisms of WSP against gout, the common targets, ingredients of WSP, and pathways were put into Cytoscape 3.7.2 to construct an ingredient-target-pathway network. And the topological parameters of this complicated network were measured by running the Network Analyzer plug-in of Cytoscape 3.7.2 [31, 38].

#### 2.1.6. Molecule Docking

The 3D structure files of ingredients (high degree in the prediction results) were obtained from ZINC (https://zinc.docking.org/) database [39] or PubChem® (https://pubchem.ncbi.nlm.nih.gov/). All the files of ligands were saved in ^*∗*^mol2 format. The macromolecule crystal structure files of the proteins were obtained from RCSB PDB (https://www.rcsb.org/) database [40] and saved in ^*∗*^pdb format. The protein file was imported to AutoDock 1.5.6 software (The Scripps Research Institute, CA, USA) for pretreatment including removal of water, adding hydrogens, and saving in ^*∗*^pdbqt format. The ligand was added hydrogens, detected roots and torsions, and saved in ^*∗*^pdbqt format. Then, docking parameters were set as follows: “rigid filename,” “search parameters: genetic algorithm,” and “docking parameters: default”. The binding energy <0 indicted the ligand can spontaneously bind the macromolecule. The results of docking were put into PyMOL 2.3.0 software (© Schrödinger) for visualization.

### 2.2. Step 2: Experimental Validation Using Serum Pharmacology

#### 2.2.1. Regents and Drugs

All the materials were obtained from the suppliers as follows: THP-1 cell line (Guangzhou Cellcook Biotech Co., Ltd, Guangzhou, China); RPMI-1640 culture medium and phosphate-buffered saline (PBS) (Gibco, NY, USA); fetal bovine serum (FBS, HyClone, UT, USA); cell counting kit-8 (CCK-8) and ECL kit (Biosharp, Hefei, China); the enzyme-linked immunosorbent assay (ELISA) kits for human IL-1*β* and TNF-*α* (ExCell, Taicang, China); RIPA protein lysate and BCA protein assay kit (Boster, Wuhan, China); protease inhibitor mixture and protein phosphatase inhibitor (Solarbio, Beijing, China); SDS-PAGE sample loading buffer, skim milk powder, and NF-*κ*B nuclear translocation assay kit (Beyotime. Shanghai, China); bovine serum albumin (BSA) (Sigma-Aldrich, MO, USA), MSU crystal (InvivoGen, Hong Kong SAR, China); gel premade glues (WSHT, Shanghai, China); PAGE transfer buffer powder and TBST (Servicebio, Wuhan, China); PVDF transfer membrane (0.45 *μ*m and 0.22 *μ*m, Millipore, Schwalbach, Germany); anti-phospho-JNK1/2/3 (Thr183+Tyr185) antibody (Abs130943), anti-phospho-p38 MAPK (Thr180/Tyr182) antibody (Abs131122), and anti-phospho-ERK1/2 (Thr202/Tyr204) antibody (Abs130614, Absin, Shanghai, China); anti-JNK antibody (9252), anti-p38 MAPK antibody (8690), anti-Erk1/2 antibody (4695), anti-NLRP3 antibody (15101), anti-phospho-I*κ*B*α* (Ser32) antibody (2859), anti-I*κ*B*α* antibody (4812), anti-phospho–NF–*κ*B p65 (Ser536) antibody (3033), anti-NF-*κ*B p65 antibody (8242), anti-ASC antibody (13833), anti-GAPDH antibody (8884), anti-Phospho-IKK*α*/*β* (Ser176/180) antibody (2697), anti-IKK*α* antibody (61294), and anti-IKK*β* antibody (8943) (Cell Signaling Technology Inc. (CST), MA, USA); prestained protein ladder (Thermo Scientific, MA, USA); total RNA isolation kit (Foregene, Chengdu, China); 5 × All-In-One MasterMix, EvaGreen Express 2 × qPCR MasterMix (Abm, Vancouver, Canada); WSP (Z51020967) (Jiuzhaigou Natural Pharmaceutical Group Co., Ltd., Aba, China); Colchicine tablets (H53020166) (Yunnan Pharmaceutical Group Co., Ltd., Kunming, China); and Etoricoxib tablets (J20180059) (Merck and Co., Inc., NJ, USA).

#### 2.2.2. Animals and Treatments

A total of 40 male-specific pathogen-free Sprague-Dawley rats (6∼8 weeks, 280∼300 g) were purchased from the center of experimental animals of Sichuan Academy of Chinese Traditional Medicine (license number: SCXK (Chuan) 2018–19). After 1 week of acclimatization, the rats were randomly divided into 4 groups including the WSP group, colchicine group, etoricoxib group, and blank serum group which were orally administrated with responding drugs at the doses of 40 mg/kg, 0.32 mg/kg, and 32 mg/kg or water vehicle once a day for 8 days, respectively. All the animals were housed under the standard condition with regulated temperature and humidity with an alternating 12-hour light/12-hour dark cycle, allowing them to take food and water freely. After the experiment, the rats were sacrificed by asphyxiation with CO_2_ (KW-AL experimental animal euthanasia device, Nanjing Calvin Biotech. Co., Ltd.), which was delivered into the cages at less than 5 psi per second. The death of the rats was confirmed by a lack of respiration and consciousness. All the experimental procedures were approved by the Experimental Animal Ethics Committee of the Sichuan Academy of Chinese Traditional Medicine (Grant number: SYLL (2021)-031).

#### 2.2.3. Preparation of the Blank Serum and Serum-Containing WSP, Colchicine, and Etoricoxib

On day 8, animals were anesthetized with an intraperitoneal injection of pentobarbital sodium at a dose of 40 mg/kg after 1 hour of the last treatment. Then, the blood was collected through the abdominal aorta for the preparation of serum. After 30 min at room temperature, the blood samples were centrifuged at 4,000 r/min at 4°C for 15 min to obtain the serum. The containing serum was inactivated at 56°C for 30 min, filtered through a 0.22 *μ*m microporous membrane in the sterile environment, and stored at -20°C for cellular experiment [41].

#### 2.2.4. Cell Counting Kit-8 (CCK-8) Assay

The THP-1 cells were cultured in the RPM1-1640 culture medium containing 10% FBS, 100 mg/ml streptomycin, and 100 U/ml penicillin at 37°C and a 5% CO_2_ incubator (Thermo Scientific, MA, USA). When the cells were at the logarithmic growth phase, 100 ng/mL phorbol-12-myristate-13-acetate (PMA) was added to the medium, inducing THP-1 cells differentiated into macrophages. The CCK-8 assay was used to detect the cell viability of THP-1 macrophages. THP-1 cells were plated in 96-well plates with a density of 5 × 10^4^/well. Treated with PMA and rested for 1 d, the different concentrations of the containing serum (5%, 10%, 20%, 30%, 40%, 60%) were added to the medium for 1 h, and MSU was added to the medium for 24 h. Then, the CCK-8 working solution was added to the wells (10 *μ*L/well) for 1 h after discarding the medium. The absorbance was measured at 450 nm with a microplate reader (TECAN Sunrise™, Grödig, Austria). Cell viability ratio (%) was determined as follows: cell viability ratio (%) = [OD (treat) − OD (blank)]/[OD (control) − OD (blank)] × 100%.

#### 2.2.5. Experimental Groups and Sample Collection

**Figure 2:**
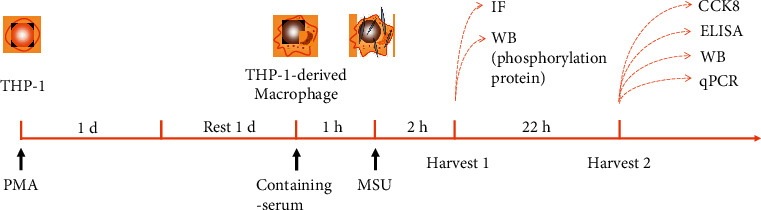
*In vitro* experiment protocol.

PMA-induced THP-1 macrophages were classified into 8 groups, including the control group (CON, complete medium), model group (MOD, complete medium + 100 *μ*g/mL MSU), low-dose group (WSP-L, complete medium + 20% containing WSP serum + 100 *μ*g/mL MSU), mid-dose group (WSP-M, complete medium + 30% containing WSP serum + 100 *μ*g/mL MSU), high-dose group (WSP-H, complete medium + 40% containing WSP serum + 100 *μ*g/mL MSU), colchicine group (COL, complete medium + 40% containing-colchicine serum + 100 *μ*g/mL MSU), etoricoxib group (ETO, complete medium + 40% containing-etoricoxib serum + 100 *μ*g/mL MSU), and blank serum group (complete medium + 40% blank serum + 100 *μ*g/mL MSU). The subsequent experiment procedures were described in Figure 2. Briefly, the cell samples were collected for the detection of the phosphorylation of the target proteins and immunofluorescence assay after MSU crystal stimulation for 2 h. Whereafter, the samples were harvested for the detection of the cell viability and release of inflammatory cytokines with 24 h MSU insult, as well as the cells for the detection of gene and protein expression.

#### 2.2.6. Enzyme-Linked Immunosorbent Assay (ELISA)

Treated by the different concentrations of test serum for 1 h, namely, 20%, 30%, and 40% containing WSP of serum, 40% containing-colchicine serum, 40% containing-etoricoxib serum, and 40% blank serum, THP-1-derived macrophages were stimulated by 100 *μ*g/mL MSU for 24 h, except the control group. Then, the supernatant was determined by ELISA for IL-1*β*, TNF-*α*, IL-6, and IL-18 contents according to the manufacturer's instructions.

#### 2.2.7. Western Blot

Provoked by MSU crystal, the total protein of cells was collected at 2 h or 24 h, respectively. For removal and cleaning of the medium, the RIPA protein lysate containing 1% protease inhibitor mixture and 1% protein phosphatase inhibitor was added to the plates. The lysates were centrifuged at 12,000 × *g* for 15 min at 4°C after ultrasonic wave breaking. Then, the supernatant was collected and the concentration was measured by BCA assay. Then, the supernatant was added to a 5×loading buffer and heated for denaturation at 95°C for 5 min. The proteins were separated by 10% or 15% sodium dodecyl sulfate-polyacrylamide gel electrophoresis (SDS-PAGE) and transferred onto the 0.22 *μ*m or 0.45 *μ*m PVDF membranes, based on the molecular weight of the target proteins. Blocked with the 5% nonfat milk or BSA in TBST, the membranes were incubated with primary antibody (diluted 1 : 1000) at 4°C overnight. Then, the primary antibody was washed and incubated with horseradish peroxidase- (HRP-) linked secondary antibody (diluted 1 : 10000) for 1 hour at room temperature. Finally, the protein bands were probed with the ECL kit and exposed to the imaging system (Tanon, Shanghai, China).

#### 2.2.8. Quantitative Real-Time Polymerase Chain Reaction (qRT-PCR) Assays

The mRNA expression levels of related targets were measured by qRT-PCR assays. The THP-1 cells with PMA were plated on the 6-well plates overnight. After the replacement of fresh medium and rest for 1 d, the containing serum and MSU crystal were incubated with cells for 24 h. Total RNA of THP-1 macrophages was isolated by TRIzol regent, which was purified and reverse-transcribed according to the manufacturer's instructions. The qRT-PCR reaction was performed using an SYBR Green PCR kit. The primer sequences are shown in Table 2. The qRT-PCR conditions were as follows: predenaturation for 10 min at 95°C, 40 cycles of a 10 s denaturation at 95°C, and a 30 s amplification at 60°C. The relative gene expression was detected by the Cycler threshold (2^−Δct^) method and was normalized to the housekeeping gene *β*-Actin.

#### 2.2.9. Immunocytochemical Analysis

The translocation of NF-*κ*B was observed by fluorescence microscope (Carl Zeiss, Jena, Germany) using the immunocytochemical kit according to the manufacturer's instructions. Briefly, after incubation with WSP and MSU crystal for 24 h, the cells were fixed and washed. Then, samples were incubated with the primary antibody overnight at 4°C and added to the secondary antibody Cy3 in turns. Whereafter, the nuclear was stained with DAPI for 5 min. Finally, the cells were observed and plotted in the darkroom.

#### 2.2.10. Statistics Analysis

All data was expressed as the mean ± standard deviation. The image data were analyzed by the ImageJ 1.8.0 software (NIH and LOCI, USA) and ZEN (Carl Zeiss, Jena, Germany). One-way analysis of variance (ANOVA) was used for multifactorial comparisons using GraphPad Prism 9 (GraphPad Software, Inc., CA, USA). The *p* value < 0.05 was considered statistically significant.

## 3. Results and Discussion

### 3.1. Herb-Ingredient-Target Network of WSP

Due to the characteristic of multiple compounds and multiple targets of TCM, we constructed a herb-ingredient-target network of WSP to clarify the relationships between various ingredients of herbs and potential targets (Supplementary file 2). According to the results of BATMAN-TCM and SwiessADME screening, a total of 88 ingredients and 1652 targets were included. The amount of ingredients in the herbs was as follows: ZCP (degree = 23), HZ (degree = 10), MX (degree = 33), SX (degree = 18), and TBC (degree = 4) (details were shown in Supplementary file 3).

### 3.2. Gout Targets

By searching the targets associated with “gout” “gout arthritis” and “gout flares” in OMIM®, Drugbank, GeneCards®, and TTD disease databases, we obtained 718 targets after merging results and deleting duplicate terms.

### 3.3. PPI Network of Common Targets

**Figure 3:**
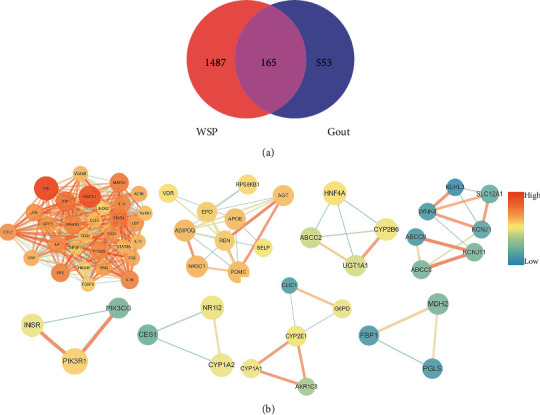
(a) Venn diagram. The value demonstrated the number of shared and unique targets of WSP and gout. (b) A total of 8 modules have been found in the PPI network. The color depths of nodes are positively correlated with their degrees. The size and color depths of edges are positively correlated with the interaction degree of the two nodes.

A total of 165 common targets shared by both WSP and gout were obtained via Venn analysis (Figure 3(a)), which were listed in the Supplementary file 4 Table S4. A PPI network was constructed with these targets (disconnected nodes were hidden) in the STRING (Supplementary file 5).

In the PPI network, the highly connected dense regions called modules usually represent important and relevant biological activities [42]. Thus, it is of great significance to analyze the PPI network by exploring these subnetworks. These functional modules were identified by the MCODE plug-in of Cytoscape 3.7.2 and a total of 8 modules were acquired (Figure 3(b)). Through the analysis of the 8 modules, the biological processes with the top 5 scores were retained (Table 3).

### 3.4. GO and KEGG Enrichment Analysis

**Figure 4:**
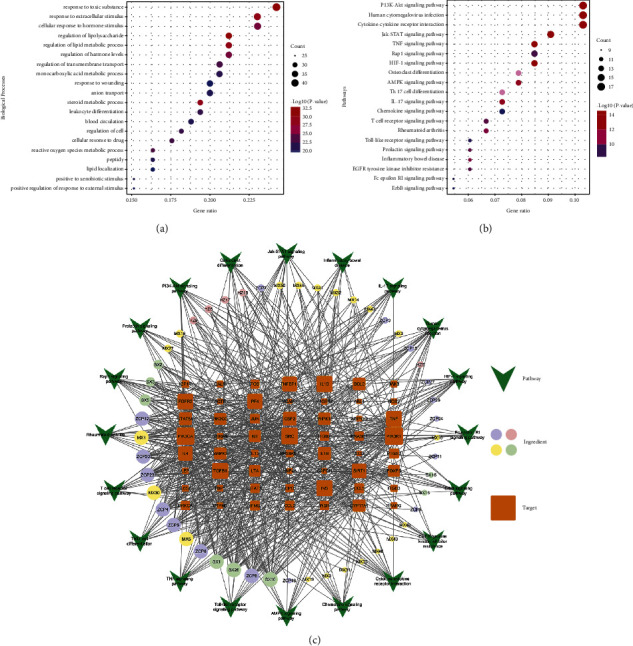
(a) GO biological processes of WSP against gout. (b) KEGG enrichment analysis results. The color scale represented the –Log10 (*p* value), and the dot size indicated the gene count in each term. (c) The ingredient-target-pathway network. The different colors indicated the different ingredients of herbs. The size of nodes was positively correlated with their degrees. The bigger the nodes are, the more important they were.

The results of GO and KEGG enrichment analysis based on the Metascape database showed the biological function and pathways of WSP against gout. The WSP was mainly involved in 2164 biological processes including “response to lipopolysaccharide,” “response to toxic substance,” “response to extracellular stimulus,” “regulation of lipid metabolic process,” “steroid metabolic process,” “cellular response to hormone stimulus,” and “regulation of hormone levels”, and top 20 biological processes were retained (Figure 4(a)). A total of 328 pathways were mapped by the KEGG enrichment. Unrelated results such as pathways in cancer, Chagas disease, and nonalcoholic fatty liver disease were excluded and the top 20 pathways were retained (Figure 4(b)). The targets-pathways results are shown in Table 4, mainly containing TNF signaling pathway, HIF-1 signaling pathway, Jak-STAT signaling pathway, and others.

### 3.5. Ingredient-Target-Pathway Network

Through the above work, it has been revealed that the correlation between the ingredients of WSP and gout targets, biological processes, cellular pathways, and core targets interfered by WSP. To uncover the association between ingredients, targets, and pathways, the related information was put into Cytoscape 3.7.2 to construct the tripartite network (Figure 4(c)).

According to the analysis results, SX10 (5-Cis-Cyclopentadecen-1-One), SX26 (5-Cis-Cyclotetradecen-1-One), and ZCP8 ((-)-isoshyobunone) were predicted as the main biological ingredients, and PIK3R1 and PIK3CA were predicted as the main targets. And the network characteristic parameters of other important ingredients and targets of WSP against gout are listed in Tables 5 and 6.

### 3.6. Molecule Docking Analysis

**Figure 5:**
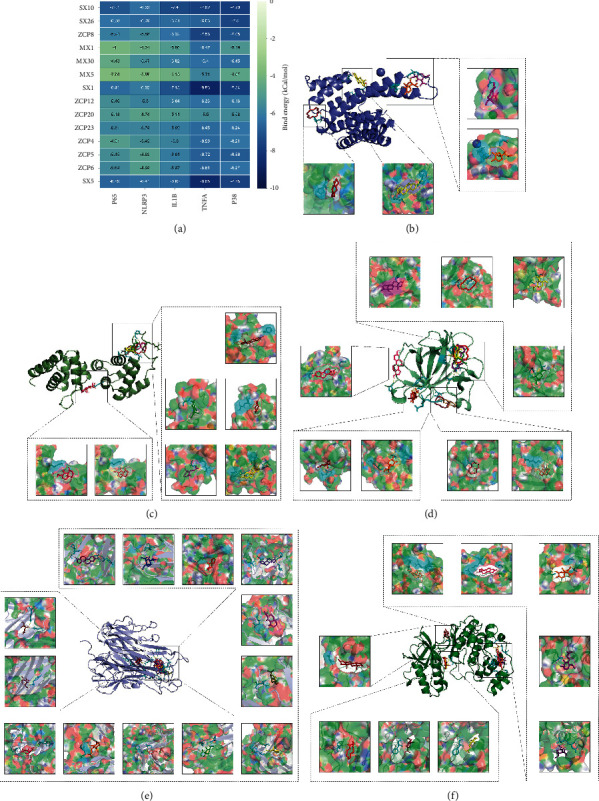
Molecule docking results of the main active components and the key TNF-associated targets. (a) The number in the grid represents the binding energy between ligands and macromolecules. The darker the color is, the stronger the affinity is. (b) 4 ingredients (SX10 (5-Cis-Cyclopentadecen-1-One), ZCP8 ((-)-isoshyobunone), ZCP6 (Acoronene), and SX5 (Estradiol)) and NF-*κ*B P65. (c) 7 ingredients (SX26 (5-Cis-Cyclotetradecen-1-One, ZCP8 ((-)-isoshyobunone), MX30 (Beta-Ionone), SX1 (Androstenedione), ZCP12 (Acolamone), ZCP23 (Isoacolamone), and SX5 (Estradiol)) and NLRP3. (d) 9 ingredients (SX10 (5-Cis-Cyclopentadecen-1-One, SX26 (5-Cis-Cyclotetradecen-1-One), ZCP8 ((-)-isoshyobunone), MX30 (Beta-Ionone), SX1 (Androstenedione), ZCP12 (Acolamone), ZCP4 (Shyobunone), ZCP6 (Acoronene), and SX5 (Estradiol)) and IL-1*β*. (e) 13 ingredients (SX10 (5-Cis-Cyclopentadecen-1-One), ZCP8 ((-)-isoshyobunone), MX1 (Artemisia Ketone), MX30 (Beta-Ionone), MX5 ((E)-6,10-Dimethyl-9-methylene-5-undecen-2-one), SX1 (Androstenedione), ZCP12 (Acolamone), ZCP20 (Calacone), ZCP23 (Isoacolamone), ZCP4 (Shyobunone), ZCP5 (Acoragermacrone), ZCP6 (Acoronene), and SX5 (Estradiol)) and TNF-*α*. (f) 9 ingredients (SX10 (5-Cis-Cyclopentadecen-1-One), ZCP8 ((-)-isoshyobunone), MX30 (Beta-Ionone), SX1 (Androstenedione), ZCP20 (Calacone), ZCP23 (Isoacolamone), ZCP4 (Shyobunone), ZCP6, and SX5 (Estradiol)) and MAPK P38.

Molecule docking was performed to further identify the potential effect of the top 14 ingredients of WSP on relative proteins of TNF signaling, including NF-*κ*B P65, NLRP3, IL-1*β*, TNF-*α*, and MAPK P38. The binding free energies are shown in Figure 5(a). It was generally recognized that the lower binding energy implicated the higher possibility of their combination and the better stability of the ligand-macromolecule pair. As shown in Figure 5(b), a total of 4 docking sites of NF-*κ*B P65 (HIS-106, LYS-77, GLY-78, and ARG-82, GLU-20, and ARG-132) were found. SX10 performed a strong affinity (binding energy = −7.01 kCal/mol) for NF-*κ*B P65. And SX26 (5-Cis-Cyclotetradecen-1-One), ZCP8 ((−)-isoshyobunone), MX30 (Beta-Ionone), SX1 (Androstenedione), ZCP12 (Acolamone), ZCP23 (Isoacolamone), and SX5 (Estradiol) showed high affinity for NLRP3 (Figure 5(c)). Subsequently, a total of 9 components might bind to IL-1*β*. Notably, the binding energy between SX1 and IL-1*β* was the lowest and they formed 3 H-bounds (Figure 5(d)). Interestingly, almost all the ingredients showed a very high affinity for TNF-*α*. In the docking site of “SER-99, PRO-100, GLN-102, and TYR-115”, SX1 and SX5 formed 5 H-bounds and performed a strong corelationship for TNF-*α*, respectively (Figure 5(e)). Besides, a total of 9 ingredients (SX10 (5-Cis-Cyclopentadecen-1-One), SX26 (5-Cis-Cyclotetradecen-1-One), ZCP8 ((-)-isoshyobunone), MX30 (Beta-Ionone), SX1 (Androstenedione), ZCP12 (Acolamone), ZCP4 (Shyobunone), ZCP6 (Acoronene), and SX5 (Estradiol)) might connect to MAPK P38 protein (Figure 5(f)). The docking results implied that WSP had a potential regulatory effect on the associated key targets of TNF signaling in GA sufferings.

### 3.7. WSP Attenuated the Production of Inflammatory Cytokines in MSU-Activated THP-1 Macrophages

**Figure 6:**
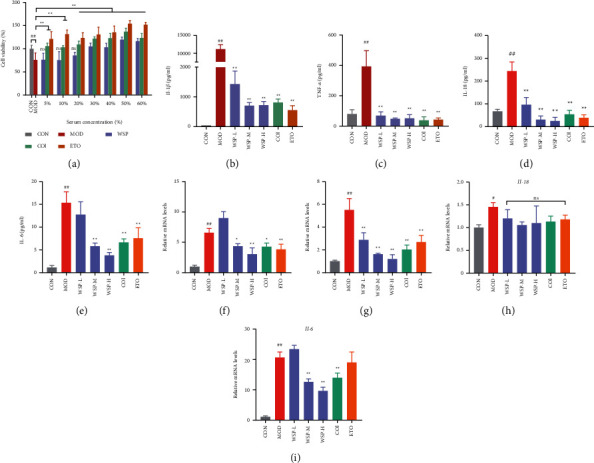
Effect of containing serum on cell viability and WSP attenuated the production of inflammatory cytokines in MSU-activated THP-1 macrophages. (a) The viability of MSU-activated THP-1 macrophages was evaluated by CCK-8 assay. Values are mean ± SD (*n* = 6). ##*p* < 0.01 vs. CON group; ^*∗∗*^*p* < 0.01 vs. MOD group. (b–e) Levels of IL-1*β*, TNF-*α,* IL-18, and IL-6 in the supernatant of MSU-activated THP-1 macrophages measured by ELISA. Values are mean ± SD (n = 4∼5). (f–i) Relative mRNA levels of IL-1*β*, TNF-*α*, IL-18, and IL-6 of MSU-activated THP-1 macrophages measured by qRT-PCR. Values are mean ± SD (n = 3). #*p* < 0.05, ##*p* < 0.01 vs. CON group; *∗p* < 0.05, ^*∗∗*^*p* < 0.01 vs. MOD group.

Firstly, the cell viability of MSU crystal-induced THP-1-derived macrophage treated with serum-containing WSP was evaluated by a CCK-8 kit. As shown in Figure 6(a), the cell viability of the model group decreased significantly after MSU crystal stimulation, compared to the control group. Pretreated with medicated serum for 24 h, the cell viability of WSP groups (concentration of medicated serum ranges from 30% to 60%) increased prominently, while WSP groups (concentration ranges from 5% to 20%) had no obvious effect, compared to the model group. Meanwhile, the elevated effect of colchicine and etoricoxib on cell viability exhibited a concentration-dependent trend. Therefore, the concentration of 20%∼40% serum-containing WSP was applied in further trials. Besides, 40% medicated serum of colchicine and etoricoxib was selected for the positive control. Moreover, we tested the effect of 20%, 30%, and 40% blank serum on THP-1 macrophages. The results showed that the blank serum could not reverse the MSU-induced decrease in cell viability (Supplementary file 6, Figure S6(a)).

To evaluate the effect of medicated serum on the inflammation induced by MSU crystal, we detected the release and gene expression of inflammatory cytokines. As shown in Figures 6(b)–6(i), the MSU crystal stimulation could significantly induce inflammatory cytokines cascade. And the addition of 40% blank serum could not notably decrease MSU-induced IL-1*β* and TNF-*α* release (Supplementary file 6 Figure S6(b)). However, treated with varying concentrations of the serum-containing WSP in MSU-activated macrophages, the mRNA expression and the release of IL-1*β*, TNF-*α*, and IL-6 were dramatically reduced in a concentration-dependent manner. As the positive control groups, colchicine and etoricoxib exhibited powerful anti-inflammatory activity. However, there was no prominent difference between the model group and medicated serum group for the mRNA expression of IL-18.

### 3.8. WSP Downregulated the Protein Expression of MAPK Signaling and NF-*κ*B Signaling Pathways in MSU-Activated THP-1 Macrophages

**Figure 7:**
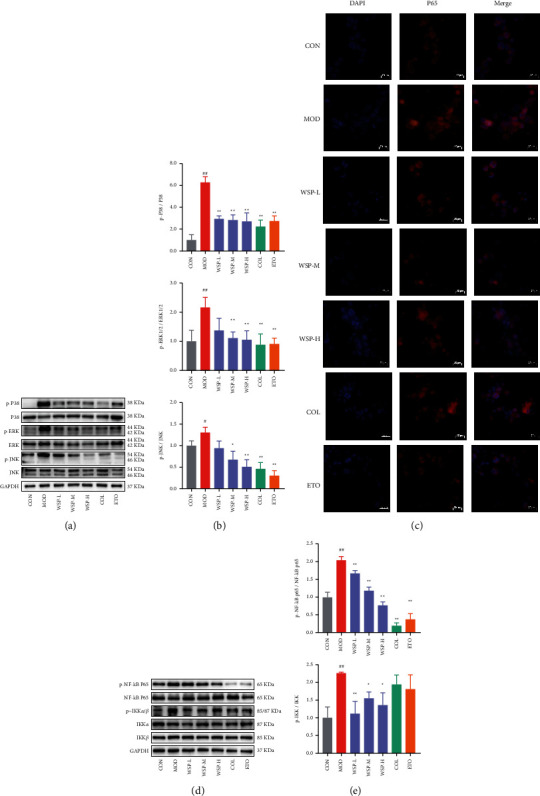
WSP downregulated the protein expression of MAPK/NF-*κ*B signaling pathways in MSU-activated THP-1 macrophages. (a, b) Levels of p-P38, p-ERK, p-JNK, P38, ERK, and JNK in MSU-activated THP-1 macrophages were analyzed by western blot. Values are mean ± SD (*n* = 3). (c) The nuclear translocation activity of NF-*κ*B p65 in MSU-activated THP-1 macrophages was analyzed by immunofluorescence staining. Values are mean ± SD (*n* = 3). (d, e) Levels of p-p65, p-IKK*α*/*β*, p65, IKK*α*, and IKK*β* in MSU-activated THP-1 macrophages were analyzed by western blot. Values are mean ± SD (*n* = 3). #*p* < 0.05, ##*p* < 0.01 vs. CON group; *∗p* < 0.05, ^*∗∗*^*p* < 0.01 vs. MOD group.

The NF-*κ*B and MAPK signaling pathways, as the important downstream pathways, played a crucial role in the MSU crystal-induced inflammatory [43]. According to the predicted results of the network pharmacology, TNF signaling was forecasted as the key pathway. Therefore, the activation of MAPK signaling and NF-*κ*B signaling pathways were measured by western blot and immunofluorescence staining. As depicted in Figures 7(a)–7(e), MSU crystal markedly activated the MAPK signaling and NF-*κ*B signaling, manifested as up-regulated phosphorylation of P38, ERK 1/2, and JNK 1/2/3, IKK*α*/*β*, and NF-*κ*B p65, compared to the control group whereas WSP treatment could significantly inhibit the phosphorylation of the above proteins in a concentration-dependent manner. Moreover, western blot assays of p-P38, p-JNK, and p-P65 showed that 40% blank serum could not inhibit these phosphorylated proteins' expression (Supplementary file 6 Figures S6(c) and S6(d)). Furthermore, the figures of immunofluorescence staining revealed the inhibition effect of WSP on the nuclear translocation of NF-*κ*B p65 (Figure 7(c)). Similarly, colchicine and etoricoxib treatment could restore the MAPK signaling and NF-*κ*B signaling to baseline. In conclusion, our data demonstrated the suppressive effect of WSP on MAPK signaling and NF-*κ*B signaling, thus downregulating the gene expression and release of inflammation cytokines in response to MSU insult.

### 3.9. WSP Suppressed NLRP3 Inflammasome Activation in MSU-Activated THP-1 Macrophages

**Figure 8:**
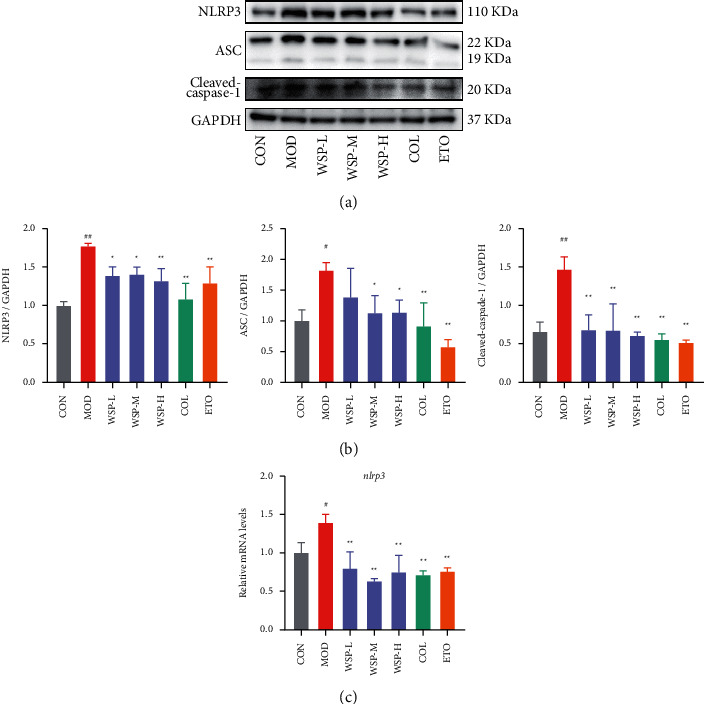
WSP suppressed NLRP3 inflammasome activation in MSU-activated THP-1 macrophages. (a, b) Levels of NLRP3, ASC, and cleaved Caspase-1 in MSU-activated THP-1 macrophages were analyzed by western blot. Values are mean ± SD (*n* = 3). (c) Relative mRNA levels of NLRP3 of MSU-activated THP-1 macrophages measured by qRT-PCR. Values are mean ± SD (*n* = 3). #*p* < 0.05, ##*p* < 0.01 vs. CON group; *∗p* < 0.05, ^*∗∗*^*p* < 0.01 vs. MOD group.

In general, the assembly of NLRP3 inflammasome was essential for the maturation of IL-1*β*, which triggered the inflammatory amplification reaction via binding to IL-1R. As mentioned above, WSP markedly inhibited MSU-induced IL-1*β* secretion in a concentration-dependent manner. Thus, the expression of NLRP3 inflammasome-associated proteins was investigated by western blot. In primed MSU-induced THP-1 macrophage, NLRP3 inflammasome was activated, as evidenced by remarkable up-regulated protein expression of NLRP3, ASC, and cleaved Caspase-1. However, 30% and 40% serum-containing WSP could notably downregulate the protein expression of NLRP3 inflammasome-associated proteins (Figures 8(a) and 8(b)). And the blank serum had no effect on the expression of NLRP3, ASC, and cleaved Caspase-1 (Supplementary file 6 Figures S6(c) and S6(d)). Moreover, WSP could dramatically inhibit MSU-stimulated mRNA expression of NLRP3 in THP-1 macrophages (Figure 8(c)). Likewise, the inhibitory activity of NLRP3 inflammasome was observed in the colchicine-treated and etoricoxib-treated groups. Thus, we concluded that WSP could inhibit NLRP3 inflammasome signaling at protein and gene expression levels, followed by decreased secretion of activated IL-1*β*.

## 4. Discussion

Gout is one of the most common inflammatory arthritis which is associated with the inflammation of diarthrodial joints triggered by MSU crystal. Gouty flare is closely related to the secretion of proinflammatory cytokines and chemokines induced by MSU crystals in inflammasome-dependent and independent mechanisms, including mitochondrial dysfunction, reactive oxygen species (ROS), lysosomal damage, ionic flux, and AMP-activated protein kinase (AMPK) [44, 45]. WSP exhibited anti-inflammatory, antinociceptive, urate-lowering, and antigout activity, which has been applied in various arthritis among Tibetans in China for centuries [46–48]. However, the specific mechanisms of WSP against gouty arthritis have rarely been reported. Considering the complicated features of TCM which is characterized by complex components and multiple targets, we used the network pharmacology method to predict the material basis and potential mechanisms of WSP and utilized molecular docking and serum pharmacology method to preliminarily confirm the results of the prediction.

In this work, we obtained 88 ingredients by searching the database and ADME screening. 13 of them with a high degree (degree >20) were regarded as potential active ingredients against gout. 5-Cis-Cyclopentadecen-1-One, 5-Cis-Cyclotetradecen-1-One, and Androstenedione are the compounds from Moschus, which is a rare and precious medicine in TCM. As the main active ingredient of Moschus, Muscone exhibits a broad spectrum of biological properties, including anti-inflammatory, antioxidant, antiapoptotic, and antitumor activities. Similar to the structure of muscone, 5-Cis-Cyclopentadecen-1-One and 5-Cis-Cyclotetradecen-1-One are supposed to be analogical pharmacological activity as Muscone [49, 50]. Artemisia Ketone, Beta-Ionone, and (E)-6,10-Dimethyl-9-methylene-5-undecen-2-one are three ketone ingredients from *Aucklandia lappa* Decne. It has been reported that Artemisia Ketone, as the major oily content extracted from *Achillea millefolium* L., performed anti-inflammatory and antioxidant activities in LPS-induced RAW 264.7 macrophages [51]. Recently, Beta-Ionone was confirmed to exhibit anti-inflammatory activity in LPS-induced BV2 microglial cells and *Cutibacterium acnes*-induced THP-1 monocytes via inhibiting NF-*κ*B and MAPK pathways and decreasing the release of proinflammatory mediators, such as IL-1*β*, NO, PEG_2_, and TNF-*α* [52]. Plenty of constituents in *Acorus calamus* L., including (−)-isoshyobunone, Acolamone, Calacone, Isoacolamone, and others, were predicted pharmacological activities in this study. (−)-isoshyobunone was regarded as a potential anti-inflammatory and antioxidant compound, which was the main component of volatile oil constituents of *Lactuca serriola* L., according to Abd-ElGawad's research [53]. Despite we predicted abundant components, whether these components acted as prototypes or metabolites was still unclear. In further research, the predicted components will be arranged to evaluate the antigout activity. Besides, the serum phytochemical method will be adopted to explore the main active ingredients in plasma, thus illustrating the material basis of WSP.

The modules of the PPI network are core functional areas that are predicted as crucial targets. In this paper, a total of 8 modules were found. According to the GO analysis, the first module with the most density was the possible targets for the treatment of the disease. The main biological processes (Table 3) were prescribed as “leukocyte differentiation,” “positive regulation of cytokine production,” “response to lipopolysaccharide,” “positive regulation of cell-cell adhesion,” and “regulation of inflammatory response,” which were associated with the initiation, occurrence, and development of inflammation [54]. In GA, the inflammatory response is principally mediated by monocytes/macrophages and neutrophils. Resident macrophages trigger inflammation by mediating urate crystal phagocytosis, releasing a variety of proinflammatory cytokines and chemokines. Subsequently, neutrophils and other immune cells were recruited to the site of MSU crystal deposition, resulting in the production of proinflammatory substances and local acute immune reactions whereas neutrophils play a major role in the resolution of acute gout, suspending the inflammatory process through the formation of neutrophil extracellular traps (NETs) [54]. Thus, the results of GO analysis indicated that WSP could regulate the response of innate immune cells.

Moreover, TNF signaling, HIF-1 signaling, and Jak-STAT signaling were predicted as the top 3 pathways. In TNF signaling, TNF-*α* is an inflammatory mediator in gouty arthritis [55], which could be released by MSU crystal stimulation. Meanwhile, TNF-*α* could not only promote the activation of Caspase-1 and assemble the NLRP3 inflammasome but also advance the expression of IL-1*β* mRNA [56, 57]. Certainly, TNF-*α* is a potent physiological inducer of NF-*κ*B and MAPK signaling pathways [58, 59]. In the HIF-1 signaling, it was reported that hypoxia-inducible factor-1*α* (HIF-1*α*) was associated with the glucose metabolism process in the inflammatory cells [60]. Recently, evidence showed that there was a direct connection between HIF-1*α* and NLRP3 pathways in the hypoxic and inflammatory state which demonstrated that HIF-1*α* facilitated the production of IL-1*β* by mediating NLRP3 pathways [61, 62]. Moreover, inhibition of HIF-1*α* by Chaetocin could contribute to the suppression of NLRP3 inflammasome activation and IL-1*β* secretion in the MSU-induced gouty model [63]. These researches implicated that HIF signaling could be a new strategy for gout treatment. It has been acknowledged that Jak-STAT signaling was related to immune-mediated diseases and IL-6 signaling [64]. Yang et al.'s findings indicated that berberine exerted anti-inflammatory activity by inhibiting the Jak-STAT signaling pathway [65]. And another recent document illustrated that the phosphorylation levels of JAK2 and STAT3 were upregulated in the kidney of hyperuricemia mice [66]. Furthermore, the promotion of p-JAK2 and p-STAT3 was observed in the synovial tissues of MSU crystal-induced arthritis rats according to Yang's study [67]. At present, Jaks and STATs have become attractive pharmacological targets to treat immune-related diseases [68]. As we know, GA is a self-limited disease caused by the immune system after MSU crystal stimulation. Thus, regulators targeting for Jaks-STATs signaling might be an interesting research direction for gout treatment.

TNF signaling is a significant part of the inflammatory process, and TNF-*α* plays a determinate role in the pathogenesis of several inflammatory diseases such as rheumatoid arthritis (RA), psoriatic arthritis, and ankylopoetica spondylarthritis [69]. It has been reported that TNF-*α* monoclonal antibody Infliximab and Adamuzumab were applied in RA patients and also benefit refractory gout patients [70, 71]. Hence, the inhibitors of TNF-*α* could contribute to the remission of gout flares. Considering that TNF signaling was the top predicted pathway, therefore, the downstream pathways of TNF signaling, namely, NF-*κ*B, MAPK, and pivotal NLRP3 inflammasome signaling pathways were adopted to verify the antigout activity of WSP in further trials.

The molecule docking method was applied to evidence the effect of ingredients on NF-*κ*B p65, NLRP3, IL-1*β*, TNF-*α*, and p38 MAPK. The results suggested that most components showed an affinity for the above targets. Remarkably, all 14 ingredients could dock well with TNF-*α* (bind energy ≤ -5.5 kCal/mol). It implicated the anti-inflammatory effect of WSP on TNF signaling which was in accordance with the results of network pharmacology. In the following study, *in vitro* experiment proved the above findings.

NF-*κ*B is an essential transcription factor associated with immunity, inflammation, and cell death, which is a switch of the inflammatory response to infection or stimuli [72]. In gouty arthritis, MSU crystal stimulation could prime macrophages triggering Toll-like receptor (TLR) 2 or TLR4 as the signal 1 and activating the NF-*κ*B [55]. In short, the upstream I*κ*B kinase (IKK) of p65 will autophosphorylate to activate the I*κ*B inhibitor family (I*κ*B*α*, I*κ*B*β*, and I*κ*B*ε*) to be phosphorylated, ubiquitination, and degradation. Then, the released NF-*κ*B dimers (p50 and p65) can enter the nucleus and bind to the DNA to regulate the gene expression of inflammatory mediators, such as IL-1*β*, IL-6, TNF-*α*, and NLRP3 [73, 74], which was consistent with our data. In addition, the MAPK signaling regulates the proliferation, apoptosis, inflammation, and innate immunity including JNK, p38, and ERK which are associated with MSU crystal-induced gouty arthritis [75]. Early studies have confirmed that ROS could induce MAPKs to facilitate the activation of NLRP3 inflammasome [76, 77]. Moreover, it was reported that the members of the MAPK family played a crucial role in the activation process of NF-*κ*B such as the IKK activation and I*κ*B degradation [78, 79]. Our results showed that MSU crystal could activate the NF-*κ*B and MAKP signaling pathways, evidenced by increased phosphorylation of NF-*κ*B P65, IKK p38, JNK, and ERK whereas WSP could inhibit MAPK signaling and NF-*κ*B signaling pathways activation and gene expression of inflammatory mediators in a concentration-dependent manner. Notably, we subsequently noted that WSP blocked the MSU crystal-induced nuclear translocation of p65 according to the immunocytochemical analysis. Conversely, IL-18 inhibitory action has not been observed in WSP and positive control groups. It has been assumed that WSP could not block the expression of IL-18 mRNA, but it might work on the processes of assembly, modification, and secretion according to the results of ELISA. Taken together, these data revealed that WSP suppressed MSU-induced inflammation by modulating MAPK signaling and NF-*κ*B signaling pathways.

As the initiator of gouty inflammation, mature IL-1*β* is produced and secreted to combat infection and stimulation via recruiting monocytes/macrophages and neutrophils to the injured joints. Massive IL-1*β* release could result in other cytokine cascades, aggravation of inflammation, and joint swelling [80]. In our present study, we assessed the anti-inflammatory activity of WSP in MSU crystal-induced THP-1 macrophages. THP-1 is a common model cell to estimate macrophage activities and MSU crystal stimulation is a typical model of gouty inflammatory [81–83]. Sustained stimulation of MSU crystal led to a significant proinflammatory increase of cytokines, including IL-1*β*, TNF-*α*, IL-18, and IL-6; this result indicated the massive release of outside the cells is a remarkable event, which is in accordance with previous studies [83, 84]. However, WSP-containing serum treatment (20%, 30%, and 40%) could remarkably inhibit the production of the above proinflammatory cytokines respond to the MSU crystal challenge (Figure 6), which suggested that WSP could reduce the MSU crystal-induced cytokines storm.

As mentioned above, IL-1*β* is a pivotal cytokine in acute gout and its maturation is associated with NLRP3 inflammasome assembly. NLRP3 is a member of the nucleotide-binding oligomerization domain (NOD)-like receptor family which binds ASC and procaspase-1 to form inflammasome [85]. It is well-accepted that stimuli such as ATP or MSU crystal can trigger the assembly of the inflammasome via NF-*κ*B and MAPK signaling pathways, resulting in procaspase-1 autocleavage which can cleave the proinflammatory cytokines (pro-IL-1*β* and pro-IL-18) to produce the mature IL-1*β* and IL-18 [85]. In view of the potent inhibitory effect of WSP on IL-1*β*, we assessed the expressions of targets in NLRP3 signaling by qPCR and western blot. The results showed that the mRNA level of NLRP3 and protein levels of NLRP3, cleaved Caspase-1, and ASC significantly decrease after WSP treatment. Therefore, these results indicated that WSP attenuated inflammation by inhibiting the activation of NLRP3 inflammasome.

It has been well-recognized that serum-containing drugs after treatment in experimental animals contain not only the drug prototype but also the metabolites, which mimic the actual composition of TCM entering into circulation [23]. Although WSP-containing-serum was applied to explore the pharmacological mechanism of WSP on gouty inflammation, the active components of WSP-containing-serum were not clarified in the present study. Thus, which active ingredients or active metabolites contributed to the antigout potency of WSP should be further verified by the methods of serum pharmacochemistry and pharmacokinetics of TCM. Although our findings showed that WSP could reduce the release of IL-1*β*, we did not investigate the effects and mechanisms of WSP on IL-1*β* release. The activation of NLRP3 inflammasome and release of mature IL-1*β* is a complex process. We noted that the downstream Gasdermin D of cleaved Caspase-1 and Caspase-11 can induce pyroptosis which amplifies the inflammatory response and facilitates the release of cytokines [54]. Whether WSP could inhibit pyroptosis remains to be studied in the future. Furthermore, the components with high affinity for the pivotal targets revealed by the molecule docking should be validated by biomolecular interaction analysis.

## 5. Conclusions

In summary, we firstly constructed an ingredient-target-pathway network to decipher the possible mechanisms of WSP against gouty arthritis based on network pharmacology and molecule docking methods. Then, *in vitro* experiments demonstrated that WSP-containing-serum could inhibit the release of inflammatory cytokines in MSU-stimulated THP-1 macrophages. And further studies showed that WSP could downregulate protein phosphorylation or expression of MAPK signaling pathway (p-P38, p-JNK, and p-ERK), NF-*κ*B signaling pathway (p-P65 and p-IKK), and NLRP3 signaling pathway (NLRP3, ASC, and cleaved Caspase-1). Our findings explain the multiple therapeutic mechanisms of WSP against gouty arthritis and suggest that WSP could be a prospective candidate as a novel antigout agent for further investigation.

## Figures and Tables

**Table 1:** All herbal ingredients of WSP.

Chinese name	Latin name	Marker
Zang Chang Pu	*Acorus calamus* L.	ZCP
He Zi	*Terminalia chebula* Retz.	HZ
Mu Xiang	*Aucklandia lappa* Decne.	MX
She Xiang (artificial Moschus in English)	Not applicable	SX
Tie Bang Chui	*Aconitum pendulum* Busch	TBC

**Table 2:** Primers used for qRT-PCR.

Gene	Forward sequence (5′–3′)	Reverse sequence (5′–3′)
IL-6	CACTGGTCTTTTGGAGTTTGAG	GGACTTTTGTACTCATCTGCAC
IL-1*β*	GCCAGTGAAATGATGGCTTATT	AGGAGCACTTCATCTGTTTAGG
TNF-*α*	AGCCCTGGTATGAGCCCATCTATC	TCCCAAAGTAGACCTGCCCAGAC
IL-18	GCTGAAGATGATGAAAACCTGG	CAAATAGAGGCCGATTTCCTTG
NLRP3	AGGGATGAGAGTGTTGTGTGAAACG	GCTTCTGGTTGCTGCTGAGGAC
*β*-Actin	AATCTGGCACCACACCTTCTACAA	GGATAGCACAGCCTGGATAGCAA

**Table 3:** The function description of the PPI network.

GO	Description	−Log10(P)
GO:0002521	Leukocyte differentiation	23.16
GO:0001819	Positive regulation of cytokine production	22.42
GO:0032496	Response to lipopolysaccharide	18.96
GO:0022409	Positive regulation of cell-cell adhesion	18.13
GO:0050727	Regulation of inflammatory response	17.73

**Table 4:** Targets-pathways enrichment results.

GO	Pathway	Log10(P)	Targets
ko04668	TNF signaling pathway	−14.52	CSF2, FOS, IL1B, JUN, LTA, PIK3CA, PIK3R1, MAPK3, PTGS2, CCL2, CCL5, TNF, VCAM1, RIPK1
hsa04066	HIF-1 signaling pathway	−13.92	BCL2, EPO, GAPDH, IFNG, IGF1R, INS, INSR, NOS2, PIK3CA, PIK3R1, PRKCA, MAPK3, RPS6KB1, STAT3
ko04630	Jak-STAT signaling pathway	−3.56	BCL2, CSF2, EPO, IFNG, IL4, IL6ST, IL10, IL13, JAK3, LEP, PIK3CA, PIK3R1, PTPN6, STAT3, STAT5A
hsa05163	Human cytomegalovirus infection	−13.10	CALR, IL1B, ITGB3, PIK3CA, PIK3R1, PRKCA, MAPK3, PTGER1, PTGS2, RNASE1, RPS6KB1, CCL2, CCL5, SRC, STAT3, TNF, RIPK1
ko04657	IL-17 signaling pathway	−12.49	CSF2, FOS, IFNG, IL1B, IL4, IL13, JUN, MAPK3, PTGS2, S100A9, CCL2, TNF
ko04152	AMPK signaling pathway	−12.49	FBP1, HNF4A, IGF1R, INS, INSR, LEP, PIK3CA, PIK3R1, PPARG, RPS6KB1, SCD, ADIPOQ, SIRT1
ko04060	Cytokine-cytokine receptor interaction	−12.26	CSF2, EPO, IFNG, IL1B, IL4, IL6ST, IL10, IL13, KIT, LEP, LTA, PF4, CCL2, CCL5, TGFB1, TNF, TNFSF11
ko04659	Th17 cell differentiation	−11.75	FOS, IFNG, IL1B, IL4, IL6ST, JAK3, JUN, MAPK3, STAT3, STAT5A, TGFB1, FOXP3
hsa04380	Osteoclast differentiation	−11.66	FOS, IFNG, IL1B, ITGB3, JUN, PIK3CA, PIK3R1, PPARG, MAPK3, RNASE1, TGFB1, TNF, TNFSF11
ko05321	Inflammatory bowel disease	−11.28	IFNG, IL1B, IL4, IL10, IL13, JUN, STAT3, TGFB1, TNF, FOXP3
ko05323	Rheumatoid arthritis	−11.23	CSF2, FOS, IFNG, IL1B, ITGB2, JUN, CCL2, CCL5, TGFB1, TNF, TNFSF11
hsa04917	Prolactin signaling pathway	−10.64	CYP17A1, FOS, INS, PIK3CA, PIK3R1, MAPK3, SRC, STAT3, STAT5A, TNFSF11
ko04660	T Cell receptor signaling pathway	−10.57	CSF2, FOS, IFNG, IL4, IL10, JUN, PIK3CA, PIK3R1, MAPK3, PTPN6, TNF
hsa01521	EGFR tyrosine kinase inhibitor resistance	−10.41	BCL2, FGFR2, IGF1R, PIK3CA, PIK3R1, PRKCA, MAPK3, RPS6KB1, SRC, STAT3
hsa04015	Rap1 signaling pathway	−10.04	ACTB, FGFR2, IGF1R, INS, INSR, ITGB2, ITGB3, KIT, PIK3CA, PIK3R1, PRKCA, MAPK3, RNASE1, SRC
hsa04151	PI3K-Akt signaling pathway	−9.92	BCL2, EPO, FGFR2, IGF1R, IL4, INS, INSR, ITGB3, JAK3, KIT, PIK3CA, PIK3CG, PIK3R1, PRKCA, MAPK3, RNASE1, RPS6KB1
hsa04664	Fc epsilon RI signaling pathway	−8.95	CSF2, IL4, IL13, PIK3CA, PIK3R1, PRKCA, MAPK3, RNASE1, TNF
hsa04620	Toll-like receptor signaling pathway	−8.93	FOS, IL1B, JUN, PIK3CA, PIK3R1, MAPK3, RNASE1, CCL5, TNF, RIPK1
hsa04062	Chemokine signaling pathway	−8.51	JAK3, PF4, PIK3CA, PIK3CG, PIK3R1, PRKCA, MAPK3, RNASE1, CCL2, CCL5, SRC, STAT3
hsa04012	ErbB signaling pathway	−8.32	ABL1, JUN, PIK3CA, PIK3R1, PRKCA, MAPK3, RPS6KB1, SRC, STAT5A

**Table 5:** Parameters of main ingredients.

Marker	Ingredient	Degree	Betweenness centrality	Closeness centrality
SX10	5-Cis-Cyclopentadecen-1-One	22	0.0193688	0.43356643
SX26	5-Cis-Cyclotetradecen-1-One	22	0.0193688	0.43356643
ZCP8	(−)-isoshyobunone	22	0.0193688	0.43356643
MX1	Artemisia Ketone	21	0.01401109	0.42465753
MX30	Beta-Ionone	21	0.01401109	0.42465753
MX5	(E)-6,10-Dimethyl-9-methylene-5-undecen-2-one	21	0.01401109	0.42465753
SX1	Androstenedione	21	0.01401109	0.42465753
ZCP12	Acolamone	21	0.01401109	0.42465753
ZCP20	Calacone	21	0.01401109	0.42465753
ZCP23	Isoacolamone	21	0.01401109	0.42465753
ZCP4	Shyobunone	21	0.01401109	0.42465753
ZCP5	Acoragermacrone	21	0.01401109	0.42465753
ZCP6	Acoronene	21	0.01401109	0.42465753
SX5	Estradiol	15	0.04337996	0.38509317
SX2	Testosterone	10	0.02274873	0.36686391
SX3	3,5-Dihydroxybenzoic acid	10	0.02185115	0.37575758

**Table 6:** Parameters of main targets.

Target	Degree	Betweenness centrality	Closeness centrality
PIK3R1	29	0.05908793	0.47509579
PIK3CA	28	0.05208823	0.47148289
SRC	26	0.04916463	0.43816254
INS	25	0.06397996	0.43205575
IL10	25	0.03851927	0.4012945
TGFB1	25	0.04392304	0.42611684
TNF	24	0.03075795	0.42906574
FGFR2	24	0.04953984	0.41196013
PF4	23	0.02872017	0.39616613
SIRT1	22	0.03333712	0.38871473
TNFSF11	22	0.02359378	0.39871383
IL4	21	0.01637895	0.39871383
CSF2	20	0.0155255	0.4012945
IL1B	18	0.078045	0.4012945
STAT5A	18	0.01173282	0.39116719

## Data Availability

The human data used to support the findings of this study have been deposited as follows: BATMAN-TCM, http://bionet.ncpsb.org.cn/batman-tcm/, SwissADME, http://www.swissadme.ch/, UniProt, https://www.uniprot.org/, OMIM®, https://omim.org/, Drugbank, https://go.drugbank.com/, GeneCards®, https://www.genecards.org/, TTD, http://db.idrblab.net/ttd/, STRING, https://string-db.org/, and Metascape, https://metascape.org/gp/index.html#/main/step1.

## Supplementary Materials

Supplementary Materials Supplementary file 1. Table S1-1: effect of WSP on foot swelling in MSU crystal-induced acute gouty arthritis model. Table S1-2: effect of WSP on IL-1*β* in MSU crystal-induced acute gouty arthritis model. Supplementary file 2. Figure S2: a herb-ingredient-target network of WSP. Supplementary file 3. Table S3: prediction of ingredients and targets of WSP. Supplementary file 4. Table S4: targets information on WSP and gout. Supplementary file 5. Figure S5: PPI network. Supplementary file 6. Figure S6: the effect of blank serum.
