# Supplementary material for: Mechanism Investigation of Wuwei Shexiang Pills on Gouty Arthritis via Network Pharmacology, Molecule Docking, and Pharmacological Verification
Source: Evid Based Complement Alternat Med. 2022 Oct 7;2022:2377692. doi: 10.1155/2022/2377692 (PMC9568303; doi:10.1155/2022/2377692)
Supplement: Supplementary Materials — Supplementary file 1. Table S1-1: effect of WSP on foot swelling in MSU crystal-induced acute gouty arthritis model. Table S1-2: effect of WSP on IL-1β in MSU crystal-induced acute gouty arthritis model. Supplementary file 2. Figure S2: a herb-ingredient-target network of WSP. Supplementary file 3. Table S3: prediction of ingredients and targets of WSP. Supplementary file 4. Table S4: targets information on WSP and gout. Supplementary file 5. Figure S5: PPI network. Supplementary file 6. Figure S6: the effect of blank serum. [file 2377692.f1.docx]

**Supplementary file 1**

**Table S1-1** Effect of WSP on foot swelling in MSU crystal-induced acute gouty arthritis model. (*$\overline{x}$* ± SD, n = 10~12)

| Group | Dose | Foot swelling (mL) | | | | | |
| --- | --- | --- | --- | --- | --- | --- | --- |
|  | (mg/kg) | 2h | 4h | 6h | 8h | 24h | 48h |
| Control | - | 0.1350±0.1446 | 0.0623±0.1921^**^ | 0.1236±0.1856^**^ | 0.1550±0.2006^**^ | 0.0773±0.2441^**^ | -0.0650±0.0872^**^ |
| Model | - | 0.1927±0.1940 | 0.3882±0.1335 | 0.4727±0.1546 | 0.4455±0.1627 | 0.4709±0.0810 | 0.1765±0.2066 |
| Colchicine | 0.48 | 0.2527±0.3007 | 0.3195±0.2946 | 0.3709±0.2450 | 0.4277±0.2692 | 0.2655±0.2579^*^ | 0.0391±0.2611 |
| WSP-L | 20 | 0.1909±0.1807 | 0.2423±0.1576^*^ | 0.3345±0.2203 | 0.4632±0.1909 | 0.3100±0.1890 | -0.0291±0.1497^*^ |
| WSP-H | 40 | 0.1746±0.1295 | 0.3329±0.1986 | 0.3858±0.2077 | 0.3708±0.1390 | 0.2988±0.1509^**^ | -0.0192±0.1870^*^ |

Note: ^*^*P* ﹤0.05, ^**^*P* ﹤0.01 vs. MOD group.

**Table S1-2** Effect of WSP on IL-1β in MSU crystal-induced acute gouty arthritis model. (*$\overline{x}$* ± SD, n = 10~12)

| Group | Dose (mg/kg) | IL-1β of synovial fluid (pg/mL) | IL-1β of serum (pg/mL) |
| --- | --- | --- | --- |
| Control | - | 207±123^**^ | 742±147^*^ |
| Model | - | 364±48 | 925±146 |
| Colchicine | 0.48 | 337±58 | 821±167 |
| WSP-L | 25 | 376±125 | 878±388 |
| WSP-H | 50 | 302±73^*^ | 728±128^**^ |

Note: ^*^*P* ﹤0.05, ^**^*P* ﹤0.01 vs. MOD group.


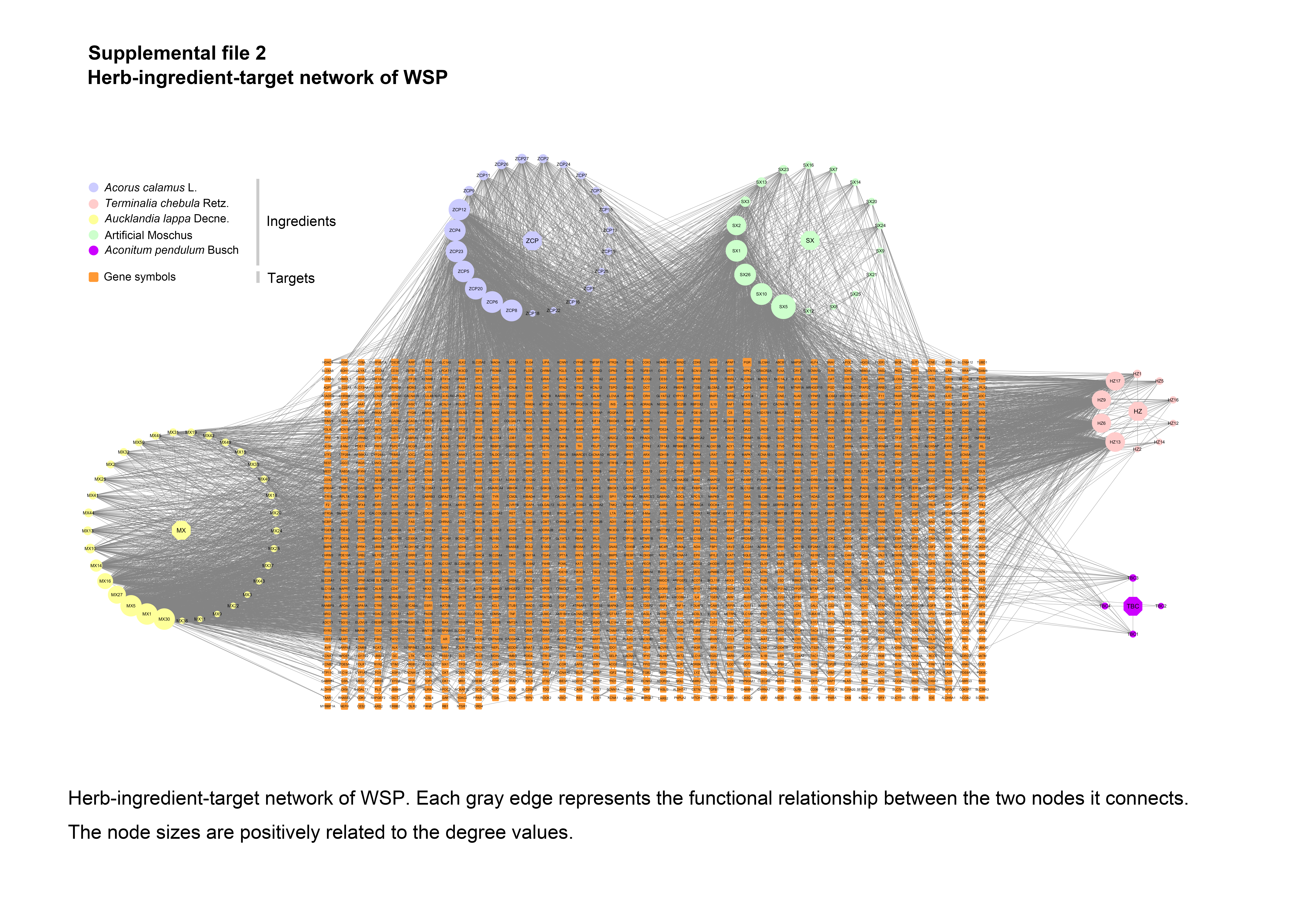


**Supplementary file 3**

**Table S3 Prediction of ingredients and targets of WSP**

| **Symbol** | **Ingredient** | **Targets** |
| --- | --- | --- |
| ZCP1 | Bisasaricin | PDE10A, PDE1B, CAMK2G, PDE4B, PLN, PDE11A, PDE4D, HRC, PDE5A, CAMK2D, PDE9A, PDE2A |
| ZCP2 | Borneol | TRPV3, ESRRG, AKR1C2, FECH, COX6B1, COX8A, ADRA2A, BNIP3, TACR2, NEFH, OPRK1, COX6C, COX5A, COX4I1, FABP6, NR1H4, TRPV1, CRH, AQP1, CRHR2, TRPM8, COX5B, COX3, PLA2G1B, CES1, ACTN3, FGF10, NEFL, TAC4, TRPA1, COX7C, AR, COX6A2, COX7B, TAC1, VCP, UCN2, CDH3, KCNK4, COX1, COX7A1, ADH1C, COX2, NGFR, HIF1A, SMO, CBFA2T3 |
| ZCP3 | Isocembrol | ESR1, PHB, ESR2, FGFR2, GPER1, TRIM24, PGR, TOX3, NEDD4, SRC, WNT5A, WNT4, TGFB1, SHH, UBR5, SOX9, TFAP2C, MED1, SERPINB3, AREG, PDGFB, TNFSF11, NKX3-1, LEF1, CD34, VDR |
| ZCP4 | Shyobunone | PDE7B, CYP17A1, PRKDC, PIK3CA, PIK3CB, ITPR3, ATM, CYP19A1, PDE11A, PIK3R1, ADA, HMGA2, TAC1, FURIN, G6PD, ATP11C, SIRT2, TRIM28, ESR2, TP53, ADRA2A, CETN1, NEDD4, FOXP3, LEF1, SORCS3, ADAP2, VCP, GAS6, DNM3, XCL1, UTS2R, RAPGEF2, BDKRB2, INS, BNIP3, CRH, AVPR2, UCN2, ATP2A1, AHCYL1, CASQ1, IDO1, IGF2, EDA, SPR, SELP, TAS1R3, PAWR, CD63, VDR, PCSK6, RAG2, PDE5A, PDE3A, PIK3CD, ADORA2B, ITPR2, POLA2, PDE6B, PDE8B, PDE8A, WNT4, HAP1, TNFSF11, PHB, TOX3, GNAT1, KHDRBS1, SPI1, XRCC4, HCN4, GNAT3, YWHAE, NUDT9, AGT, CXCL13, PTK2B, ADRB1, LRRC8A, GHRL, C5, CHRNA3, HSP90AB1, SLC6A4, TET1, KCNH2, MTOR, ZFPM1, ZFP42, CAMK2D, ATP1A1, SMO, AQP1, FKBP1B, ATP2B4, HPS4, RYR2, GPER1, XRCC6BP1, NOS1, SOX9, CAMK2G, GNAS, TRIM24, NFX1, PDE9A, ADORA2A, ESR1, PDE1A, PDE1B, PDE4C, PDE4B, NT5E, NR3C2, TACR2, IDNK, NT5C1A, SIRT1, UTS2, SIX1, PPP3CB, HOMER1, MED1, EDN1, HELB, CETN2, PIK3CG, FER, ARID1A, LIG4, ADAM8, FGFR2, BCL11B, SREBF1, TCF3, HIBADH, HMGCR, C2CD5, MMP28, WNT10B, GATA3, PIK3R6, ADRA1B, RYR3, RAG1, TNFAIP3, CDC42, CTNNB1, TKT, CD34, CBFA2T3, PARP10, TREM1, BMP5, LAMP2, NCBP2, REST, TAS1R2, PDE4A, ITPR1, PDE3B, PDE4D, OPRK1, PDE10A, PDE2A, HDAC2, RINT1, AMPD3, HDAC1, ACTN3, DGKI, STUB1, QDPR, SYT2, SHANK3, NKX3-1, CACNA1A, COMT, POLA1, BMP2, SHH, FGF10, ADRBK1, VTI1A, AURKA, BCL2, DRD2, SRC, STAP1, HIPK2, CSF2, HIF1A, SLC18A3, UBR5, PF4, IL10, PPP3R1, RAB8B, TAC4, IL4, PGLS, DRD1, HSP90AA1, CALHM1, HPRT1, TAL1, PDGFB, NEFH, TPM1, CRHR2, RYR1, PDE6A, PGD, PGR, PDE7A, PDE1C, ADORA1, PDE6C, RIPK1, CX3CR1, TFAP2C, NOS1AP, FKBP1A, CTR9, TGFB1, SIX4, TAS1R1, UBE2B, STAT5A, SLC26A6, CRP, TNF, SERPINB3, KCNB1, MAOB, SLC44A4, MCM3, 8-Mar, PLN, LONP1, HRC, PPP3CA, TALDO1, KIT, ABCC4, AREG, ATP1A2, NEFL, SPX, DKK3, DPPA3, POLB, SLC9A3R1, ADAP1, LTA, WNT5A, AMICA1, ALAD, RTN2, GLYR1, PIK3R5, HCN2 |
| ZCP5 | Acoragermacrone | PDE7B, CYP17A1, PRKDC, PIK3CA, PIK3CB, ITPR3, ATM, CYP19A1, PDE11A, PIK3R1, ADA, HDAC1, ACTN3, DGKI, STUB1, QDPR, SYT2, SHANK3, NKX3-1, CACNA1A, COMT, POLA1, BMP2, SHH, FGF10, ADRBK1, VTI1A, AURKA, BCL2, DRD2, SRC, STAP1, HIPK2, CSF2, HIF1A, SLC18A3, UBR5, PF4, IL10, PPP3R1, RAB8B, TAC4, IL4, PGLS, DRD1, HSP90AA1, CALHM1, HPRT1, TAL1, PDGFB, NEFH, TPM1, CRHR2, PDE5A, PDE3A, PIK3CD, ADORA2B, ITPR2, POLA2, PDE6B, PDE8B, PDE8A, WNT4, HAP1, TFAP2C, NOS1AP, FKBP1A, CTR9, TGFB1, SIX4, TAS1R1, UBE2B, STAT5A, SLC26A6, CRP, TNF, SERPINB3, KCNB1, MAOB, SLC44A4, MCM3, 8-Mar, PLN, LONP1, HRC, PPP3CA, TALDO1, KIT, ABCC4, AREG, ATP1A2, NEFL, SPX, DKK3, DPPA3, POLB, SLC9A3R1, ADAP1, LTA, WNT5A, AMICA1, ALAD, RTN2, GLYR1, PIK3R5, HCN2, PDE9A, ADORA2A, ESR1, PDE1A, PDE1B, PDE4C, PDE4B, NT5E, NR3C2, TACR2, IDNK, HMGA2, TAC1, FURIN, G6PD, ATP11C, SIRT2, TRIM28, ESR2, TP53, ADRA2A, CETN1, NEDD4, FOXP3, LEF1, SORCS3, ADAP2, VCP, GAS6, DNM3, XCL1, UTS2R, RAPGEF2, BDKRB2, INS, BNIP3, CRH, AVPR2, UCN2, ATP2A1, AHCYL1, CASQ1, IDO1, IGF2, EDA, SPR, SELP, TAS1R3, PAWR, CD63, VDR, PCSK6, RAG2, PDE4A, ITPR1, PDE3B, PDE4D, OPRK1, PDE10A, PDE2A, HDAC2, RINT1, AMPD3, AR, TNFSF11, PHB, TOX3, GNAT1, KHDRBS1, SPI1, XRCC4, HCN4, GNAT3, YWHAE, NUDT9, AGT, CXCL13, PTK2B, ADRB1, LRRC8A, GHRL, C5, CHRNA3, HSP90AB1, SLC6A4, TET1, KCNH2, MTOR, ZFPM1, ZFP42, CAMK2D, ATP1A1, SMO, AQP1, FKBP1B, ATP2B4, HPS4, RYR2, GPER1, XRCC6BP1, NOS1, SOX9, CAMK2G, GNAS, TRIM24, NFX1, RYR1, PDE6A, PGD, PGR, PDE7A, PDE1C, ADORA1, PDE6C, RIPK1, CX3CR1, NR3C1, NT5C1A, SIRT1, UTS2, SIX1, PPP3CB, HOMER1, MED1, EDN1, HELB, CETN2, PIK3CG, FER, ARID1A, LIG4, ADAM8, FGFR2, BCL11B, SREBF1, TCF3, HIBADH, HMGCR, C2CD5, MMP28, WNT10B, GATA3, PIK3R6, ADRA1B, RYR3, RAG1, TNFAIP3, CDC42, CTNNB1, TKT, CD34, CBFA2T3, PARP10, TREM1, BMP5, LAMP2, NCBP2, REST, TAS1R2 |
| ZCP6 | Acoronene | CYP17A1, NR3C2, RYR1, PRKDC, ADORA2B, PDE1B, PDE10A, PDE2A, HDAC2, RIPK1, CX3CR1, PRLR, HMGA2, TAC1, FURIN, G6PD, ATP11C, SIRT2, TRIM28, ESR2, TP53, ADRA2A, CETN1, NEDD4, FOXP3, LEF1, SORCS3, ADAP2, VCP, GAS6, DNM3, XCL1, UTS2R, RAPGEF2, BDKRB2, INS, BNIP3, CRH, AVPR2, UCN2, ATP2A1, AHCYL1, CASQ1, IDO1, IGF2, EDA, SPR, SELP, TAS1R3, PAWR, CD63, VDR, PCSK6, RAG2, ESR1, PDE7B, PDE3A, PIK3CD, PDE1A, PDE7A, PDE1C, ADORA1, PDE6C, PIK3R1, ADA, NR3C1, TNFSF11, PHB, TOX3, GNAT1, KHDRBS1, SPI1, XRCC4, HCN4, GNAT3, YWHAE, NUDT9, AGT, CXCL13, PTK2B, ADRB1, LRRC8A, GHRL, C5, CHRNA3, HSP90AB1, SLC6A4, TET1, KCNH2, MTOR, ZFPM1, ZFP42, CAMK2D, ATP1A1, SMO, AQP1, FKBP1B, ATP2B4, HPS4, RYR2, GPER1, XRCC6BP1, NOS1, SOX9, CAMK2G, GNAS, TRIM24, NFX1, PGR, PDE5A, ADORA2A, PDE3B, PDE4D, ITPR3, ATM, CYP19A1, PDE11A, WNT4, HAP1, ANXA1, NT5C1A, SIRT1, UTS2, SIX1, PPP3CB, HOMER1, MED1, EDN1, HELB, CETN2, PIK3CG, FER, ARID1A, LIG4, ADAM8, FGFR2, BCL11B, SREBF1, TCF3, HIBADH, HMGCR, C2CD5, MMP28, WNT10B, GATA3, PIK3R6, ADRA1B, RYR3, RAG1, TNFAIP3, CDC42, CTNNB1, TKT, CD34, CBFA2T3, PARP10, TREM1, BMP5, LAMP2, NCBP2, REST, TAS1R2, AR, PDE9A, ITPR1, PGD, PIK3CB, POLA2, PDE6B, PDE8B, PDE8A, TACR2, IDNK, HDAC1, ACTN3, DGKI, STUB1, QDPR, SYT2, SHANK3, NKX3-1, CACNA1A, COMT, POLA1, BMP2, SHH, FGF10, ADRBK1, VTI1A, AURKA, BCL2, DRD2, SRC, STAP1, HIPK2, CSF2, HIF1A, SLC18A3, UBR5, PF4, IL10, PPP3R1, RAB8B, TAC4, IL4, PGLS, DRD1, HSP90AA1, CALHM1, HPRT1, TAL1, PDGFB, NEFH, TPM1, CRHR2, NOS1AP, FKBP1A, CTR9, TGFB1, SIX4, TAS1R1, UBE2B, STAT5A, SLC26A6, CRP, TNF, SERPINB3, KCNB1, MAOB, SLC44A4, MCM3, 8-Mar, PLN, LONP1, HRC, PPP3CA, TALDO1, KIT, ABCC4, AREG, ATP1A2, NEFL, SPX, DKK3, DPPA3, POLB, SLC9A3R1, ADAP1, LTA, WNT5A, AMICA1, ALAD, RTN2, GLYR1, PIK3R5, HCN2, TFAP2C, OPRK1, PDE4A, PDE6A, PIK3CA, ITPR2, PDE4C, PDE4B, NT5E, RINT1, AMPD3, SRD5A1 |
| ZCP7 | Calamendiol | ESR1, PTGFR, TOX3, NEDD4, SRC, WNT5A, PGR, VDR, TGFB1, SHH, UBR5, SOX9, WNT4, TFAP2C, MED1, SERPINB3, AREG, PDGFB, PTGER1, TNFSF11, NKX3-1, LEF1, CD34, TRIM24, PTGIR, PHB, ESR2, FGFR2, GPER1 |
| ZCP8 | (-)-isoshyobunone | PDE7B, CYP17A1, PRKDC, PIK3CA, PIK3CB, ITPR3, ATM, CYP19A1, PDE11A, PIK3R1, ADA, FADS2, HDAC1, ACTN3, DGKI, STUB1, QDPR, SYT2, SHANK3, NKX3-1, CACNA1A, COMT, POLA1, BMP2, SHH, FGF10, ADRBK1, VTI1A, AURKA, BCL2, DRD2, SRC, STAP1, HIPK2, CSF2, HIF1A, SLC18A3, UBR5, PF4, IL10, PPP3R1, RAB8B, TAC4, IL4, PGLS, DRD1, HSP90AA1, CALHM1, HPRT1, TAL1, PDGFB, NEFH, TPM1, CRHR2, PDE5A, PDE3A, PIK3CD, ADORA2B, ITPR2, POLA2, PDE6B, PDE8B, PDE8A, WNT4, HAP1, FADS1, TFAP2C, NOS1AP, FKBP1A, CTR9, TGFB1, SIX4, TAS1R1, UBE2B, STAT5A, SLC26A6, CRP, TNF, SERPINB3, KCNB1, MAOB, SLC44A4, MCM3, 8-Mar, PLN, LONP1, HRC, PPP3CA, TALDO1, KIT, ABCC4, AREG, ATP1A2, NEFL, SPX, DKK3, DPPA3, POLB, SLC9A3R1, ADAP1, LTA, WNT5A, AMICA1, ALAD, RTN2, GLYR1, PIK3R5, HCN2, PDE9A, ADORA2A, ESR1, PDE1A, PDE1B, PDE4C, PDE4B, NT5E, NR3C2, TACR2, IDNK, PTGS1, HMGA2, TAC1, FURIN, G6PD, ATP11C, SIRT2, TRIM28, ESR2, TP53, ADRA2A, CETN1, NEDD4, FOXP3, LEF1, SORCS3, ADAP2, VCP, GAS6, DNM3, XCL1, UTS2R, RAPGEF2, BDKRB2, INS, BNIP3, CRH, AVPR2, UCN2, ATP2A1, AHCYL1, CASQ1, IDO1, IGF2, EDA, SPR, SELP, TAS1R3, PAWR, CD63, VDR, PCSK6, RAG2, PDE4A, ITPR1, PDE3B, PDE4D, OPRK1, PDE10A, PDE2A, HDAC2, RINT1, AMPD3, SLC8A1, PTGS2, TNFSF11, PHB, TOX3, GNAT1, KHDRBS1, SPI1, XRCC4, HCN4, GNAT3, YWHAE, NUDT9, AGT, CXCL13, PTK2B, ADRB1, LRRC8A, GHRL, C5, CHRNA3, HSP90AB1, SLC6A4, TET1, KCNH2, MTOR, ZFPM1, ZFP42, CAMK2D, ATP1A1, SMO, AQP1, FKBP1B, ATP2B4, HPS4, RYR2, GPER1, XRCC6BP1, NOS1, SOX9, CAMK2G, GNAS, TRIM24, NFX1, RYR1, PDE6A, PGD, PGR, PDE7A, PDE1C, ADORA1, PDE6C, RIPK1, CX3CR1, TRPV1, ELOVL4, NT5C1A, SIRT1, UTS2, SIX1, PPP3CB, HOMER1, MED1, EDN1, HELB, CETN2, PIK3CG, FER, ARID1A, LIG4, ADAM8, FGFR2, BCL11B, SREBF1, TCF3, HIBADH, HMGCR, C2CD5, MMP28, WNT10B, GATA3, PIK3R6, ADRA1B, RYR3, RAG1, TNFAIP3, CDC42, CTNNB1, TKT, CD34, CBFA2T3, PARP10, TREM1, BMP5, LAMP2, NCBP2, REST, TAS1R2 |
| ZCP9 | 1-Allyl-2,4,5-Trimethoxy-Benzene | PDE4A, DHFR, PDE3B, PDE5A, INSR, DPYD, ADK, GAS6, HSP90AB1, ATIC, RAB8B, PARP10, HCN2, PDE3A, PDE10A, DMTN, PDE9A, P2RX2, HOXA5, IL1B, CMPK2, CHRNB2, AK9, HYAL2, CHRNB4, CACNA1C, PDE2A, KCNMA1, TBPL1, HTR2A, FBLN1, NT5M, PDE1B, SLC6A4, CDKN1A, DNMT3B, MGMT, TRDMT1, TYMS, ABCC2, DNMT3A, SIRT2, SLC22A6, P2RX3, DPYS, DUT, HIF1A, HSP90AA1, DTYMK, DHFRL1, SHMT1, FOLR2, STUB1, HCN4, ASNS, AURKA, CHRNA3, RAPGEF2, FOLR1, DNMT1, PDE11A |
| ZCP10**^*^** | Trans-Calamenene |  |
| ZCP11 | Beta-Asarone | PDE4A, DHFR, PDE3B, PDE5A, INSR, DPYD, ADK, GAS6, HSP90AB1, ATIC, RAB8B, PARP10, HCN2, PDE3A, PDE10A, DMTN, PDE9A, P2RX2, HOXA5, IL1B, CMPK2, CHRNB2, AK9, HYAL2, CHRNB4, CACNA1C, PDE2A, KCNMA1, TBPL1, HTR2A, FBLN1, NT5M, PDE1B, SLC6A4, CDKN1A, DNMT3B, MGMT, TRDMT1, TYMS, ABCC2, DNMT3A, SIRT2, SLC22A6, P2RX3, DPYS, DUT, HIF1A, HSP90AA1, DTYMK, DHFRL1, SHMT1, FOLR2, STUB1, HCN4, ASNS, AURKA, CHRNA3, RAPGEF2, FOLR1, DNMT1, PDE11A |
| ZCP12 | Acolamone | PDE7B, CYP17A1, PRKDC, PIK3CA, PIK3CB, ITPR3, ATM, CYP19A1, PDE11A, PIK3R1, ADA, HMGA2, TAC1, FURIN, G6PD, ATP11C, SIRT2, TRIM28, ESR2, TP53, ADRA2A, CETN1, NEDD4, FOXP3, LEF1, SORCS3, ADAP2, VCP, GAS6, DNM3, XCL1, UTS2R, RAPGEF2, BDKRB2, INS, BNIP3, CRH, AVPR2, UCN2, ATP2A1, AHCYL1, CASQ1, IDO1, IGF2, EDA, SPR, SELP, TAS1R3, PAWR, CD63, VDR, PCSK6, RAG2, PDE5A, PDE3A, PIK3CD, ADORA2B, ITPR2, POLA2, PDE6B, PDE8B, PDE8A, WNT4, HAP1, TNFSF11, PHB, TOX3, GNAT1, KHDRBS1, SPI1, XRCC4, HCN4, GNAT3, YWHAE, NUDT9, AGT, CXCL13, PTK2B, ADRB1, LRRC8A, GHRL, C5, CHRNA3, HSP90AB1, SLC6A4, TET1, KCNH2, MTOR, ZFPM1, ZFP42, CAMK2D, ATP1A1, SMO, AQP1, FKBP1B, ATP2B4, HPS4, RYR2, GPER1, XRCC6BP1, NOS1, SOX9, CAMK2G, GNAS, TRIM24, NFX1, PDE9A, ADORA2A, ESR1, PDE1A, PDE1B, PDE4C, PDE4B, NT5E, NR3C2, TACR2, IDNK, NT5C1A, SIRT1, UTS2, SIX1, PPP3CB, HOMER1, MED1, EDN1, HELB, CETN2, PIK3CG, FER, ARID1A, LIG4, ADAM8, FGFR2, BCL11B, SREBF1, TCF3, HIBADH, HMGCR, C2CD5, MMP28, WNT10B, GATA3, PIK3R6, ADRA1B, RYR3, RAG1, TNFAIP3, CDC42, CTNNB1, TKT, CD34, CBFA2T3, PARP10, TREM1, BMP5, LAMP2, NCBP2, REST, TAS1R2, PDE4A, ITPR1, PDE3B, PDE4D, OPRK1, PDE10A, PDE2A, HDAC2, RINT1, AMPD3, HDAC1, ACTN3, DGKI, STUB1, QDPR, SYT2, SHANK3, NKX3-1, CACNA1A, COMT, POLA1, BMP2, SHH, FGF10, ADRBK1, VTI1A, AURKA, BCL2, DRD2, SRC, STAP1, HIPK2, CSF2, HIF1A, SLC18A3, UBR5, PF4, IL10, PPP3R1, RAB8B, TAC4, IL4, PGLS, DRD1, HSP90AA1, CALHM1, HPRT1, TAL1, PDGFB, NEFH, TPM1, CRHR2, RYR1, PDE6A, PGD, PGR, PDE7A, PDE1C, ADORA1, PDE6C, RIPK1, CX3CR1, TFAP2C, NOS1AP, FKBP1A, CTR9, TGFB1, SIX4, TAS1R1, UBE2B, STAT5A, SLC26A6, CRP, TNF, SERPINB3, KCNB1, MAOB, SLC44A4, MCM3, 8-Mar, PLN, LONP1, HRC, PPP3CA, TALDO1, KIT, ABCC4, AREG, ATP1A2, NEFL, SPX, DKK3, DPPA3, POLB, SLC9A3R1, ADAP1, LTA, WNT5A, AMICA1, ALAD, RTN2, GLYR1, PIK3R5, HCN2 |
| ZCP13 | Camphor | ESRRG, AKR1C2, FECH, COX6B1, COX8A, COX6C, COX5A, COX4I1, FABP6, NR1H4, COX5B, COX3, PLA2G1B, CES1, COX7C, AR, COX6A2, COX7B, COX1, COX7A1, ADH1C, COX2 |
| ZCP14**^*^** | Alpha-Calacorene |  |
| ZCP15 | Isocalamendiol | ESR1, PHB, ESR2, FGFR2, GPER1, TRIM24, PGR, TOX3, NEDD4, SRC, WNT5A, WNT4, TGFB1, SHH, UBR5, SOX9, TFAP2C, MED1, SERPINB3, AREG, PDGFB, TNFSF11, NKX3-1, LEF1, CD34, VDR |
| ZCP16 | Isosinomenine | ESR2, SHH, OPRD1, ESR1, OPRK1, OPRM1, FGFR2, NEFL, WLS, NEFH |
| ZCP17**^*^** | Siaresinolic Acid |  |
| ZCP18 | Eugenol | TRPV3, TYR, OPRK1, TRPM8, TRPA1, CNR2, CNR1 |
| ZCP19 | Acorenone | ESRRG, AKR1C2, FECH, COX6B1, COX8A, COX6C, COX5A, COX4I1, FABP6, NR1H4, COX5B, COX3, PLA2G1B, CES1, COX7C, AR, COX6A2, COX7B, COX1, COX7A1, ADH1C, COX2 |
| ZCP20 | Calacone | CYP17A1, PDE7B, PDE3A, PIK3CD, PDE1A, PDE7A, PDE1C, ADORA1, PDE6C, PIK3R1, ADA, NR3C1, TNFSF11, PHB, TOX3, GNAT1, KHDRBS1, SPI1, XRCC4, HCN4, GNAT3, YWHAE, NUDT9, AGT, CXCL13, PTK2B, ADRB1, LRRC8A, GHRL, C5, CHRNA3, HSP90AB1, SLC6A4, TET1, KCNH2, MTOR, ZFPM1, ZFP42, CAMK2D, ATP1A1, SMO, AQP1, FKBP1B, ATP2B4, HPS4, RYR2, GPER1, XRCC6BP1, NOS1, SOX9, CAMK2G, GNAS, TRIM24, NFX1, ESR1, PDE5A, ADORA2A, PDE3B, PDE4D, ITPR3, ATM, CYP19A1, PDE11A, WNT4, HAP1, ANXA1, NT5C1A, SIRT1, UTS2, SIX1, PPP3CB, HOMER1, MED1, EDN1, HELB, CETN2, PIK3CG, FER, ARID1A, LIG4, ADAM8, FGFR2, BCL11B, SREBF1, TCF3, HIBADH, HMGCR, C2CD5, MMP28, WNT10B, GATA3, PIK3R6, ADRA1B, RYR3, RAG1, TNFAIP3, CDC42, CTNNB1, TKT, CD34, CBFA2T3, PARP10, TREM1, BMP5, LAMP2, NCBP2, REST, TAS1R2, PGR, PDE9A, ITPR1, PGD, PIK3CB, POLA2, PDE6B, PDE8B, PDE8A, TACR2, IDNK, HDAC1, ACTN3, DGKI, STUB1, QDPR, SYT2, SHANK3, NKX3-1, CACNA1A, COMT, POLA1, BMP2, SHH, FGF10, ADRBK1, VTI1A, AURKA, BCL2, DRD2, SRC, STAP1, HIPK2, CSF2, HIF1A, SLC18A3, UBR5, PF4, IL10, PPP3R1, RAB8B, TAC4, IL4, PGLS, DRD1, HSP90AA1, CALHM1, HPRT1, TAL1, PDGFB, NEFH, TPM1, CRHR2, OPRK1, PDE4A, PDE6A, PIK3CA, ITPR2, PDE4C, PDE4B, NT5E, RINT1, AMPD3, SRD5A1, TFAP2C, NOS1AP, FKBP1A, CTR9, TGFB1, SIX4, TAS1R1, UBE2B, STAT5A, SLC26A6, CRP, TNF, SERPINB3, KCNB1, MAOB, SLC44A4, MCM3, 8-Mar, PLN, LONP1, HRC, PPP3CA, TALDO1, KIT, ABCC4, AREG, ATP1A2, NEFL, SPX, DKK3, DPPA3, POLB, SLC9A3R1, ADAP1, LTA, WNT5A, AMICA1, ALAD, RTN2, GLYR1, PIK3R5, HCN2, NR3C2, RYR1, PRKDC, ADORA2B, PDE1B, PDE10A, PDE2A, HDAC2, RIPK1, CX3CR1, AR, HMGA2, TAC1, FURIN, G6PD, ATP11C, SIRT2, TRIM28, ESR2, TP53, ADRA2A, CETN1, NEDD4, FOXP3, LEF1, SORCS3, ADAP2, VCP, GAS6, DNM3, XCL1, UTS2R, RAPGEF2, BDKRB2, INS, BNIP3, CRH, AVPR2, UCN2, ATP2A1, AHCYL1, CASQ1, IDO1, IGF2, EDA, SPR, SELP, TAS1R3, PAWR, CD63, VDR, PCSK6, RAG2 |
| ZCP21**^*^** | Gamma-Camphorene |  |
| ZCP22 | Isocantlyine | CACNA2D1, LIPA, CES1, PTGFR, CES2, CES5A, CES3, ESD |
| ZCP23 | Isoacolamone | PDE7B, CYP17A1, PRKDC, PIK3CA, PIK3CB, ITPR3, ATM, CYP19A1, PDE11A, PIK3R1, ADA, HDAC1, ACTN3, DGKI, STUB1, QDPR, SYT2, SHANK3, NKX3-1, CACNA1A, COMT, POLA1, BMP2, SHH, FGF10, ADRBK1, VTI1A, AURKA, BCL2, DRD2, SRC, STAP1, HIPK2, CSF2, HIF1A, SLC18A3, UBR5, PF4, IL10, PPP3R1, RAB8B, TAC4, IL4, PGLS, DRD1, HSP90AA1, CALHM1, HPRT1, TAL1, PDGFB, NEFH, TPM1, CRHR2, PDE5A, PDE3A, PIK3CD, ADORA2B, ITPR2, POLA2, PDE6B, PDE8B, PDE8A, WNT4, HAP1, TFAP2C, NOS1AP, FKBP1A, CTR9, TGFB1, SIX4, TAS1R1, UBE2B, STAT5A, SLC26A6, CRP, TNF, SERPINB3, KCNB1, MAOB, SLC44A4, MCM3, 8-Mar, PLN, LONP1, HRC, PPP3CA, TALDO1, KIT, ABCC4, AREG, ATP1A2, NEFL, SPX, DKK3, DPPA3, POLB, SLC9A3R1, ADAP1, LTA, WNT5A, AMICA1, ALAD, RTN2, GLYR1, PIK3R5, HCN2, PDE9A, ADORA2A, ESR1, PDE1A, PDE1B, PDE4C, PDE4B, NT5E, NR3C2, TACR2, IDNK, HMGA2, TAC1, FURIN, G6PD, ATP11C, SIRT2, TRIM28, ESR2, TP53, ADRA2A, CETN1, NEDD4, FOXP3, LEF1, SORCS3, ADAP2, VCP, GAS6, DNM3, XCL1, UTS2R, RAPGEF2, BDKRB2, INS, BNIP3, CRH, AVPR2, UCN2, ATP2A1, AHCYL1, CASQ1, IDO1, IGF2, EDA, SPR, SELP, TAS1R3, PAWR, CD63, VDR, PCSK6, RAG2, PDE4A, ITPR1, PDE3B, PDE4D, OPRK1, PDE10A, PDE2A, HDAC2, RINT1, AMPD3, AR, TNFSF11, PHB, TOX3, GNAT1, KHDRBS1, SPI1, XRCC4, HCN4, GNAT3, YWHAE, NUDT9, AGT, CXCL13, PTK2B, ADRB1, LRRC8A, GHRL, C5, CHRNA3, HSP90AB1, SLC6A4, TET1, KCNH2, MTOR, ZFPM1, ZFP42, CAMK2D, ATP1A1, SMO, AQP1, FKBP1B, ATP2B4, HPS4, RYR2, GPER1, XRCC6BP1, NOS1, SOX9, CAMK2G, GNAS, TRIM24, NFX1, RYR1, PDE6A, PGD, PGR, PDE7A, PDE1C, ADORA1, PDE6C, RIPK1, CX3CR1, NR3C1, NT5C1A, SIRT1, UTS2, SIX1, PPP3CB, HOMER1, MED1, EDN1, HELB, CETN2, PIK3CG, FER, ARID1A, LIG4, ADAM8, FGFR2, BCL11B, SREBF1, TCF3, HIBADH, HMGCR, C2CD5, MMP28, WNT10B, GATA3, PIK3R6, ADRA1B, RYR3, RAG1, TNFAIP3, CDC42, CTNNB1, TKT, CD34, CBFA2T3, PARP10, TREM1, BMP5, LAMP2, NCBP2, REST, TAS1R2 |
| ZCP24 | Acoramone | GGCX, NDRG2, METRNL, SLC6A4, WNT11, SNCA, CCM2L, PTGS1, NAMPT, MAPK9, BDKRB2, ALOX15B, CPLX2, PTGS2, RGCC, PTGIS, NEUROD2, CDC20, ALOX15, SERPINB7, FNDC5, HEG1, INS, NAPRT, ANXA1, PDE3A, AVPR1A, AVP, ANAPC2, MECOM, PRDM16 |
| ZCP25 | Isoeleutherol Glucoside | TOP2A, TUBA3C, TOP2B, TUBB, TUBA1C, TUBA3D, TUBB4A, TUBB2A, TUBA1A, TUBB2B, TUBA1B, TUBB4B, TUBA4A |
| ZCP26 | Cis-Methyl Isoeugenol | PDE4A, DHFR, PDE3B, PDE5A, INSR, DPYD, ADK, GAS6, HSP90AB1, ATIC, RAB8B, PARP10, HCN2, PDE3A, PDE10A, DMTN, PDE9A, P2RX2, HOXA5, IL1B, CMPK2, CHRNB2, AK9, HYAL2, CHRNB4, CACNA1C, PDE2A, KCNMA1, TBPL1, HTR2A, FBLN1, NT5M, PDE1B, SLC6A4, CDKN1A, DNMT3B, MGMT, TRDMT1, TYMS, ABCC2, DNMT3A, SIRT2, SLC22A6, P2RX3, DPYS, DUT, HIF1A, HSP90AA1, DTYMK, DHFRL1, SHMT1, FOLR2, STUB1, HCN4, ASNS, AURKA, CHRNA3, RAPGEF2, FOLR1, DNMT1, PDE11A |
| ZCP27 | Asarone | PDE4A, DHFR, PDE3B, PDE5A, INSR, DPYD, ADK, GAS6, HSP90AB1, ATIC, RAB8B, PARP10, HCN2, PDE3A, PDE10A, DMTN, PDE9A, P2RX2, HOXA5, IL1B, CMPK2, CHRNB2, AK9, HYAL2, CHRNB4, CACNA1C, PDE2A, KCNMA1, TBPL1, HTR2A, FBLN1, NT5M, PDE1B, SLC6A4, CDKN1A, DNMT3B, MGMT, TRDMT1, TYMS, ABCC2, DNMT3A, SIRT2, SLC22A6, P2RX3, DPYS, DUT, HIF1A, HSP90AA1, DTYMK, DHFRL1, SHMT1, FOLR2, STUB1, HCN4, ASNS, AURKA, CHRNA3, RAPGEF2, FOLR1, DNMT1, PDE11A |
| HZ1 | Peraksine | KCNJ8, HTR1B, DRD2, KCNJ1, ACE, CACNA1I, HRH1, CHRM2, KCNJ12, KCNJ11, KCNJ10, ADRA2B, CACNA1H, CACNA1S, HTR1F, CHRM4, HTR2A, KCNJ15, HTR1A, KCNJ14, CACNA1F, CACNA1C, DRD5, HTR2B, HTR2C, ADRA2C, SLC18A1, CHRM3, CACNA1D, CACNA1G, ADRA2A, HTR1D, DRD3, SLC18A2, CHRM1, CHRM5, DRD1 |
| HZ2 | Catechol | ALOX5, SERPINE1 |
| HZ3**^*^** | Coriose |  |
| HZ4**^*^** | Linoleic Acid |  |
| HZ5 | Gallicacid | SEC14L3, PPP2CB, AKR1C1, AKR1C2, CHUK, PPP2CA, SEC14L2, PTGS1, PPARG, PRKCA, DGKA, PTGS2, MPO, NR1I2, PRKCB, SEC14L6, DDC, ALOX5, SEC14L4, PLA2G2E, IKBKB |
| HZ6 | Myristic Acid | AKR1D1, ABAT, COX5B, SCN7A, COX5A, COX4I1, SCN4A, CES1, COX2, SUCLG2, SLC25A10, SUCNR1, OXCT1, SDHA, DCT, JMJD6, HSD17B11, P4HB, GRIN3B, CACNA1B, GRIN3A, GRIN1, SIRT1, SIX1, OSBPL8, UBE2B, STIM2, COLGALT1, ARID1A, ACOT4, SLC25A12, CDH11, BDKRB2, SMAD7, WNT10B, STAR, CAMK2D, HTT, TST, TMEM110, EPHA4, EDA, KCNQ1, UGT1A1, PLOD2, SLC25A1, TYR, SCN1A, COX7C, SCN5A, COX3, HDAC9, SCN4B, ACADSB, OGDH, SLC13A2, P3H3, SLC13A3, PLOD3, P4HA2, AKR1C3, SLC13A4, TYRP1, CACNA2D1, CACNA2D2, GRIN2C, SLC7A1, HDAC1, SHMT1, BDH1, SPI1, CYP1A2, PKD2, NEDD4, EGLN2, PHGDH, YBX3, CACNA1D, PRPH, PRDM8, OGFOD1, DLST, AP3D1, IL10, SLC1A6, CDC42, OCA2, HACL1, CASQ2, DLD, CYP1A1, SRD5A2, ESRRG, COX1, SCN10A, AR, PLA2G1B, ADH1C, SCN8A, HDAC2, SLC13A1, OXCT2, ASPH, P4HA1, SUCLA2, CRTAP, SDHAF2, HIF1AN, PLAT, GRIN2A, GRIN2B, SLC7A4, GPD1L, SUGCT, COLGALT2, SUOX, CCL5, ALDH9A1, CYP11A1, ACAT1, PNLIPRP2, 8-Mar, DDC, BCKDHB, UROD, SLC38A7, PF4, MYO5A, EGLN3, NPPA, ARHGEF2, HSD17B8, RYR2, GJA5, GNAS, SULT2A1, SCN11A, SCN3B, SCN3A, ALDH5A1, COX7A1, COX6A2, COX6B1, COX7B, COX8A, SUCLG1, TMLHE, BBOX1, SDHC, SDHD, SLIT2, SLC1A3, UROS, SLC7A2, TOP1, SLC7A3, GRIN2D, SRD5A1, ACSS2, SIX4, CCL2, ANK2, TH, DHODH, GALC, CYP39A1, STIM1, DHTKD1, KCNE5, UGT8, ACSS1, CRACR2A, ILVBL, OGDHL, ACADM, EGLN1, CTNNB1, HAP1, DHRS9, SLC25A13, NFX1, SCN2B, COX6C, AKR1C2, SCN2A, FECH, SCN9A, FABP6, SCN1B, NR1H4, HSD17B6, PLOD1, SDHB, P3H2, P3H1, ACO2, SALL1, SLC13A5, CACNA1A, PLG, KARS, ADORA1, FBXO45, ARV1, MRPS36, BCKDK, HSD17B2, EPO, CLN3, PLCG2, BCKDHA, GAL3ST1, NEDD4L, SRD5A3, ETHE1, CYP4B1, HSD17B1, CAV3, KCNA5, SQRDL, ETFDH, P4HA3, PCSK9, GPR143, SFXN5, DBT |
| HZ7**^*^** | Oleic Acid |  |
| HZ8**^*^** | Catharanthamine |  |
| HZ9 | Lauric Acid | AKR1D1, ABAT, COX5B, SCN7A, COX5A, COX4I1, SCN4A, CES1, COX2, SUCLG2, SLC25A10, SUCNR1, OXCT1, SDHA, DCT, JMJD6, HSD17B11, P4HB, GRIN3B, CACNA1B, GRIN3A, GRIN1, SIRT1, SIX1, OSBPL8, UBE2B, STIM2, COLGALT1, ARID1A, ACOT4, SLC25A12, CDH11, BDKRB2, SMAD7, WNT10B, STAR, CAMK2D, HTT, TST, TMEM110, EPHA4, EDA, KCNQ1, UGT1A1, PLOD2, SLC25A1, TYR, SCN1A, COX7C, SCN5A, COX3, HDAC9, SCN4B, ACADSB, OGDH, SLC13A2, P3H3, SLC13A3, PLOD3, P4HA2, AKR1C3, SLC13A4, TYRP1, CACNA2D1, CACNA2D2, GRIN2C, SLC7A1, HDAC1, SHMT1, BDH1, SPI1, CYP1A2, PKD2, NEDD4, EGLN2, PHGDH, YBX3, CACNA1D, PRPH, PRDM8, OGFOD1, DLST, AP3D1, IL10, SLC1A6, CDC42, OCA2, HACL1, CASQ2, DLD, CYP1A1, SRD5A2, ESRRG, COX1, SCN10A, AR, PLA2G1B, ADH1C, SCN8A, HDAC2, SLC13A1, OXCT2, ASPH, P4HA1, SUCLA2, CRTAP, SDHAF2, HIF1AN, PLAT, GRIN2A, GRIN2B, SLC7A4, SRD5A1, SUGCT, COLGALT2, SUOX, CCL5, ALDH9A1, CYP11A1, ACAT1, PNLIPRP2, 8-Mar, DDC, BCKDHB, UROD, SLC38A7, PF4, MYO5A, EGLN3, NPPA, ARHGEF2, HSD17B8, RYR2, GJA5, GNAS, SULT2A1, SCN11A, SCN3B, SCN3A, ALDH5A1, COX7A1, COX6A2, COX6B1, COX7B, COX8A, SUCLG1, TMLHE, BBOX1, SDHC, SDHD, SLIT2, SLC1A3, UROS, SLC7A2, TOP1, SLC7A3, GRIN2D, FBXO45, ACSS2, SIX4, CCL2, ANK2, TH, DHODH, GALC, CYP39A1, STIM1, DHTKD1, KCNE5, UGT8, ACSS1, CRACR2A, ILVBL, OGDHL, ACADM, EGLN1, CTNNB1, HAP1, DHRS9, SLC25A13, NFX1, GPD1L, SCN2B, COX6C, AKR1C2, SCN2A, FECH, SCN9A, FABP6, SCN1B, NR1H4, HSD17B6, PLOD1, SDHB, P3H2, P3H1, ACO2, SALL1, SLC13A5, CACNA1A, PLG, KARS, ADORA1, ARV1, MRPS36, BCKDK, HSD17B2, EPO, CLN3, PLCG2, BCKDHA, GAL3ST1, NEDD4L, SRD5A3, ETHE1, CYP4B1, HSD17B1, CAV3, KCNA5, SQRDL, ETFDH, P4HA3, PCSK9, GPR143, SFXN5, DBT |
| HZ10**^*^** | Stearic Acid |  |
| HZ11**^*^** | Docosanoic Acid |  |
| HZ12 | Ellipticine | TOP2A, TOP2B, GABRB2, GABRB3, F2, GPR27, CD36, SDC4, CD47, BICD1 |
| HZ13 | Capric Acid | FURIN, SCN11A, SCN3B, SCN3A, ALDH5A1, COX7A1, COX6A2, COX6B1, COX7B, COX8A, SUCLG1, TMLHE, BBOX1, SDHC, SDHD, SLIT2, SLC1A3, UROS, SLC7A2, TOP1, SLC7A3, GRIN2D, CALM2, SRD5A1, ACSS2, SIX4, CCL2, ANK2, TH, DHODH, GALC, CYP39A1, STIM1, DHTKD1, KCNE5, UGT8, ACSS1, CRACR2A, ILVBL, OGDHL, ACADM, EGLN1, CTNNB1, HAP1, DHRS9, SLC25A13, NFX1, GLTP, SCN2B, COX6C, AKR1C2, SCN2A, FECH, SCN9A, FABP6, SCN1B, NR1H4, HSD17B6, PLOD1, SDHB, P3H2, P3H1, ACO2, SALL1, SLC13A5, CACNA1A, PLG, KARS, ADORA1, CALM3, FBXO45, ARV1, MRPS36, BCKDK, HSD17B2, EPO, CLN3, PLCG2, BCKDHA, GAL3ST1, NEDD4L, SRD5A3, ETHE1, CYP4B1, HSD17B1, CAV3, KCNA5, SQRDL, ETFDH, P4HA3, PCSK9, GPR143, SFXN5, DBT, AKR1D1, ABAT, COX5B, SCN7A, COX5A, COX4I1, SCN4A, CES1, COX2, SUCLG2, SLC25A10, SUCNR1, OXCT1, SDHA, DCT, JMJD6, HSD17B11, P4HB, GRIN3B, CACNA1B, GRIN3A, GRIN1, CALM1, SIRT1, SIX1, OSBPL8, UBE2B, STIM2, COLGALT1, ARID1A, ACOT4, SLC25A12, CDH11, BDKRB2, SMAD7, WNT10B, STAR, CAMK2D, HTT, TST, TMEM110, EPHA4, EDA, KCNQ1, UGT1A1, PLOD2, SLC25A1, TYR, SCN1A, COX7C, SCN5A, COX3, HDAC9, SCN4B, ACADSB, OGDH, SLC13A2, P3H3, SLC13A3, PLOD3, P4HA2, AKR1C3, SLC13A4, TYRP1, CACNA2D1, CACNA2D2, GRIN2C, SLC7A1, DRD2, HDAC1, SHMT1, BDH1, SPI1, CYP1A2, PKD2, NEDD4, EGLN2, PHGDH, YBX3, CACNA1D, PRPH, PRDM8, OGFOD1, DLST, AP3D1, IL10, SLC1A6, CDC42, OCA2, HACL1, CASQ2, DLD, CYP1A1, SRD5A2, ESRRG, COX1, SCN10A, AR, PLA2G1B, ADH1C, SCN8A, HDAC2, SLC13A1, OXCT2, ASPH, P4HA1, SUCLA2, CRTAP, SDHAF2, HIF1AN, PLAT, GRIN2A, GRIN2B, SLC7A4, DRD1, GPD1L, SUGCT, COLGALT2, SUOX, CCL5, ALDH9A1, CYP11A1, ACAT1, PNLIPRP2, 8-Mar, DDC, BCKDHB, UROD, SLC38A7, PF4, MYO5A, EGLN3, NPPA, ARHGEF2, HSD17B8, RYR2, GJA5, GNAS, SULT2A1 |
| HZ14 | Cheilanthifoline | CHRM3, HTR3A, DRD2, BCHE, ACHE SLC18A2, CHRM2, CHRNA2 |
| HZ15**^*^** | Arachidic Acid |  |
| HZ16 | Benzoic Acid | GABRA2, GABRA3, GABRQ, TSPO, GABRD, GABRG1, GABRB3, GABRG2, GABRB1, GABRP, GABRA5, ALAD, GABRG3, GABRA4, GABRA6, GABRE, GABRB2, GABRA1 |
| HZ17 | Teresautalic Acid | ESRRG, AKR1C2, COX7A1, TYR, SRD5A2, SCN11A, SCN1A, SCN3A, SCN7A, SLC13A3, SDHB, SCN4B, SCN8A, P4HA2, DCT, JMJD6, HSD17B11, P4HB, CACNA2D2, GRIN2B, HDAC1, SHMT1, BDH1, SPI1, CYP1A2, PKD2, NEDD4, EGLN2, PHGDH, YBX3, CACNA1D, PRPH, PRDM8, OGFOD1, DLST, AP3D1, IL10, SLC1A6, CDC42, OCA2, HACL1, CASQ2, DLD, CYP1A1, COX6C, AKR1D1, FECH, ADH1C, COX7B, SCN2B, SCN3B, SLC25A10, PLOD1, ALDH5A1, HDAC9, PLOD3, SCN1B, SUCLA2, AKR1C3, SLC13A4, TYRP1, CACNA2D1, GRIN2A, GRIN3A, GPD1L, SUGCT, COLGALT2, SUOX, CCL5, ALDH9A1, CYP11A1, ACAT1, PNLIPRP2, 8-Mar, DDC, BCKDHB, UROD, SLC38A7, PF4, MYO5A, EGLN3, NPPA, ARHGEF2, HSD17B8, RYR2, GJA5, GNAS, SULT2A1, COX5B, COX5A, COX4I1, COX6B1, COX2, SUCLG2, SLC13A1, P3H3, SCN5A, SCN2A, SCN9A, P4HA1, P3H2, HDAC2, CRTAP, SDHAF2, HIF1AN, PLAT, PLG, GRIN2D, SRD5A1, ACSS2, SIX4, CCL2, ANK2, TH, DHODH, GALC, CYP39A1, STIM1, DHTKD1, KCNE5, UGT8, ACSS1, CRACR2A, ILVBL, OGDHL, ACADM, EGLN1, CTNNB1, HAP1, DHRS9, SLC25A13, NFX1, COX7C, COX3, PLA2G1B, FABP6, COX8A, SLC13A2, SUCLG1, OXCT2, SCN10A, ASPH, OXCT1, SDHC, SDHA, SDHD, SLIT2, SLC1A3, UROS, CACNA1A, CACNA1B, ADORA1, FBXO45, ARV1, MRPS36, BCKDK, HSD17B2, EPO, CLN3, PLCG2, BCKDHA, GAL3ST1, NEDD4L, SRD5A3, ETHE1, CYP4B1, HSD17B1, CAV3, KCNA5, SQRDL, ETFDH, P4HA3, PCSK9, GPR143, SFXN5, DBT, COX1, AR, COX6A2, CES1, NR1H4, ABAT, HSD17B6, TMLHE, SUCNR1, BBOX1, SCN4A, ACADSB, OGDH, P3H1, ACO2, SALL1, SLC13A5, GRIN3B, GRIN2C, GRIN1, SIRT1, SIX1, OSBPL8, UBE2B, STIM2, COLGALT1, ARID1A, ACOT4, SLC25A12, CDH11, BDKRB2, SMAD7, WNT10B, STAR, CAMK2D, HTT, TST, TMEM110, EPHA4, EDA, KCNQ1, UGT1A1, PLOD2, SLC25A1 |
| HZ18**^*^** | Arjungenin |  |
| MX1 | Artemisia Ketone | PDE7B, CYP17A1, PRKDC, PIK3CA, PIK3CB, ITPR3, ATM, CYP19A1, PDE11A, PIK3R1, ADA, HMGA2, TAC1, FURIN, G6PD, ATP11C, SIRT2, TRIM28, ESR2, TP53, ADRA2A, CETN1, NEDD4, FOXP3, LEF1, SORCS3, ADAP2, VCP, GAS6, DNM3, XCL1, UTS2R, RAPGEF2, BDKRB2, INS, BNIP3, CRH, AVPR2, UCN2, ATP2A1, AHCYL1, CASQ1, IDO1, IGF2, EDA, SPR, SELP, TAS1R3, PAWR, CD63, VDR, PCSK6, RAG2, PDE5A, PDE3A, PIK3CD, ADORA2B, ITPR2, POLA2, PDE6B, PDE8B, PDE8A, WNT4, HAP1, TNFSF11, PHB, TOX3, GNAT1, KHDRBS1, SPI1, XRCC4, HCN4, GNAT3, YWHAE, NUDT9, AGT, CXCL13, PTK2B, ADRB1, LRRC8A, GHRL, C5, CHRNA3, HSP90AB1, SLC6A4, TET1, KCNH2, MTOR, ZFPM1, ZFP42, CAMK2D, ATP1A1, SMO, AQP1, FKBP1B, ATP2B4, HPS4, RYR2, GPER1, XRCC6BP1, NOS1, SOX9, CAMK2G, GNAS, TRIM24, NFX1, PDE9A, ADORA2A, ESR1, PDE1A, PDE1B, PDE4C, PDE4B, NT5E, NR3C2, TACR2, IDNK, NT5C1A, SIRT1, UTS2, SIX1, PPP3CB, HOMER1, MED1, EDN1, HELB, CETN2, PIK3CG, FER, ARID1A, LIG4, ADAM8, FGFR2, BCL11B, SREBF1, TCF3, HIBADH, HMGCR, C2CD5, MMP28, WNT10B, GATA3, PIK3R6, ADRA1B, RYR3, RAG1, TNFAIP3, CDC42, CTNNB1, TKT, CD34, CBFA2T3, PARP10, TREM1, BMP5, LAMP2, NCBP2, REST, TAS1R2, PDE4A, ITPR1, PDE3B, PDE4D, OPRK1, PDE10A, PDE2A, HDAC2, RINT1, AMPD3, HDAC1, ACTN3, DGKI, STUB1, QDPR, SYT2, SHANK3, NKX3-1, CACNA1A, COMT, POLA1, BMP2, SHH, FGF10, ADRBK1, VTI1A, AURKA, BCL2, DRD2, SRC, STAP1, HIPK2, CSF2, HIF1A, SLC18A3, UBR5, PF4, IL10, PPP3R1, RAB8B, TAC4, IL4, PGLS, DRD1, HSP90AA1, CALHM1, HPRT1, TAL1, PDGFB, NEFH, TPM1, CRHR2, RYR1, PDE6A, PGD, PGR, PDE7A, PDE1C, ADORA1, PDE6C, RIPK1, CX3CR1, TFAP2C, NOS1AP, FKBP1A, CTR9, TGFB1, SIX4, TAS1R1, UBE2B, STAT5A, SLC26A6, CRP, TNF, SERPINB3, KCNB1, MAOB, SLC44A4, MCM3, 8-Mar, PLN, LONP1, HRC, PPP3CA, TALDO1, KIT, ABCC4, AREG, ATP1A2, NEFL, SPX, DKK3, DPPA3, POLB, SLC9A3R1, ADAP1, LTA, WNT5A, AMICA1, ALAD, RTN2, GLYR1, PIK3R5, HCN2 |
| MX2 | Elemol | ESR1, TRPM8, TOX3, NEDD4, SRC, WNT5A, PGR, TRPA1, TGFB1, SHH, UBR5, SOX9, WNT4, TFAP2C, MED1, SERPINB3, AREG, PDGFB, TRPV3, TNFSF11, NKX3-1, LEF1, CD34, VDR, OPRK1, PHB, ESR2, FGFR2, GPER1, TRIM24 |
| MX3 | Cynaropicrin | HMGCR, ITGB2, ITGAL, HDAC2 |
| MX4**^*^** | Betulin |  |
| MX5 | (E)-9-Isopropyl-6-Methyl-5,9-Decadiene-2-One | PDE7B, CYP17A1, PRKDC, PIK3CA, PIK3CB, ITPR3, ATM, CYP19A1, PDE11A, PIK3R1, ADA, HMGA2, TAC1, FURIN, G6PD, ATP11C, SIRT2, TRIM28, ESR2, TP53, ADRA2A, CETN1, NEDD4, FOXP3, LEF1, SORCS3, ADAP2, VCP, GAS6, DNM3, XCL1, UTS2R, RAPGEF2, BDKRB2, INS, BNIP3, CRH, AVPR2, UCN2, ATP2A1, AHCYL1, CASQ1, IDO1, IGF2, EDA, SPR, SELP, TAS1R3, PAWR, CD63, VDR, PCSK6, RAG2, PDE5A, PDE3A, PIK3CD, ADORA2B, ITPR2, POLA2, PDE6B, PDE8B, PDE8A, WNT4, HAP1, TNFSF11, PHB, TOX3, GNAT1, KHDRBS1, SPI1, XRCC4, HCN4, GNAT3, YWHAE, NUDT9, AGT, CXCL13, PTK2B, ADRB1, LRRC8A, GHRL, C5, CHRNA3, HSP90AB1, SLC6A4, TET1, KCNH2, MTOR, ZFPM1, ZFP42, CAMK2D, ATP1A1, SMO, AQP1, FKBP1B, ATP2B4, HPS4, RYR2, GPER1, XRCC6BP1, NOS1, SOX9, CAMK2G, GNAS, TRIM24, NFX1, PDE9A, ADORA2A, ESR1, PDE1A, PDE1B, PDE4C, PDE4B, NT5E, NR3C2, TACR2, IDNK, NT5C1A, SIRT1, UTS2, SIX1, PPP3CB, HOMER1, MED1, EDN1, HELB, CETN2, PIK3CG, FER, ARID1A, LIG4, ADAM8, FGFR2, BCL11B, SREBF1, TCF3, HIBADH, HMGCR, C2CD5, MMP28, WNT10B, GATA3, PIK3R6, ADRA1B, RYR3, RAG1, TNFAIP3, CDC42, CTNNB1, TKT, CD34, CBFA2T3, PARP10, TREM1, BMP5, LAMP2, NCBP2, REST, TAS1R2, PDE4A, ITPR1, PDE3B, PDE4D, OPRK1, PDE10A, PDE2A, HDAC2, RINT1, AMPD3, HDAC1, ACTN3, DGKI, STUB1, QDPR, SYT2, SHANK3, NKX3-1, CACNA1A, COMT, POLA1, BMP2, SHH, FGF10, ADRBK1, VTI1A, AURKA, BCL2, DRD2, SRC, STAP1, HIPK2, CSF2, HIF1A, SLC18A3, UBR5, PF4, IL10, PPP3R1, RAB8B, TAC4, IL4, PGLS, DRD1, HSP90AA1, CALHM1, HPRT1, TAL1, PDGFB, NEFH, TPM1, CRHR2, RYR1, PDE6A, PGD, PGR, PDE7A, PDE1C, ADORA1, PDE6C, RIPK1, CX3CR1, TFAP2C, NOS1AP, FKBP1A, CTR9, TGFB1, SIX4, TAS1R1, UBE2B, STAT5A, SLC26A6, CRP, TNF, SERPINB3, KCNB1, MAOB, SLC44A4, MCM3, 8-Mar, PLN, LONP1, HRC, PPP3CA, TALDO1, KIT, ABCC4, AREG, ATP1A2, NEFL, SPX, DKK3, DPPA3, POLB, SLC9A3R1, ADAP1, LTA, WNT5A, AMICA1, ALAD, RTN2, GLYR1, PIK3R5 |
| MX6**^*^** | 20-Hexadecanoylingenol |  |
| MX7**^*^** | Camphene |  |
| MX8**^*^** | P-Cymene |  |
| MX9 | Cotarnine | PDE5A, PDE11A |
| MX10 | Elemicin | PDE4A, DHFR, PDE3B, PDE5A, INSR, DPYD, ADK, GAS6, HSP90AB1, ATIC, RAB8B, PARP10, HCN2, PDE3A, PDE10A, DMTN, PDE9A, P2RX2, HOXA5, IL1B, CMPK2, CHRNB2, AK9, HYAL2, CHRNB4, CACNA1C, PDE2A, KCNMA1, TBPL1, HTR2A, FBLN1, NT5M, PDE1B, SLC6A4, CDKN1A, DNMT3B, MGMT, TRDMT1, TYMS, ABCC2, DNMT3A, SIRT2, SLC22A6, P2RX3, DPYS, DUT, HIF1A, HSP90AA1, DTYMK, DHFRL1, SHMT1, FOLR2, STUB1, HCN4, ASNS, AURKA, CHRNA3, RAPGEF2, FOLR1, DNMT1, PDE11A |
| MX11 | Colartin | AR |
| MX12**^*^** | Gamma-Selinene |  |
| MX13 | Germacra-1(10),4,11(13)-Trien-12-Ol | RDH11, RDH5, ALDH1A1, ALDH1A2, ESR1, RDH10, AR, ASCL1, TSPO, RBP3, ALDH1A3, RLBP1, ALDH3A1, PGR, ALDH8A1, THRA, DLX5, OPN5, RETSAT, RDH12, RDH14, RHO, VDR, SHBG, PAX2, RET, DHRS9, RDH13, DHRS4, RDH8, RS1, CTSH, DHRS2, CBR3, THRB, CBR1, DHRS3, RBP1, LRAT, F12, OPN4, HSD17B7, GATA3, CRYM, CYP1A1 |
| MX14 | Aplotaxene | KCND1, GAMT, CAT, KCNC1, KCND2, KCND3, KCNQ1, PTGS1, ADH7, AQP8, ASPA, APOE, MC4R, SCN5A, KCNIP2, SCGB1A1, CACNA1D, PPARD, ALDH3B1, ALDH3B2, GJA5, KCNA3, KCNC3, KCNB1, ADH1C, ALDH2, GUCY1B3, SLC8A1, PTGS2, NFIB, ARX, NRXN1, SOX15, IL1B, SCN10A, GPX7, OXTR, RAPGEF2, NKX2-1, NPPA, RNASE2, FAS, PRKAB1, KCNA1, ADH1B, KCNB2, KCNA4, GATM, TRPV1, ELOVL4, CYGB, IGF1, EDN1, CNTNAP4, GRIN2A, SPARC, CRLF1, ALDH1B1, DARS, MIP, STX1A, NRXN2, DAB2IP, ADH1A, KCNA2, DLG4, KCNA5, KCNA7, KCNK4, FADS2, FBP1, NRXN3, SHANK3, OXT, RNASE4, SLC17A7, SORCS3, FADD, LEP, ANK3, RYR3, PAXBP1, ADORA1, CYP2E1, KCNA10, TPO, KCNC2, RNASE1, KCNA6, IYD, FADS1, DGKI, LRRC4B, ACY3, MAGI2, RAB3A, CYP11A1, RNASE8, SNTG2, NLGN1, KCNE5, ZPR1, PAX7, ADH4, ACY1 |
| MX15 | Cyclododecanone | SCN11A, SCN3A, ALDH5A1, SCN4A, SCN1B, SCN2B, SCN7A, SCN2A, SCN4B, OGDH, ABAT, SCN5A, HDAC9, SRD5A2, HDAC2, SCN1A, SCN10A, TYR, ACADSB, SCN3B, AKR1D1, SCN9A, SCN8A |
| MX16 | Citrulline | PADI3, DDAH2, PADI6, SLC7A1, TFAP2B, OAZ3, OAT, SLC1A5, ABAT, ACY3, BCAT1, SLC1A4, GLRA3, GOT1, ASPH, GLYATL1, GRIN2C, GPT, SLC6A9, AGXT, ACADSB, IARS, GLRA1, DGAT2, P2RX2, SLC24A2, MTRR, BAK1, PDE4D, FCER2, F2RL1, CAMK2D, NAGS, MYL2, PADI2, PADI4, PADI1, SLC7A4, HLCS, SLC7A2, OAZ2, GOT2, SLC1A1, ASPA, GCAT, ADSSL1, DARS2, GNMT, GPR18, LARS, GLDC, AARS, ASRGL1, LARS2, SLC6A5, BCAT2, LYZ, CYBA, SLC8A1, KLRC4-KLRK1, TH, ROBO1, SLC18A1, KLRK1, SLC6A4, UROS, SNCA, RGS2, NOS2, NOS3, AZIN2, ARG2, MAP1S, SLC25A15, SLC25A2, GPT2, GCSH, IARS2, SLC38A3, VDAC3, GRIN3B, GLRB, CTPS1, NARS2, VDAC2, SHMT2, GLYATL2, RNASE1, PCCB, ACY1, LCMT2, GPM6B, CCL2, CCL5, EDN3, SLC9A1, BCL2L11, SLIT3, AIF1, NPPA, CERS1, EDN2, DDAH1, OTC, ASL, HMOX1, CAD, GATM, OAZ1, BAAT, SLC7A8, GLRA2, VARS, GSS, LCMT1, GLYAT, SLC25A12, TNNC1, PIPOX, ALAS2, SLC32A1, ADSS, GLUL, KYNU, PAICS, NOS1AP, GSK3A, HTR2B, IL1B, POR, ECE1, NDOR1, SLC11A2, BAD, ARRDC3, ASS1, NOS1, SLC7A3, KLF4, ATP2B4, ARG1, CPS1, SHMT1, PPAT, ALAS1, NFS1, ASNS, GRIN2A, AARS2, PHYKPL, DARS, GARS, VDAC1, KARS, SLC36A1, AGXT2, SLC25A13, NARS, RARS, EDN1, ADRA2A, SLIT2, HMBS, PLN, IFNG, MT3, LCN2, RYR2 |
| MX17**^*^** | Stigmasterol |  |
| MX18 | Costunolide | CHRM3, AR, CHRM5, CHRM4, CHRM1, NR3C2, GNA15, CHRM2, ARFGEF2, P2RX1, ESR1, DOCK5, DOCK4, PGR, NTSR1, MAP2K1 |
| MX19 | Germacra-1(10),4,11(13)-Trien-12-Al | ACHE, ESR1, NRG1, SLC44A4, CDH8, GRIN1, BCHE, PGR, DMGDH, ALDH7A1, FNTA, COLQ, OPRK1, SIX3, DNM3, AGRN, CYP17A1, NR3C2, CRP, ASCL1, CHKA, F12, SLC5A7, PRSS12, ENPP6, CHDH |
| MX20 | Isoalantolactone | CHRM3, NR3C2, GNA15, CHRM1, ARFGEF2, P2RX1, CHRM2, DOCK5, DOCK4, PGR, NTSR1, MAP2K1, AR, CHRM5, CHRM4 |
| MX21**^*^** | 6,6'-Dimethoxygossypol |  |
| MX22 | Costuslactone | AR, PGR, NR3C2 |
| MX23**^*^** | Taraxasterol |  |
| MX24 | Dehydrocostuslactone | CHRM3, NR3C2, GNA15, CHRM1, ARFGEF2, P2RX1, CHRM2, DOCK5, DOCK4, PGR, NTSR1, MAP2K1, AR, CHRM5, CHRM4 |
| MX25 | Campherenol | VDR, ESR1, GPBAR1, CALB1, TCF3, B4GALT1, RXRA, CYP27B1, PGR, SNAI2, FGF23, BAX, S100G, NR1H4, GC, NFKB1, MED1, GFI1, KL, CYP27A1, TRIM24, SNW1, AKR1C3, SNAI1, LANCL2, CYP2R1, KANK2, F12, CYP24A1, CYP3A4, WNT4, PML, IRX5 |
| MX26**^*^** | Beta-Phellandrene |  |
| MX27 | Gamma-Aminobutyric Acid | CACNA2D1, GRIN2A, GRIN3A, RIPK1, SLC25A20, LCMT1, CPT2, ACADSB, IARS, SLC1A5, SHMT1, SLC7A8, ALAS1, SLC1A4, GLRA3, GLYAT, SLC25A12, NARS2, VDAC2, GPT, SLC6A9, OAZ1, ADSS, CAD, GABBR1, LYZ, CDK5R2, UTS2, EPCAM, FASLG, CETN1, CYP11A1, SLC44A4, DNM3, UTS2R, UROD, PHOX2B, ADCY3, LRRK2, KDM6B, PCSK6, PLAT, PLG, GRIN2D, ASCL1, IARS2, THNSL1, LARS, CPT1A, SLC22A5, GOT2, ABAT, PPAT, GCAT, ADSSL1, DARS2, AARS2, PHYKPL, TNNC1, OAZ2, AARS, SLC7A3, KARS, SLC7A1, OTC, ACY1, PAICS, PDE5A, KHDRBS1, F12, COMT, TH, KCNB1, GHRL, CHRNA3, HMGCR, INS, STAR, CDH8, TACR2, F11, GRM7, CACNA1A, CACNA1B, ADORA1, EPHX2, BCAT1, MPO, CES1, PCCB, CRAT, GPT2, SLC1A1, ACY3, SLC38A3, VDAC3, GOT1, ASPH, GLYATL1, DARS, PIPOX, SHMT2, ASRGL1, AGXT, SLC36A1, GLUL, KYNU, NARS, RGCC, ARPIN, ADORA2A, ADRA2A, PIK3CG, ADRBK1, TBR1, MAD2L2, ACHE, SLC18A3, ATP1A2, RYR3, DRD3, CKB, FOS, GRIN3B, GRIN2C, GRIN1, ADA, VARS, SLC25A29, TARS2, XDH, BCAT2, BAAT, SLC7A2, ASPA, NFS1, GSS, GNMT, GPR18, GATM, OAT, SLC25A2, ALAS2, GLYATL2, RNASE1, SLC6A5, AGXT2, SLC25A13, ABCC2, DGKI, PTEN, ARCN1, CETN2, KCNA1, PRSS12, CEND1, CDK5R1, ANK3, DRD4, ATP13A2, SPX, RERE, PARK2, NLGN3, CACNA2D2, GRIN2B, DLL1, HAP1, TARS, SLC22A4, LARS2, CROT, LCMT2, OAZ3, GCSH, GLRA2, SLC25A15, ASNS, GLRB, CTPS1, ARG1, GLDC, GARS, VDAC1, SLC32A1, ASS1, SLC7A4, ARG2, GLRA1, FBP1, FURIN, SHANK3, EDN1, CRP, KLKB1, SORCS3, DRD2, CHRNB2, COLQ, CRH, AVPR2, ATP2A1, DRD1, NCBP2 |
| MX28 | Dehydrocostus Lactone | CHRM3, NR3C2, GNA15, CHRM1, ARFGEF2, P2RX1, CHRM2, DOCK5, DOCK4, PGR, NTSR1, MAP2K1, AR, CHRM5, CHRM4 |
| MX29**^*^** | Beta-Humulene |  |
| MX30 | Beta-Ionone | PDE7B, CYP17A1, PRKDC, PIK3CA, PIK3CB, ITPR3, ATM, CYP19A1, PDE11A, PIK3R1, ADA, TFAP2C, NOS1AP, FKBP1A, CTR9, TGFB1, SIX4, TAS1R1, UBE2B, STAT5A, SLC26A6, CRP, TNF, SERPINB3, KCNB1, MAOB, SLC44A4, MCM3, 8-Mar, PLN, LONP1, HRC, PPP3CA, TALDO1, KIT, ABCC4, AREG, ATP1A2, NEFL, SPX, DKK3, DPPA3, POLB, SLC9A3R1, ADAP1, LTA, WNT5A, AMICA1, ALAD, RTN2, GLYR1, PIK3R5, HCN2, PDE5A, PDE3A, PIK3CD, ADORA2B, ITPR2, POLA2, PDE6B, PDE8B, PDE8A, WNT4, HAP1, HMGA2, TAC1, FURIN, G6PD, ATP11C, SIRT2, TRIM28, ESR2, TP53, ADRA2A, CETN1, NEDD4, FOXP3, LEF1, SORCS3, ADAP2, VCP, GAS6, DNM3, XCL1, UTS2R, RAPGEF2, BDKRB2, INS, BNIP3, CRH, AVPR2, UCN2, ATP2A1, AHCYL1, CASQ1, IDO1, IGF2, EDA, SPR, SELP, TAS1R3, PAWR, CD63, VDR, PCSK6, RAG2, PDE9A, ADORA2A, ESR1, PDE1A, PDE1B, PDE4C, PDE4B, NT5E, NR3C2, TACR2, IDNK, TNFSF11, PHB, TOX3, GNAT1, KHDRBS1, SPI1, XRCC4, HCN4, GNAT3, YWHAE, NUDT9, AGT, CXCL13, PTK2B, ADRB1, LRRC8A, GHRL, C5, CHRNA3, HSP90AB1, SLC6A4, TET1, KCNH2, MTOR, ZFPM1, ZFP42, CAMK2D, ATP1A1, SMO, AQP1, FKBP1B, ATP2B4, HPS4, RYR2, GPER1, XRCC6BP1, NOS1, SOX9, CAMK2G, GNAS, TRIM24, NFX1, PDE4A, ITPR1, PDE3B, PDE4D, OPRK1, PDE10A, PDE2A, HDAC2, RINT1, AMPD3, AR, NT5C1A, SIRT1, UTS2, SIX1, PPP3CB, HOMER1, MED1, EDN1, HELB, CETN2, PIK3CG, FER, ARID1A, LIG4, ADAM8, FGFR2, BCL11B, SREBF1, TCF3, HIBADH, HMGCR, C2CD5, MMP28, WNT10B, GATA3, PIK3R6, ADRA1B, RYR3, RAG1, TNFAIP3, CDC42, CTNNB1, TKT, CD34, CBFA2T3, PARP10, TREM1, BMP5, LAMP2, NCBP2, REST, TAS1R2, RYR1, PDE6A, PGD, PGR, PDE7A, PDE1C, ADORA1, PDE6C, RIPK1, CX3CR1, HDAC1, ACTN3, DGKI, STUB1, QDPR, SYT2, SHANK3, NKX3-1, CACNA1A, COMT, POLA1, BMP2, SHH, FGF10, ADRBK1, VTI1A, AURKA, BCL2, DRD2, SRC, STAP1, HIPK2, CSF2, HIF1A, SLC18A3, UBR5, PF4, IL10, PPP3R1, RAB8B, TAC4, IL4, PGLS, DRD1, HSP90AA1, CALHM1, HPRT1, TAL1, PDGFB, NEFH, TPM1, CRHR2 |
| MX31 | 5'-Methoxy Dehydrodiisoeugenol | CNR2, PRKCA, DGKA, ABHD6, CHRNB2, DAGLA, CNR1, NR1I2, PRKCB, MGLL, FCER1A, PLIN5, DRD2, ALOX5, SEC14L4, FCER1G, CAV3, ZP3, SEC14L3, PPP2CB, AKT1, SUMO1, RNF207, PPP2CA, SEC14L2, CCR7, GPR55, C3 |
| MX32 | 3-Epizaluzanin C | HMGCR, HDAC1, SPI1, ARID1A, PF4, EDA, ITGB2, LTK, UBE2B, 8-Mar, CAMK2D, ADORA1, ITGAL, SIRT1, TP53, INS, IL10, CLEC4M, HDAC2, SIX1, CAMK2A, WNT10B, CDC42, GNAS, NR3C2, SIX4, CRP, TIGAR, CTNNB1, NFX1 |
| MX33**^*^** | Gamma-Sitosterol |  |
| MX34**^*^** | Beta-Amyrin Palmitate |  |
| MX35 | Alantolactone | CHRM3, AR, CYP19A1, NTSR1, MAP2K1, CHRM1, NR3C1, HDAC2, CHRM5, CHRM4, CHRM2, HMGCR, NR3C2, GNA15, ESR1, ITGB2, ARFGEF2, P2RX1, PGR, ITGAL, DOCK5, DOCK4 |
| MX36 | Arbusculin A | AR |
| MX37 | Saussurea Lactone | CHRM3, NR3C2, GNA15, CHRM1, ARFGEF2, P2RX1, CHRM2, DOCK5, DOCK4, PGR, NTSR1, MAP2K1, AR, CHRM5, CHRM4 |
| MX38**^*^** | Myricadiol |  |
| MX39**^*^** | 22,23-Dihydrostigmasterol |  |
| MX40 | Isodehydrocostus Lactone | CHRM3, HMGCR, ARFGEF2, P2RX1, CHRM1, ITGB2, DOCK5, DOCK4, CHRM2, ITGAL, NTSR1, MAP2K1, PGR, HDAC2, CHRM5, CHRM4, AR, NR3C2, GNA15 |
| MX41 | Santamarin | HMGCR, PGR, LTK, UBE2B, 8-Mar, CAMK2D, ADORA1, ITGB2, AR, SIRT1, TP53, INS, IL10, CLEC4M, ITGAL, NR3C1, SIX1, CAMK2A, WNT10B, CDC42, GNAS, HDAC2, NR3C2, SIX4, CRP, TIGAR, CTNNB1, NFX1, ESR1, HDAC1, SPI1, ARID1A, PF4, EDA |
| MX42 | Linalool | ESR1, PHB, ESR2, FGFR2, GPER1, TRIM24, PGR, TOX3, NEDD4, SRC, WNT5A, WNT4, TGFB1, SHH, UBR5, SOX9, TFAP2C, MED1, SERPINB3, AREG, PDGFB, TNFSF11, NKX3-1, LEF1, CD34, VDR |
| MX43 | 5-Methoxy-N,N-Dimethyl-Tryptamine Nb-Oxide | CALR, ASMT, EPX, NQO2, MPO, PDE3A, MTNR1A, CALM2, ESR1, CALM3, MTNR1B, CALM1 |
| MX44 | Costol | RDH11, RDH5, ALDH1A1, ALDH1A2, OPRK1, RDH10, AR, ASCL1, TSPO, RBP3, ALDH1A3, RLBP1, ALDH3A1, TRPM8, ALDH8A1, THRA, DLX5, OPN5, RETSAT, RDH12, RDH14, RHO, TRPA1, SHBG, PAX2, RET, DHRS9, RDH13, DHRS4, RDH8, RS1, CTSH, DHRS2, CBR3, THRB, CBR1, DHRS3, RBP1, LRAT, TRPV3, OPN4, HSD17B7, GATA3, CRYM, CYP1A1 |
| MX45 | Reynosin | HMGCR, LTK, UBE2B, 8-Mar, CAMK2D, ADORA1, ITGB2, SIRT1, TP53, INS, IL10, CLEC4M, ITGAL, SIX1, CAMK2A, WNT10B, CDC42, GNAS, HDAC2, SIX4, CRP, TIGAR, CTNNB1, NFX1, HDAC1, SPI1, ARID1A, PF4, EDA |
| MX46**^*^** | Myrcene |  |
| MX47**^*^** | Dehydro-Alpha-Curcumene |  |
| MX48**^*^** | Isoallylbenzene |  |
| MX49 | Alpha-Cyclocostunolide | CHRM3, PGR, PTGER2, NR3C2, GNA15, CHRM1, AR, PTGER3, ARFGEF2, P2RX1, CHRM2, NR3C1, ITGAL, DOCK5, DOCK4, ESR1, HMGCR, CYP19A1, NTSR1, MAP2K1, PTGER4, ITGB2, HDAC2, CHRM5, CHRM4 |
| MX50 | Zaluzanin C | HMGCR, HDAC1, SPI1, ARID1A, PF4, EDA, ITGB2, LTK, UBE2B, 8-Mar, CAMK2D, ADORA1, ITGAL, SIRT1, TP53, INS, IL10, CLEC4M, HDAC2, SIX1, CAMK2A, WNT10B, CDC42, GNAS, NR3C2, SIX4, CRP, TIGAR, CTNNB1, NFX1 |
| SX1 | Androst-4-Ene-3,17-Dione | CYP17A1, AR, RYR1, PRKDC, ADORA2B, PDE1B, PDE10A, PDE2A, HDAC2, RIPK1, CX3CR1, PRLR, HMGA2, TAC1, FURIN, G6PD, ATP11C, SIRT2, TRIM28, ESR2, TP53, ADRA2A, CETN1, NEDD4, FOXP3, LEF1, SORCS3, ADAP2, VCP, GAS6, DNM3, XCL1, UTS2R, RAPGEF2, BDKRB2, INS, BNIP3, CRH, AVPR2, UCN2, ATP2A1, AHCYL1, CASQ1, IDO1, IGF2, EDA, SPR, SELP, TAS1R3, PAWR, CD63, VDR, PCSK6, RAG2, ESR1, PDE7B, PDE3A, PIK3CD, PDE1A, PDE7A, PDE1C, ADORA1, PDE6C, PIK3R1, ADA, NR3C1, TNFSF11, PHB, TOX3, GNAT1, KHDRBS1, SPI1, XRCC4, HCN4, GNAT3, YWHAE, NUDT9, AGT, CXCL13, PTK2B, ADRB1, LRRC8A, GHRL, C5, CHRNA3, HSP90AB1, SLC6A4, TET1, KCNH2, MTOR, ZFPM1, ZFP42, CAMK2D, ATP1A1, SMO, AQP1, FKBP1B, ATP2B4, HPS4, RYR2, GPER1, XRCC6BP1, NOS1, SOX9, CAMK2G, GNAS, TRIM24, NFX1, PGR, PDE5A, ADORA2A, PDE3B, PDE4D, ITPR3, ATM, CYP19A1, PDE11A, WNT4, HAP1, ANXA1, NT5C1A, SIRT1, UTS2, SIX1, PPP3CB, HOMER1, MED1, EDN1, HELB, CETN2, PIK3CG, FER, ARID1A, LIG4, ADAM8, FGFR2, BCL11B, SREBF1, TCF3, HIBADH, HMGCR, C2CD5, MMP28, WNT10B, GATA3, PIK3R6, ADRA1B, RYR3, RAG1, TNFAIP3, CDC42, CTNNB1, TKT, CD34, CBFA2T3, PARP10, TREM1, BMP5, LAMP2, NCBP2, REST, TAS1R2, OPRK1, PDE9A, ITPR1, PGD, PIK3CB, POLA2, PDE6B, PDE8B, PDE8A, TACR2, IDNK, HDAC1, ACTN3, DGKI, STUB1, QDPR, SYT2, SHANK3, NKX3-1, CACNA1A, COMT, POLA1, BMP2, SHH, FGF10, ADRBK1, VTI1A, AURKA, BCL2, DRD2, SRC, STAP1, HIPK2, CSF2, HIF1A, SLC18A3, UBR5, PF4, IL10, PPP3R1, RAB8B, TAC4, IL4, PGLS, DRD1, HSP90AA1, CALHM1, HPRT1, TAL1, PDGFB, NEFH, TPM1, CRHR2, NR3C2, PDE4A, PDE6A, PIK3CA, ITPR2, PDE4C, PDE4B, NT5E, RINT1, AMPD3, SRD5A1, TFAP2C, NOS1AP, FKBP1A, CTR9, TGFB1, SIX4, TAS1R1, UBE2B, STAT5A, SLC26A6, CRP, TNF, SERPINB3, KCNB1, MAOB, SLC44A4, MCM3, 8-Mar, PLN, LONP1, HRC, PPP3CA, TALDO1, KIT, ABCC4, AREG, ATP1A2, NEFL, SPX, DKK3, DPPA3, POLB, SLC9A3R1, ADAP1, LTA, WNT5A, AMICA1, ALAD, RTN2, GLYR1, PIK3R5, HCN2 |
| SX2 | Testosterone | AR, NR3C1, NOTCH2, FADS2, HSD11B1, PAK6, NFKB1, SMAD4, CDK6, PTEN, NCOR1, GSK3A, DAXX, PRKDC, KDM1A, FHL2, STAT3, PELP1, ADAM10, AHR, POU2F2, UBE2I, HDAC3, NR2C2, RAC3, PRPF6, PARK7, HSPA1A, MAGEA11, PSPC1, PMEPA1, HSD17B1, PRDX1, KAT2B, RNF6, CDK7, TMF1, PIAS3, HDAC4, PIAS4, ERG, SPDEF, PPIA, CREBRF, WNT4, FXYD2, AKAP13, SRD5A1, OPRK1, SLC8A1, FADS1, CREBBP, CASP8, HDAC1, COX5B, HMGB2, CASP3, RAF1, CDC37, PSMC3IP, WHSC1, SMAD3, PNRC2, SELENBP1, GTF2F1, PA2G4, PRMT1, AES, GRIP1, SRY, HMGB1, NCOA6, NRIP1, DDC, ACTB, CCNE1, GNB2L1, NISCH, TRIM68, POLR2A, BAG1, RAN, CTNNB1, CDK9, MED14, SMARCA4, GTF2H1, CALM1, DAP3, ATP1A2, NKX3-1, TGFB2, TSPO, RAD51, CYP17A1, ANXA1, F12, PTGS1, RELA, RANBP9, STUB1, CALR, BRCA1, EP300, MED1, KLK3, CEBPA, CDC25A, CCNH, PARP1, CCND1, RUNX1, SVIL, ATF2, PATZ1, PXN, IL6ST, JUN, SRC, UXT, KLK2, TRIP4, PNRC1, RB1, CAV1, ETV5, TGIF1, HIPK3, NONO, EFCAB6, HSP90AA1, CASP1, IARS, EGFR, NCOA4, CAMLG, PPIF, BMP2, DKK3, BMP5, REST, ESR1, NR3C2, PRLR, PTGS2, AKT1, PIAS1, GAPDH, XRCC5, TCF4, NSD1, GAK, SART3, KAT7, FOXA1, SUMO1, TGFB1I1, TDG, APPBP2, APOL2, CDK1, GSK3B, JMJD1C, GTF2F2, HSPB2, RNF4, UBE3A, SP1, YWHAQ, HIF1A, MDM2, SMAD1, RBAK, NCOA2, PIAS2, NR2C1, XRCC6, RNF14, FOXO1, DCAF6, KIF1A, TRIM24, ATP1A3, COL5A1, SHBG, NR1I3, PDE8B, PGR, ATP1A1, TRPV1, ELOVL4, RUVBL1, NCOR2, NR0B2, MAPK1, CDC25B, FLNA, POU5F1, MYBBP1A, TP53, WIPI1, NR0B1, IFI16, TBP, UBC, TOP1, TSG101, RUNX2, CASP7, SMARCC1, CUL4B, RCHY1, CTDSP2, NCOA3, SIN3A, POU2F1, RAD9A, IDE, NCOA1, DCC, ZMIZ1, NR5A1, GSN, CEBPB, KAT5, RAD54L2, RNASEL, SLC25A4, PPP3R2, NOX1, ATP1B1, ALDH1A1, S100A9 |
| SX3 | 3,5-Dihydroxybenzoic Acid | PLA2G2E, PTGS1, APOA2, SERPINB7, NDRG2, OSBPL8, MAPK9, TNF, S100A8, BDKRB2, WNT11, ROCK1, MECOM, PPARA, S100A9, AKR1C2, PTGS2, HSD17B2, AKR1C3, NAMPT, AVPR1A, NCKAP1L, PTGIS, ALOX5AP, NR1H2, ALOX15B, COL1A1, ABHD5, ALOX15, FABP3, PPARG, ALOX5, SERPINE1, IFNG, RGCC, IKBKE, STX3, HEG1, AVP, NEUROD2, DHDH, NR1D1, SNCA, ANXA1, ABCG1, MPO, IKBKB, IL1B, AKR1C4, FNDC5, ITGB3, OXER1, ITGAV, SLC6A4, INS, CDC20, ABCA1, ADIPOQ, PAWR, PRDM16, AKR1C1, CHUK, IL13, TPMT, NR1H3, METRNL, PNPLA2, PTPN2, ROCK2, ANAPC2, DNAJA3, NAPRT, CPLX2, AKR1B10, CCM2L |
| SX4**^*^** | Musennin |  |
| SX5 | 17-Beta-Estradiol | ESR1, ESRRG, NR3C2, PTGER1, PTGER2, PTGER3, OPRM1, TOX3, GPER1, SOX9, TRIM24, SMAD2, FHL2, PPP5C, RXRB, GADD45G, DDX5, SP1, CSNK2B, TBP, CHD9, YWHAQ, PIAS1, NR2C1, NOS3, NR2F6, STUB1, CTNNB1, SMAD4, STRN, PIK3R3, SMARCA2, MAPK11, SDF4, CDC25B, CEBPB, MAPK3, MED14, KMT2D, JUND, JUNB, GRIP1, LCK, MTA3, MAD2L1, GTF2H1, AKAP13, MAPK14, KIF1A, MTA2, OXT, TRIM59, MGMT, CALM1, MED21, KDM5A, PRKACA, CUL4B, AKT2, SRC, PRPF6, GRB2, CALR, SLC12A5, NR1I2, CYP17A1, SLC6A3, DRD1, PTGER4, DRD3, OPRD1, SHH, WNT5A, NKX3-1, ABCB4, MTCH2, MED17, TUBB, AKT1, EIF3I, PPARGC1A, PRMT2, CCND1, XBP1, TRIP4, PTGES3, HEXIM1, PSMB9, MNAT1, CCNE1, TAF10, PNRC1, PPP1CC, PPID, POU2F1, PRMT1, POU1F1, MDM2, RNF14, CAV1, ATAD2, MED24, PIK3R1, POU4F1, KAT5, NCOR1, CCNC, SMARCA4, MAP3K1, PAK1, NCOA1, PSMC5, PSMC3IP, PDLIM1, STAT5A, TP53, ARHGEF15, ZNF398, NCOA4, JUN, BCAS2, WIPI1, CDK2, FOXO3, NRIP1, SLC12A1, SLC12A2, NQO2, ESR2, PGR, DRD5, DRD4, HTR1A, DRD2, ALOX5, LEF1, MED1, VDR, NR1I3, UBE3A, RELA, BAG1, PAGR1, PIAS2, PTPN6, NFATC4, TDG, FHL1, ITGB3BP, FOXO4, FKBP4, NCOR2, SMARCD1, ISL1, CDK7, LDB1, CRIPAK, MED23, MAPK1, SMARCD3, BRCA1, HSP90AA1, SHC1, NCOA7, TCF20, MAPK7, RFX4, FOXO1, UBE2I, HSD17B1, ATP6AP1, CDK8, PPARGC1B, NR2F1, RPS6KA1, MKNK2, TSC2, TRIM25, THRB, SLC30A9, TRRAP, THRA, BAZ1B, BDNF, NUCB2, RBFOX2, NCOA6, RNF4, DAP3, CYP11A1, SLC12A7, MTNR1A, ANXA1, AR, ESRRB, HTR7, CYP19A1, SLC6A2, TFAP2C, TGFB1, FGFR2, WNT4, CREBBP, MED12, HSP90AB1, RBBP5, POLR1B, SRD5A1, FKBP5, MPG, DUT, TNFRSF14, ERBB2, BCAR1, CHUK, NFKB1, RARA, USF1, UBC, NR0B2, GNAI1, SVIL, AHR, DDX54, MED13, TAP1, PRDM2, POU2F2, ZBTB16, EP300, NSD1, MED7, SMARCE1, TRIM28, ASH2L, GADD45A, RXRA, REXO4, HSPA4, EGFR, CEBPA, MTA1, PHB2, GTF2B, BTF3, NPPA, NR2C2, RPS6KA3, TADA3, SMAD3, CCNH, NCOA2, CUEDC2, MTNR1B, MPO, SLC12A4, NR3C1, OPRK1, SLC6A4, NOS2, DBH, NR0B1, SLC2A1, AREG, ABCG2, ABCB1, TUBA1B, MVP, POLR2D, PNRC2, PAK6, VAV3, STAT3, NCOA3, ARNT, SP3, PELP1, RBM39, MMS19, ZBTB17, COPS5, HNF4A, CDKN1A, MED20, ESRRA, PTMA, CALM2, NELFB, IGF1R, MED6, PTEN, GADD45B, PIAS3, RBM23, YWHAH, PTPN1, CCNT1, SOS1, CALM3, DDX17, TRAM1, POU4F2, CITED1, RLIM, FLII, WDR5, DNTTIP2, NR1H4, SREBF1, SAFB, MED10, SAFB2, RXRG, RGS3, MED16, FGR, ACTN2, ASMT, SLC12A6, EPX |
| SX6**^*^** | Allantoin |  |
| SX7 | 3-Methylcyclotridecan-1-One | SCN11A, SCN3A, ALDH5A1, SCN4A, SCN1B, SCN2B, SCN7A, SCN2A, SCN4B, OGDH, ABAT, SCN5A, HDAC9, SRD5A2, HDAC2, SCN1A, SCN10A, TYR, ACADSB, SCN3B, AKR1D1, SCN9A, SCN8A |
| SX8 | Morin | CRYZ, VKORC1, NQO1 |
| SX9 | Muscopyridine | GABRB2, SDC4, GABRB3, CD47, F2, BICD1, GPR27, CD36 |
| SX10 | 5-Cis-Cyclopentadecen-1-One | PDE7B, CYP17A1, PRKDC, PIK3CA, PIK3CB, ITPR3, ATM, CYP19A1, PDE11A, PIK3R1, ADA, TRPV1, PTGS2, TNFSF11, PHB, TOX3, GNAT1, KHDRBS1, SPI1, XRCC4, HCN4, GNAT3, YWHAE, NUDT9, AGT, CXCL13, PTK2B, ADRB1, LRRC8A, GHRL, C5, CHRNA3, HSP90AB1, SLC6A4, TET1, KCNH2, MTOR, ZFPM1, ZFP42, CAMK2D, ATP1A1, SMO, AQP1, FKBP1B, ATP2B4, HPS4, RYR2, GPER1, XRCC6BP1, NOS1, SOX9, CAMK2G, GNAS, TRIM24, NFX1, PDE5A, PDE3A, PIK3CD, ADORA2B, ITPR2, POLA2, PDE6B, PDE8B, PDE8A, WNT4, HAP1, AR, ELOVL4, NT5C1A, SIRT1, UTS2, SIX1, PPP3CB, HOMER1, MED1, EDN1, HELB, CETN2, PIK3CG, FER, ARID1A, LIG4, ADAM8, FGFR2, BCL11B, SREBF1, TCF3, HIBADH, HMGCR, C2CD5, MMP28, WNT10B, GATA3, PIK3R6, ADRA1B, RYR3, RAG1, TNFAIP3, CDC42, CTNNB1, TKT, CD34, CBFA2T3, PARP10, TREM1, BMP5, LAMP2, NCBP2, REST, TAS1R2, PDE9A, ADORA2A, ESR1, PDE1A, PDE1B, PDE4C, PDE4B, NT5E, NR3C2, TACR2, IDNK, FADS2, HDAC1, ACTN3, DGKI, STUB1, QDPR, SYT2, SHANK3, NKX3-1, CACNA1A, COMT, POLA1, BMP2, SHH, FGF10, ADRBK1, VTI1A, AURKA, BCL2, DRD2, SRC, STAP1, HIPK2, CSF2, HIF1A, SLC18A3, UBR5, PF4, IL10, PPP3R1, RAB8B, TAC4, IL4, PGLS, DRD1, HSP90AA1, CALHM1, HPRT1, TAL1, PDGFB, NEFH, TPM1, CRHR2, PDE4A, ITPR1, PDE3B, PDE4D, OPRK1, PDE10A, PDE2A, HDAC2, RINT1, AMPD3, SLC8A1, FADS1, TFAP2C, NOS1AP, FKBP1A, CTR9, TGFB1, SIX4, TAS1R1, UBE2B, STAT5A, SLC26A6, CRP, TNF, SERPINB3, KCNB1, MAOB, SLC44A4, MCM3, PLN, LONP1, HRC, PPP3CA, TALDO1, KIT, ABCC4, AREG, ATP1A2, NEFL, SPX, DKK3, DPPA3, POLB, SLC9A3R1, ADAP1, LTA, WNT5A, AMICA1, ALAD, RTN2, GLYR1, PIK3R5, HCN2, RYR1, PDE6A, PGD, PGR, PDE7A, PDE1C, ADORA1, PDE6C, RIPK1, CX3CR1, F12, PTGS1, HMGA2, TAC1, FURIN, G6PD, ATP11C, SIRT2, TRIM28, ESR2, TP53, ADRA2A, CETN1, NEDD4, FOXP3, LEF1, SORCS3, ADAP2, VCP, GAS6, DNM3, XCL1, UTS2R, RAPGEF2, BDKRB2, INS, BNIP3, CRH, AVPR2, UCN2, ATP2A1, AHCYL1, CASQ1, IDO1, IGF2, EDA, SPR, SELP, TAS1R3, PAWR, CD63, VDR, PCSK6, RAG2 |
| SX11**^*^** | Decamine |  |
| SX12 | Musclide A1 | PROS1, PROC |
| SX13 | Estragole | PDE4A, DHFR, PDE3B, PDE5A, INSR, DPYD, ADK, GAS6, HSP90AB1, ATIC, RAB8B, PARP10, HCN2, PDE3A, PDE10A, DMTN, PDE9A, P2RX2, HOXA5, IL1B, CMPK2, CHRNB2, AK9, HYAL2, CHRNB4, CACNA1C, PDE2A, KCNMA1, TBPL1, HTR2A, FBLN1, NT5M, PDE1B, SLC6A4, CDKN1A, DNMT3B, MGMT, TRDMT1, TYMS, ABCC2, DNMT3A, SIRT2, SLC22A6, P2RX3, DPYS, DUT, HIF1A, HSP90AA1, DTYMK, DHFRL1, SHMT1, FOLR2, STUB1, HCN4, ASNS, AURKA, CHRNA3, RAPGEF2, FOLR1, DNMT1, PDE11A |
| SX14 | Cyclotetradecan-1-One | SCN11A, SCN3A, ALDH5A1, SCN4A, SCN1B, SCN2B, SCN7A, SCN2A, SCN4B, OGDH, ABAT, SCN5A, HDAC9, SRD5A2, HDAC2, SCN1A, SCN10A, TYR, ACADSB, SCN3B, AKR1D1, SCN9A, SCN8A |
| SX15**^*^** | 3beta-Hydroxy-5alpha-Androstan-17-One |  |
| SX16 | Alpha-Estradiol | ESR2, OPRM1, TGFB1, FGFR2, ABCB4, ABCB1, ESR1, ALOX5, MED1, PGR, GPER1, TRIM24, NR1I2, TFAP2C, NKX3-1, ABCG2, WNT5A, OPRD1, SLC2A1, SHH, WNT4, SOX9, OPRK1, TOX3, LEF1, AREG, VDR |
| SX17**^*^** | Androsterone |  |
| SX18**^*^** | Cyclovirobuxine |  |
| SX19**^*^** | Cholesterol |  |
| SX20 | Muscone | SCN11A, SCN3A, ALDH5A1, SCN4A, SCN1B, SCN2B, SCN7A, SCN2A, SCN4B, OGDH, ABAT, SCN5A, HDAC9, SRD5A2, HDAC2, SCN1A, SCN10A, TYR, ACADSB, SCN3B, AKR1D1, SCN9A, SCN8A |
| SX21 | 2,6-Decamethylene Pyridine | GABRB2, SDC4, GABRB3, F2, GPR27, CD36, CD47, BICD1 |
| SX22**^*^** | 3alpha-Hydroxy-5alpha-Androstan-17-One |  |
| SX23 | Muscol | TRPV3, SCN11A, SCN3A, ALDH5A1, SCN4A, SCN1B, NGFR, HIF1A, SMO, CBFA2T3, OPRK1, SCN2B, SCN7A, SCN2A, SCN4B, OGDH, ADRA2A, BNIP3, TACR2, NEFH, TRPM8, ABAT, SCN5A, HDAC9, SRD5A2, HDAC2, TRPV1, CRH, AQP1, CRHR2, TRPA1, SCN1A, SCN10A, TYR, ACADSB, ACTN3, FGF10, NEFL, TAC4, KCNK4, SCN3B, AKR1D1, SCN9A, SCN8A, TAC1, VCP, UCN2, CDH3 |
| SX24 | Normuscone | SCN11A, SCN3A, ALDH5A1, SCN4A, SCN1B, SCN2B, SCN7A, SCN2A, SCN4B, OGDH, ABAT, SCN5A, HDAC9, SRD5A2, HDAC2, SCN1A, SCN10A, TYR, ACADSB, SCN3B, AKR1D1, SCN9A, SCN8A |
| SX25 | 2,6-Nonamethylene Pyridine | GABRB2, SDC4, GABRB3, CD47, F2, BICD1, GPR27, CD36 |
| SX26 | 5-Cis-Cyclotetradecen-1-One | PDE7B, CYP17A1, PRKDC, PIK3CA, PIK3CB, ITPR3, ATM, CYP19A1, PDE11A, PIK3R1, ADA, TRPV1, PTGS2, TNFSF11, PHB, TOX3, GNAT1, KHDRBS1, SPI1, XRCC4, HCN4, GNAT3, YWHAE, NUDT9, AGT, CXCL13, PTK2B, ADRB1, LRRC8A, GHRL, C5, CHRNA3, HSP90AB1, SLC6A4, TET1, KCNH2, MTOR, ZFPM1, ZFP42, CAMK2D, ATP1A1, SMO, AQP1, FKBP1B, ATP2B4, HPS4, RYR2, GPER1, XRCC6BP1, NOS1, SOX9, CAMK2G, GNAS, TRIM24, NFX1, PDE5A, PDE3A, PIK3CD, ADORA2B, ITPR2, POLA2, PDE6B, PDE8B, PDE8A, WNT4, HAP1, AR, ELOVL4, NT5C1A, SIRT1, UTS2, SIX1, PPP3CB, HOMER1, MED1, EDN1, HELB, CETN2, PIK3CG, FER, ARID1A, LIG4, ADAM8, FGFR2, BCL11B, SREBF1, TCF3, HIBADH, HMGCR, C2CD5, MMP28, WNT10B, GATA3, PIK3R6, ADRA1B, RYR3, RAG1, TNFAIP3, CDC42, CTNNB1, TKT, CD34, CBFA2T3, PARP10, TREM1, BMP5, LAMP2, NCBP2, REST, TAS1R2, PDE9A, ADORA2A, ESR1, PDE1A, PDE1B, PDE4C, PDE4B, NT5E, NR3C2, TACR2, IDNK, FADS2, HDAC1, ACTN3, DGKI, STUB1, QDPR, SYT2, SHANK3, NKX3-1, CACNA1A, COMT, POLA1, BMP2, SHH, FGF10, ADRBK1, VTI1A, AURKA, BCL2, DRD2, SRC, STAP1, HIPK2, CSF2, HIF1A, SLC18A3, UBR5, PF4, IL10, PPP3R1, RAB8B, TAC4, IL4, PGLS, DRD1, HSP90AA1, CALHM1, HPRT1, TAL1, PDGFB, NEFH, TPM1, CRHR2, TFAP2C, PDE4A, ITPR1, PDE3B, PDE4D, OPRK1, PDE10A, PDE2A, HDAC2, RINT1, AMPD3, SLC8A1, FADS1, HMGA2, NOS1AP, FKBP1A, CTR9, TGFB1, SIX4, TAS1R1, UBE2B, STAT5A, SLC26A6, CRP, TNF, SERPINB3, KCNB1, MAOB, SLC44A4, MCM3, PLN, LONP1, HRC, PPP3CA, TALDO1, KIT, ABCC4, AREG, ATP1A2, NEFL, SPX, DKK3, DPPA3, POLB, SLC9A3R1, ADAP1, LTA, WNT5A, AMICA1, ALAD, RTN2, GLYR1, PIK3R5, HCN2, TAC1, FURIN, G6PD, ATP11C, SIRT2, TRIM28, ESR2, TP53, ADRA2A, CETN1, NEDD4, FOXP3, LEF1, SORCS3, ADAP2, VCP, GAS6, DNM3, XCL1, UTS2R, RAPGEF2, BDKRB2, INS, BNIP3, CRH, AVPR2, UCN2, ATP2A1, AHCYL1, CASQ1, IDO1, IGF2, EDA, SPR, SELP, TAS1R3, PAWR, CD63, VDR, PCSK6, RAG2, RYR1, PDE6A, PGD, PGR, PDE7A, PDE1C, ADORA1, PDE6C, RIPK1, CX3CR1, F12, PTGS1 |
| TBC1 | Benzoylaconine | SLC6A3, SCN11A, CHRM1, SCN5A, SCN10A, SLC6A4, HRH1, CHRM2, SLC6A2, CYP3A4, PADI4 |
| TBC2 | Pennogenin | ATP1A1 |
| TBC3 | Penniclavine | ADRA1A, HTR2A, HTR2B, ADRA2A, HTR1B, HTR2C, HTR1D, DRD2, DRD4, HTR7, HTR1F, HTR1A, ADRA2C, DRD3, DRD5, HTR1E, DRD1, ADRA2B, CHRM1, CHRNA2 |
| TBC4 | Penduline | SLC6A3, SCN11A, CHRM1, SCN5A, SCN10A, SLC6A4, HRH1, CHRM2, SLC6A2, SLC18A1, SLC18A2, ACE |

*****: Unadopted due to ADME screening

**Supplementary file 4**

**Table S4 Targets information of WSP and gout**

| **Source** | **Targets** |
| --- | --- |
| WSP (1652) | PDE10A, PDE1B, CAMK2G, PDE4B, PLN, PDE11A, PDE4D, HRC, PDE5A, CAMK2D, PDE9A, PDE2A, TRPV3, ESRRG, AKR1C2, FECH, COX6B1, COX8A, ADRA2A, BNIP3, TACR2, NEFH, OPRK1, COX6C, COX5A, COX4I1, FABP6, NR1H4, TRPV1, CRH, AQP1, CRHR2, TRPM8, COX5B, COX3, PLA2G1B, CES1, ACTN3, FGF10, NEFL, TAC4, TRPA1, COX7C, AR, COX6A2, COX7B, TAC1, VCP, UCN2, CDH3, KCNK4, COX1, COX7A1, ADH1C, COX2, NGFR, HIF1A, SMO, CBFA2T3, ESR1, PHB, ESR2, FGFR2, GPER1, TRIM24, PGR, TOX3, NEDD4, SRC, WNT5A, WNT4, TGFB1, SHH, UBR5, SOX9, TFAP2C, MED1, SERPINB3, AREG, PDGFB, TNFSF11, NKX3-1, LEF1, CD34, VDR, PDE7B, CYP17A1, PRKDC, PIK3CA, PIK3CB, ITPR3, ATM, CYP19A1, PIK3R1, ADA, HMGA2, FURIN, G6PD, ATP11C, SIRT2, TRIM28, TP53, CETN1, FOXP3, SORCS3, ADAP2, GAS6, DNM3, XCL1, UTS2R, RAPGEF2, BDKRB2, INS, AVPR2, ATP2A1, AHCYL1, CASQ1, IDO1, IGF2, EDA, SPR, SELP, TAS1R3, PAWR, CD63, PCSK6, RAG2, PDE3A, PIK3CD, ADORA2B, ITPR2, POLA2, PDE6B, PDE8B, PDE8A, HAP1, GNAT1, KHDRBS1, SPI1, XRCC4, HCN4, GNAT3, YWHAE, NUDT9, AGT, CXCL13, PTK2B, ADRB1, LRRC8A, GHRL, C5, CHRNA3, HSP90AB1, SLC6A4, TET1, KCNH2, MTOR, ZFPM1, ZFP42, ATP1A1, FKBP1B, ATP2B4, HPS4, RYR2, XRCC6BP1, NOS1, GNAS, NFX1, ADORA2A, PDE1A, PDE4C, NT5E, NR3C2, IDNK, NT5C1A, SIRT1, UTS2, SIX1, PPP3CB, HOMER1, EDN1, HELB, CETN2, PIK3CG, FER, ARID1A, LIG4, ADAM8, BCL11B, SREBF1, TCF3, HIBADH, HMGCR, C2CD5, MMP28, WNT10B, GATA3, PIK3R6, ADRA1B, RYR3, RAG1, TNFAIP3, CDC42, CTNNB1, TKT, PARP10, TREM1, BMP5, LAMP2, NCBP2, REST, TAS1R2, PDE4A, ITPR1, PDE3B, HDAC2, RINT1, AMPD3, HDAC1, DGKI, STUB1, QDPR, SYT2, SHANK3, CACNA1A, COMT, POLA1, BMP2, ADRBK1, VTI1A, AURKA, BCL2, DRD2, STAP1, HIPK2, CSF2, SLC18A3, PF4, IL10, PPP3R1, RAB8B, IL4, PGLS, DRD1, HSP90AA1, CALHM1, HPRT1, TAL1, TPM1, RYR1, PDE6A, PGD, PDE7A, PDE1C, ADORA1, PDE6C, RIPK1, CX3CR1, NOS1AP, FKBP1A, CTR9, SIX4, TAS1R1, UBE2B, STAT5A, SLC26A6, CRP, TNF, KCNB1, MAOB, SLC44A4, MCM3, 8-Mar, LONP1, PPP3CA, TALDO1, KIT, ABCC4, ATP1A2, SPX, DKK3, DPPA3, POLB, SLC9A3R1, ADAP1, LTA, AMICA1, ALAD, RTN2, GLYR1, PIK3R5, HCN2, NR3C1, PRLR, ANXA1, SRD5A1, PTGFR, PTGER1, PTGIR, FADS2, FADS1, PTGS1, SLC8A1, PTGS2, ELOVL4, DHFR, INSR, DPYD, ADK, ATIC, DMTN, P2RX2, HOXA5, IL1B, CMPK2, CHRNB2, AK9, HYAL2, CHRNB4, CACNA1C, KCNMA1, TBPL1, HTR2A, FBLN1, NT5M, CDKN1A, DNMT3B, MGMT, TRDMT1, TYMS, ABCC2, DNMT3A, SLC22A6, P2RX3, DPYS, DUT, DTYMK, DHFRL1, SHMT1, FOLR2, ASNS, FOLR1, DNMT1, GABRB2, CD47, GABRB3, BICD1, F2, NR1I2, GPR27, CD36, S1PR5, SDC4, SCNN1A, TLR8, HRH1, SCNN1D, TLR7, SCNN1B, SCT, ACACA, PRKAA2, CALCOCO2, OXT, HTR2B, TLR3, ADI1, HTR6, MAS1, ORMDL2, MAOA, NCOA3, UGT8, PYGL, PID1, NMI, RFK, P4HA2, RPS6KB1, ABCG1, KCNJ8, ADRB2, PRKAA1, ADRA2C, KCNJ1, CYP2B6, UGCG, CHRNA7, HTR2C, SLC22A1, ACTN2, RSAD2, NAV2, GAA, PC, WNK4, SPIDR, BLM, ENOPH1, BMP6, KCNJ11, MZB1, SGK1, MRI1, TYMP, HRH4, AGTR2, DECR2, PIK3AP1, ZP3, NACA, KCNE2, RGS2, GDPGP1, EDN2, RAD51, ADRA1A, ACACB, APRT, HTR7, AOC3, AGTR1, ADRA2B, PHKG2, PNP, AOC2, SCN3A, C1QTNF1, GBA2, GBA, DLL1, LHCGR, SNX3, CLN3, SERPINF2, HRH3, MSTN, NMUR2, PYGM, KCNJ10, REN, SEC14L2, PCCB, EGFR, SYK, PAICS, SCN1A, MTAP, ADRA1D, HRH2, P4HA1, ADRB3, SLC6A2, APLP1, ARRB2, ALDH3A2, ARRDC3, SCN2A, APIP, PCCA, GSK3A, KLHL3, VCAM1, CECR1, POR, MCCC2, P2RY1, GAL3ST1, ARRB1, TLR9, ABCC9, PDGFA, DRD5, POU1F1, ADAM17, UNC93B1, MECR, PYGB, ATP8A2, SCNN1G, PECR, SRPK2, ACP1, SAXO1, RAB7A, CALCA, ADCY6, ORMDL1, ANG, FGF1, RXFP4, GOT1, MCCC1, UCHL1, AGTRAP, AOC1, TNNC1, ACE, CAV1, GPR101, MLYCD, ZNF219, P4HA3, P4HB, PRKCD, OPRD1, OPRM1, WLS, PTGER3, PTGER4, ADH1B, F12, PTGER2, AKR1C1, TYR, CNR2, CNR1, KCND1, GAMT, CAT, KCNC1, KCND2, KCND3, KCNQ1, CYGB, IGF1, CNTNAP4, GRIN2A, SPARC, CRLF1, ALDH1B1, DARS, MIP, STX1A, NRXN2, DAB2IP, KCNA3, KCNC3, ALDH2, GUCY1B3, FBP1, NRXN3, RNASE4, SLC17A7, FADD, LEP, ANK3, PAXBP1, CYP2E1, PRKAB1, KCNA1, KCNB2, KCNA4, GATM, LRRC4B, ACY3, MAGI2, RAB3A, CYP11A1, RNASE8, SNTG2, NLGN1, KCNE5, ZPR1, PAX7, ADH4, ACY1, ADH1A, KCNA2, DLG4, KCNA5, KCNA7, ADH7, AQP8, ASPA, APOE, MC4R, SCN5A, KCNIP2, SCGB1A1, CACNA1D, PPARD, ALDH3B1, ALDH3B2, GJA5, KCNA10, TPO, KCNC2, RNASE1, KCNA6, IYD, NFIB, ARX, NRXN1, SOX15, SCN10A, GPX7, OXTR, NKX2-1, NPPA, RNASE2, FAS, CACNA2D1, LIPA, CES2, CES5A, CES3, ESD, GGCX, NDRG2, METRNL, WNT11, SNCA, CCM2L, NAMPT, MAPK9, ALOX15B, CPLX2, RGCC, PTGIS, NEUROD2, CDC20, ALOX15, SERPINB7, FNDC5, HEG1, NAPRT, AVPR1A, AVP, ANAPC2, MECOM, PRDM16, TOP2A, TUBA3C, TOP2B, TUBB, TUBA1C, TUBA3D, TUBB4A, TUBB2A, TUBA1A, TUBB2B, TUBA1B, TUBB4B, TUBA4A, HTR1B, CACNA1I, CHRM2, KCNJ12, CACNA1H, CACNA1S, HTR1F, CHRM4, KCNJ15, HTR1A, KCNJ14, CACNA1F, SLC18A1, CHRM3, CACNA1G, HTR1D, DRD3, SLC18A2, CHRM1, CHRM5, ALOX5, SERPINE1, TOP1, HDAC9, SCN4A, SCN1B, SCN11A, SCN3B, AKR1D1, SCN4B, SRD5A2, SCN2B, ALDH5A1, ACADSB, OGDH, ABAT, SCN7A, SCN8A, SCN9A, CKMT1B, RARB, ALDH1A1, ALDH1A2, SLC9A1, LPCAT1, OPN3, SLC8B1, PLA2R1, MAP4K4, OPN4, PNPLA2, CNST, SLC8A3, MYOD1, SP3, SCD5, JUNB, MEX3C, ABCA1, ABHD5, FABP3, CYR61, ACSL4, RARRES1, RXRG, RARA, APOA2, IL13, COL27A1, HOPX, NR1H3, FMR1, ITGB3, ANXA13, STX3, CAPN3, GDF5, PPARGC1B, ELOVL6, ZNF536, COL1A1, NOTCH1, TSPO, NODAL, CYP1A1, ACSL3, NR0B1, SLC6A8, CKB, ALDH3A1, LARP4B, OSBPL8, ELOVL3, FOXA1, EIF2AK1, SLC8A2, ABCF1, ITGAV, PTPN2, PAX2, NR1H2, NR1D1, ELOVL7, WDR77, GJD4, SLC35G1, DHRS9, FGF2, PPARG, RXRB, RARG, CKMT2, GPRC5A, SC5D, FASLG, ELOVL1, NUFIP2, OXER1, CYP4F2, ALDH8A1, SERPINH1, GNAT2, S100A8, SP1, JAK3, DNAJA3, SLC3A2, PPARA, S100A9, FFAR1, CKM, CKMT1A, RXRA, SLC16A12, HSD11B1, IHH, RPL7A, SHBG, NPPC, MATN1, ALOX5AP, FGF4, GNB2L1, TBC1D32, RET, SCD, PLCB1, ADIPOQ, MYL2, SEC14L3, PPP2CB, CHUK, PPP2CA, PRKCA, DGKA, MPO, PRKCB, SEC14L6, DDC, SEC14L4, PLA2G2E, IKBKB, SUCLG2, SLC25A10, SUCNR1, OXCT1, SDHA, DCT, JMJD6, HSD17B11, GRIN3B, CACNA1B, GRIN3A, GRIN1, STIM2, COLGALT1, ACOT4, SLC25A12, CDH11, SMAD7, STAR, HTT, TST, TMEM110, EPHA4, UGT1A1, PLOD2, SLC25A1, SLC13A2, P3H3, SLC13A3, PLOD3, AKR1C3, SLC13A4, TYRP1, CACNA2D2, GRIN2C, SLC7A1, BDH1, CYP1A2, PKD2, EGLN2, PHGDH, YBX3, PRPH, PRDM8, OGFOD1, DLST, AP3D1, SLC1A6, OCA2, HACL1, CASQ2, DLD, SLC13A1, OXCT2, ASPH, SUCLA2, CRTAP, SDHAF2, HIF1AN, PLAT, GRIN2B, SLC7A4, GPD1L, SUGCT, COLGALT2, SUOX, CCL5, ALDH9A1, ACAT1, PNLIPRP2, BCKDHB, UROD, SLC38A7, MYO5A, EGLN3, ARHGEF2, HSD17B8, SULT2A1, SUCLG1, TMLHE, BBOX1, SDHC, SDHD, SLIT2, SLC1A3, UROS, SLC7A2, SLC7A3, GRIN2D, ACSS2, CCL2, ANK2, TH, DHODH, GALC, CYP39A1, STIM1, DHTKD1, ACSS1, CRACR2A, ILVBL, OGDHL, ACADM, EGLN1, SLC25A13, HSD17B6, PLOD1, SDHB, P3H2, P3H1, ACO2, SALL1, SLC13A5, PLG, KARS, FBXO45, ARV1, MRPS36, BCKDK, HSD17B2, EPO, PLCG2, BCKDHA, NEDD4L, SRD5A3, ETHE1, CYP4B1, HSD17B1, CAV3, SQRDL, ETFDH, PCSK9, GPR143, SFXN5, DBT, TUBD1, TUBB3, TUBB6, SLC38A3, TUBG1, EHHADH, TUBB8, DAB2, TUBE1, TUBA8, JUN, TUBG2, TUBB1, ABCB1, TUBA3E, PCK1, CALM2, GLTP, CALM3, CALM1, HTR3A, BCHE, ACHE SLC18A2, CHRNA2, GABRA2, GABRA3, GABRQ, GABRD, GABRG1, GABRG2, GABRB1, GABRP, GABRA5, GABRG3, GABRA4, GABRA6, GABRE, GABRA1, ITGB2, ITGAL, HBA1, CREBBP, RELA, VAV3, PRMT2, PIAS1, USF1, PTEN, NSD1, CITED1, PDLIM1, ARHGEF15, SMAD3, FHL2, GADD45G, CCND1, BCAR1, HNF4A, PRMT1, MAPK3, RFX4, MTA1, MED21, SAFB2, MED16, SMAD2, EIF3I, CRMP1, RBM39, COPS5, GNAI1, MED6, EIF6, SOS1, NCOA1, PHB2, NPDC1, NCOA2, BAG1, PTGES3, NOS3, CDK7, RNF14, TCF20, KAT5, GTF2H1, MKNK2, WDR5, BTF3, RPS6KA3, CACNG1, DBH, SLC6A3, AKT1, DDX5, XBP1, NCOR2, MAPK1, CDC25B, CLIC1, POU4F1, LCK, DDX17, MAPK14, BAZ1B, CCNH, MED17, STAT3, TDG, FKBP4, UBC, SVIL, FDXR, ZBTB16, PSMC5, NR2C2, CUL4B, UBE3A, HEXIM1, PPID, IGF1R, PTPN1, ASH2L, HSPA4, TRIM59, MED10, RGS3, POLR1B, FOXO4, DDX54, SHC1, PIAS3, SMARCE1, PAK1, SLC35F6, ZNF398, DAP3, CPT2, MTCH2, PAGR1, CYBA, FHL1, NFKB1, NR0B2, SMARCD3, PRADC1, IGSF21, MED7, CCNC, MAP3K1, CEBPA, KDM5A, PPP5C, HSPE1, TBP, MMS19, HSPA5, PTMA, UBE2I, RBFOX2, RNF4, FKBP5, ARNT, TRIP4, ETFB, CCNE1, MED23, MDM2, MTA3, FLII, GTF2B, HSPD1, CDK2, CUEDC2, PIAS2, LDB1, MED13, CEBPB, MED14, SMARCA4, POU4F2, DNTTIP2, NCOA4, PRKACA, GRB2, HBA2, MED12, PAK6, PPARGC1A, ITGB3BP, ZBTB17, MED20, NELFB, EP300, NCOR1, SLC30A9, MPG, CHD9, PSMB9, ESRRA, PIK3R3, NCOA7, RBM23, CCNT1, TRAM1, RLIM, THRA, UNC119, PRPF6, POLR2D, TNFRSF14, ERBB2, MNAT1, POU2F1, DRD4, YWHAH, C14orf1, GADD45A, TRRAP, BDNF, FOXO3, NR4A1, PTPN6, NR2F6, PPP1CC, GADD45B, MED24, AKAP13, KIF1A, FHIT, BCAS2, CPT1A, MVP, MDH2, FAM45A, ISL1, SMAD4, BRCA1, PRDM2, KMT2D, CDK8, REXO4, MTA2, WIPI1, KBTBD7, PNRC2, CSNK2B, PELP1, STRN, AHR, POU2F2, GRIP1, RPS6KA1, TSC2, SAFB, NCOA6, NRIP1, RBBP5, YWHAQ, SMARCD1, SMARCA2, TAP1, JUND, NR2F1, THRB, TADA3, NR2C1, TAF10, CRIPAK, ATAD2, FOXO1, RPL13A, TRIM25, AKT2, CD300A, CHGA, APOC2, KIF14, RDH11, RDH5, RDH10, ASCL1, RBP3, ALDH1A3, RLBP1, DLX5, OPN5, RETSAT, RDH12, RDH14, RHO, RDH13, DHRS4, RDH8, RS1, CTSH, DHRS2, CBR3, CBR1, DHRS3, RBP1, LRAT, HSD17B7, CRYM, PADI3, DDAH2, PADI6, TFAP2B, OAZ3, OAT, SLC1A5, BCAT1, SLC1A4, GLRA3, GLYATL1, GPT, SLC6A9, AGXT, IARS, GLRA1, DGAT2, SLC24A2, MTRR, BAK1, FCER2, F2RL1, NAGS, PADI2, PADI4, PADI1, HLCS, OAZ2, GOT2, SLC1A1, GCAT, ADSSL1, DARS2, GNMT, GPR18, LARS, GLDC, AARS, ASRGL1, LARS2, SLC6A5, BCAT2, LYZ, KLRC4-KLRK1, ROBO1, KLRK1, NOS2, AZIN2, ARG2, MAP1S, SLC25A15, SLC25A2, GPT2, GCSH, IARS2, VDAC3, GLRB, CTPS1, NARS2, VDAC2, SHMT2, GLYATL2, LCMT2, GPM6B, EDN3, BCL2L11, SLIT3, AIF1, CERS1, DDAH1, OTC, ASL, HMOX1, CAD, OAZ1, BAAT, SLC7A8, GLRA2, VARS, GSS, LCMT1, GLYAT, PIPOX, ALAS2, SLC32A1, ADSS, GLUL, KYNU, ECE1, NDOR1, SLC11A2, BAD, ASS1, KLF4, ARG1, CPS1, PPAT, ALAS1, NFS1, AARS2, PHYKPL, GARS, VDAC1, SLC36A1, AGXT2, NARS, RARS, HMBS, IFNG, MT3, LCN2, GNA15, ARFGEF2, P2RX1, DOCK5, DOCK4, NTSR1, MAP2K1, ACHE, NRG1, CDH8, DMGDH, ALDH7A1, FNTA, COLQ, SIX3, AGRN, CHKA, SLC5A7, PRSS12, ENPP6, CHDH, GFI1, KL, CYP27A1, CYP27B1, SNAI1, LANCL2, CYP2R1, KANK2, GC, CYP24A1, CYP3A4, PML, IRX5, SNW1, GPBAR1, CALB1, B4GALT1, SNAI2, FGF23, BAX, S100G, SLC25A20, GABBR1, CDK5R2, EPCAM, PHOX2B, ADCY3, LRRK2, KDM6B, THNSL1, SLC22A5, F11, GRM7, EPHX2, CRAT, ARPIN, TBR1, MAD2L2, FOS, SLC25A29, TARS2, XDH, ARCN1, CEND1, CDK5R1, ATP13A2, RERE, PARK2, NLGN3, TARS, SLC22A4, CROT, KLKB1, ABHD6, DAGLA, MGLL, FCER1A, PLIN5, FCER1G, SUMO1, RNF207, CCR7, GPR55, C3, LTK, CLEC4M, CAMK2A, TIGAR, VDR , SOAT1, TNK2, APAF1, SLC25A5, ANPEP, ACVRL1, ACVR1B, ABCC8, ARAF, SLC25A6, NPC1L1, ACVR1, ADCY1, ABL2, ABL1, ACSL1, ALK, SLC25A4, ASNA1, NT5C2, AFG3L2, ABCB11, ADRBK2, AMHR2, CDK15, NAE1, AKT3, CALR, ASMT, EPX, NQO2, MTNR1A, MTNR1B, NOTCH2, CDK6, DAXX, KDM1A, ADAM10, HDAC3, RAC3, PARK7, HSPA1A, MAGEA11, PSPC1, PMEPA1, PRDX1, KAT2B, RNF6, TMF1, HDAC4, PIAS4, ERG, SPDEF, PPIA, CREBRF, FXYD2, CASP8, HMGB2, CASP3, RAF1, CDC37, PSMC3IP, WHSC1, SELENBP1, GTF2F1, PA2G4, AES, SRY, HMGB1, ACTB, NISCH, TRIM68, POLR2A, RAN, CDK9, TGFB2, RANBP9, KLK3, CDC25A, PARP1, RUNX1, ATF2, PATZ1, PXN, IL6ST, UXT, KLK2, PNRC1, RB1, ETV5, TGIF1, HIPK3, NONO, EFCAB6, CASP1, CAMLG, PPIF, GAPDH, XRCC5, TCF4, GAK, SART3, KAT7, TGFB1I1, APPBP2, APOL2, CDK1, GSK3B, JMJD1C, GTF2F2, HSPB2, SMAD1, RBAK, XRCC6, DCAF6, ATP1A3, COL5A1, NR1I3, RUVBL1, FLNA, POU5F1, MYBBP1A, IFI16, TSG101, RUNX2, CASP7, SMARCC1, RCHY1, CTDSP2, SIN3A, RAD9A, IDE, DCC, ZMIZ1, NR5A1, GSN, RAD54L2, RNASEL, PPP3R2, NOX1, ATP1B1, ROCK1, NCKAP1L, IKBKE, DHDH, AKR1C4, TPMT, ROCK2, AKR1B10, MAPK11, SDF4, MAD2L1, SLC12A5, ABCB4, SLC12A1, SLC12A2, NFATC4, MAPK7, ATP6AP1, NUCB2, SLC12A7, ESRRB, SLC12A4, SLC2A1, ABCG2, FGR, SLC12A6, GRIA2, NPY5R, GRIK5, GRIK1, ADORA3, CHRFAM7A, CHRNA9, GRIK4, GRIK2, GRIA4, GRIA1, KMT2A, KISS1, CHRNA4, GRIK3, ADIRF, GRIA3, CRYZ, VKORC1, NQO1, KCNH6, KCNN4, KCNMB4, CYP21A2, KCNMB1, POMC, SQLE, KCNH7, KCNN1, KCNN3, KCNN2, KCNMB2, KCNMB3, PROS1, PROC, MGAM, TAAR1, HTR1E |
| Gout (718) | PRPS1, SLC17A3, LDHD, CMTX5, NPT4, DLACD, DFNX1, UAQTL4, DFN2, GOUT4, AMPD2, SELE, HPN, LEP, PIK3R1, PIK3CA, HNF1B, UGT1A1, UMOD, HPRT1, GCKR, PKD1, TMPRSS2, PIK3R2, TNFRSF25, LRP6, ABCG2, SCD, CORIN, SLC2A9, FADS1, NLRP3, NAT8, TMPRSS5, ACADS, ACADM, RPS6KB1, RPS6KB2, COASY, ARHGEF25, XPNPEP3, SLC16A9, GPATCH8, XDH, SLC22A8, SLC22A7, AOX1, SLC22A2, CYP2C9, CYP2C19, SLCO1B1, SLCO2B1, ABCB11, AKR1B1, CS, SRC, RNASE1, IDH1, RNASE3, PGLS, IL4I1, CTDSP1, HGS, ITEVIR, MDH2, CPB1, RTCA, LSM6, PLEKHA1, C8G, APRT, GMPS, ITPA, MC2R, CRH, HSD3B2, CYP27B1, CYP3A4, PTGS1, PTGS2, TTR, CYP1A2, CYP2C8, ABCC4, ABCC1, SLC22A6, SLCO1C1, SLC22A11, UGT2B7, CYP2B6, CYP2C18, CYP2E1, UGT2B4, SCN4A, ASIC1, KCNQ2, KCNQ3, PLA2G2A, CYP2D6, SLCO1A2, BCL2, THBD, FABP2, PPARG, CFTR, PPARA, GP1BA, S100A7, AMACR, SLC22A12, CA1, NR3C1, CYP2A6, CYP1B1, ANXA1, SLC22A1, ABCC3, ABCC5, SLC16A7, SLC22A5, ABCC6, SLC16A1, SLC10A1, SLC22A10, ABCC2, ABCC11, PANX1, TAS2R16, SLC25A21, SLC13A2, SLC13A5, SLC25A1, NR1I2, MAPK3, PPARD, PTGDR2, CYP1A1, AKR1B10, UGT1A6, UGT2B15, SULT1A1, NAT2, PTGES3, TRPV1, FAAH, GSTP1, GSTM1, CYP3A5, CYP3A7, SERPINA6, SCN10A, PTGER1, NOS2, NR0B1, CYP17A1, CYP3A43, CYP4A11, CYP11B1, HSD11B2, HSD11B1, SHBG, CYP11B2, SRD5A2, PTGR2, GLO1, CES1, AKR1C3, SERPINA7, BCHE, TUBB, DARS2, SLC17A1, MUC1, IL1B, INS, ALDH2, ALPK1, PDZK1, TNF, CXCL8, MEFV, P2RX7, ADSL, CCL2, IL6, IL18, CARMIL1, S100A9, ALB, IL1R1, TLR2, TLR4, ALDH16A1, PYCARD, SLC22A13, MC3R, HNFJ3, SLC37A4, SHLD2, PRPS1L1, ANKH, G6PC, DNAJB11, PPAT, PRPS2, PFKM, SEC61A1, NT5C2, GRHPR, MSMB, APOE, REN, MAOA, NPHP1, RELN, FXYD2, PAX2, SLC4A1, SLC12A3, CLCNKB, ANK1, SPTA1, SPTB, EPB42, PNP, NPHP4, KCNJ16, SCARF1, RMND1, SHROOM3, ARHGAP44, SPATA5L1, CST9, CCDC160, NGF, LPL, PRTFDC1, HNF1A, APP, SLC6A3, FGA, ATIC, ERCC6, RAPGEF3, GDA, RAPGEF4, TMSB15A, LOC107032760, JAK2, MPL, CPT2, AGL, GBE1, TET2, APOA1, CSF2, PYGM, LIPC, TNFSF11, KCNJ11, ABCC8, APOB, TGFB1, IL4, IL10, IL17A, CSF1, HP, HLA-B, LPA, HNF4A, AMPD1, AMPD3, STAT3, BLK, GCK, FBP1, ENO3, PDX1, PFKP, PCBD1, PHKA2, NEUROD1, PFKL, PGAM2, HFE, PAX4, UBA5, LHX1, GCG, FHL3, G6PC3, CLIC1, CDH4, NEK3, NFE2, PTF1A, UFM1, KLF11, CDK5RAP3, OTULIN, DDRGK1, SLC37A1, NRAP, SLC37A3, SPNS1, UFL1, PPP1R3D, MYOM3, MYOZ3, PAGR1, LIME1, CLTRN, MIR142, TNFRSF1A, CCL3, HLA-DRB1, IGF1R, PTPN11, MMP3, VEGFA, PKLR, FGF5, F2, CTSL, TNFRSF11A, MMP1, G6PD, IFNG, GATA1, COX5A, HBG2, BCAS3, TNFRSF1B, CRP, MMP9, KCNJ1, SAA1, HMGB1, CXCL2, COMT, PRKG2, IL1RN, IL1A, LRP2, PDGFRB, FLT3, LDLR, NOD2, PTH, VCAM1, UGT1A8, CARD8, MAF, WDR1, CNTN5, ZNF724, MIR302F, CD14, NIPAL1, PDGFRA, EZH2, STAT1, KIT, ABL1, CSF1R, IDH2, TYK2, CALR, FLT1, ADAM17, DNMT3A, PRKG1, JAK3, FOS, MMP13, CASR, ICAM1, PTPN6, ITGB2, TEK, JAK1, MIF, AGT, ACTB, RIPK1, ITGB3, JUN, STAT5B, EPOR, CTSK, GAPDH, COL2A1, OPRM1, PIK3CG, IRF5, TFRC, YY1, VWF, BCL2L1, ACP5, CD44, COMP, MMP8, CD28, CSF3R, SLC11A1, HSPA5, ETV6, ANXA5, FOXP3, CTCF, CETP, MMP10, NR4A2, PAX5, PRDX2, SLC12A1, REL, PTPN22, WNK1, EPB41, CCR2, F5, AQP1, ANGPT1, ACAN, CXCL12, CTLA4, GYPA, CIITA, HBB, SERPINH1, IL6ST, TNFSF13B, STAT4, STAT5A, PSTPIP1, TRPM6, SELP, TIMP1, CCR6, APOA5, CR1, RHAG, HGD, HMGA2, HLA-DQB1, MMP17, MMP12, IL17RA, IL13, PADI4, SOCS3, NLRP1, SNCAIP, RPS3, TNFSF13, KITLG, KLF1, STK24, IL3, KCNJ10, STK39, THBS1, WNK4, CCL5, CCL20, ASXL1, BCL11A, CXCL1, HS6ST1, CD47, GYPC, CD86, CD34, RGS2, PRKCSH, SEC23B, SEC63, SDC1, RPS27A, SPTBN1, OSM, PMPCA, USF1, ADAMTS4, MIPEP, ANXA7, LTA, ADAMTS5, HS3ST1, HLA-DQA1, HBG1, RPS13, SRI, LAMA5, THPO, ANK2, EPO, FSTL1, CLDN16, CD80, PF4, IFNA2, RPS15, OSGEP, RPS3A, PTGES, U2AF1, KLHL3, OXSR1, ADD2, GLCE, BSND, CXCL5, CXCL6, HS2ST1, HS3ST2, CSF3, RHD, CLCNKA, IL15, RPS23, PGLYRP1, TNFAIP6, STEAP4, KDM4C, MICA, CD177, BLOC1S6, HS3ST3B1, CTCFL, IL23A, IFNA1, SACM1L, IMMP2L, PVALB, LDB1, S100A12, WNK3, FIP1L1, HBD, HBE1, RPS21, NPRL3, TTC21B, LDB2, HS3ST3A1, GYPB, PIEZO1, ZFPM1, FCRL3, HS3ST4, DMTN, HS3ST6, OLAH, LACC1, VEZF1, HS3ST5, CYLC1, SFTA3, OR51V1, CAVIN3, OR51M1, H19, H2AC18, MACIR, MIR150, H3-2, IGHV4-38-2, SLC5A4, PTGIR, ANO1, URAT1, FABP1, CXCR2, CXCR1, ZNF518B, PPM1G, PDE1C, RARB, PRKCA, MAPK6, RAB27B, MEPE, TRIM54, PTPRD, MAP4K2, ALX4, RBFOX1, ATP1A4, MPV17, MAP3K11, CRIP3, OR7E35P, KRT8P26, KCNQ1, ORC4, SLC22A18AS, PKD2, SFMBT1, PRKAG2, MLXIPL, OVOL1, LHFPL3, RREB1, SLC5A6, ALDH1A2, TPST1, ZNF512, C2orf16, RNASEH2C, QRICH2, INHBE, H4C2, BAZ1B, AP5B1, SNX17, ABCG1, BABAM2, MRPL33, NRXN2, UBE2Q2, BCL7B, ZMYM6, ACVR2A, H4C3, H3C2, H2BC4, SLC13A3, FNDC4, FAM86MP, ZSCAN31, ATXN2, SPP1, H2AC4, H2AC6, WNT5B, TRIM46, VDR, BDKRB2, JAZF1, NRG4, ZNF513, CLNK, H2BP5, GPN1, NXPH4, CD160, FAM53A, H2AC1, RFX3-AS1, KRTCAP3, NUDT17, ABCA1, LINC01010, NRXN2-AS1, CT69, AADACL2-AS1, ABCC9, MUC22, SLC17A4, H2BC2P, ADPGK-AS1, SH2B3, ALG1L3P, LINC01405, LINC01229, B4GALT1-AS1, POLR3C, SCGN, RELA-DT, PPM1K-DT, KAT5, TRIM38, BAIAP2, H2AC5P, SLC17A2, LINC01460, FGFR2, INHBC, NRBP1, GTF3C2, MACROD1, HNF1A-AS1, RNF115, TBL2, A1CF, H1-2, H1-6, RAF1P1, HNF4G, INSR, HLF, LINC02537, HGF, FBXO22, IFT172, FRK, MLXIP, CUX2, FSTL4, SLC4A1AP, CNIH2, H2BC1, NALCN, R3HDM2, MT1B, MTTP, ADIPOQ, CXCL16, NR2C2, POMC, MAP3K7, SLC40A1, SIRT1, IL33 |
| Common targets (165) | LEP, PIK3R1, PIK3CA, UGT1A1, HPRT1, ABCG2, SCD, FADS1, ACADM, RPS6KB1, XDH, ABCB11, SRC, RNASE1, PGLS, MDH2, APRT, CRH, CYP27B1, CYP3A4, PTGS1, PTGS2, CYP1A2, ABCC4, SLC22A6, CYP2B6, CYP2E1, SCN4A, BCL2, PPARG, PPARA, NR3C1, ANXA1, SLC22A1, SLC22A5, ABCC2, SLC13A2, SLC13A5, SLC25A1, NR1I2, MAPK3, PPARD, CYP1A1, AKR1B10, PTGES3, TRPV1, SCN10A, PTGER1, NOS2, NR0B1, CYP17A1, HSD11B1, SHBG, SRD5A2, CES1, AKR1C3, BCHE, TUBB, DARS2, IL1B, INS, ALDH2, TNF, CCL2, S100A9, PPAT, NT5C2, APOE, REN, MAOA, FXYD2, PAX2, PNP, SLC6A3, ATIC, CPT2, CSF2, PYGM, TNFSF11, KCNJ11, ABCC8, TGFB1, IL4, IL10, HNF4A, AMPD3, STAT3, FBP1, CLIC1, PAGR1, IGF1R, F2, G6PD, IFNG, COX5A, CRP, KCNJ1, HMGB1, COMT, VCAM1, KIT, ABL1, CALR, ADAM17, DNMT3A, JAK3, FOS, PTPN6, ITGB2, AGT, ACTB, RIPK1, ITGB3, JUN, GAPDH, OPRM1, PIK3CG, HSPA5, FOXP3, SLC12A1, AQP1, SERPINH1, IL6ST, STAT5A, SELP, HMGA2, IL13, PADI4, KCNJ10, WNK4, CCL5, CD47, CD34, RGS2, USF1, LTA, ANK2, EPO, PF4, KLHL3, LDB1, ZFPM1, DMTN, PTGIR, PDE1C, RARB, PRKCA, KCNQ1, PKD2, ALDH1A2, BAZ1B, ABCG1, NRXN2, SLC13A3, VDR, BDKRB2, ABCA1, ABCC9, KAT5, FGFR2, INSR, ADIPOQ, NR2C2, POMC, SIRT1 |


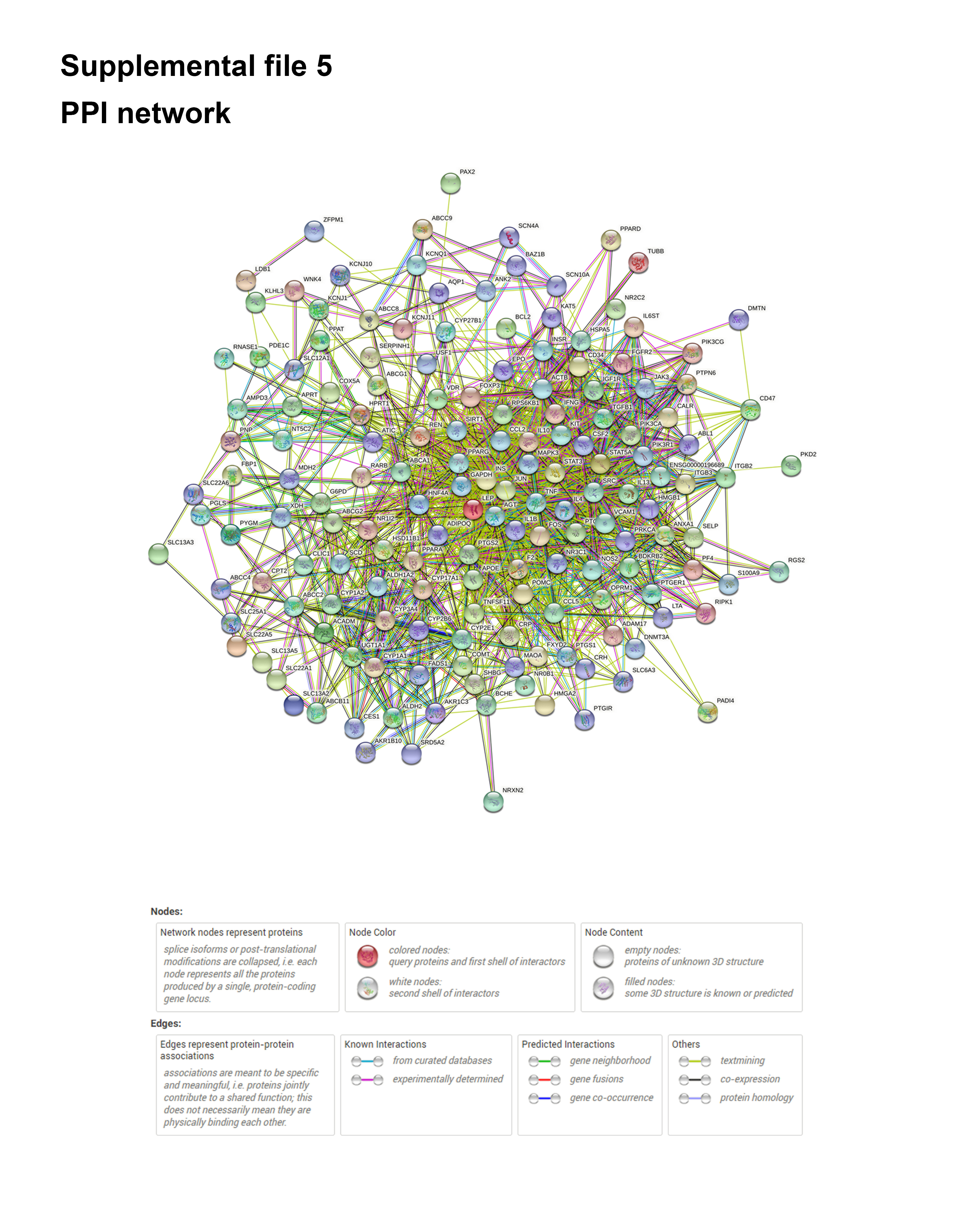


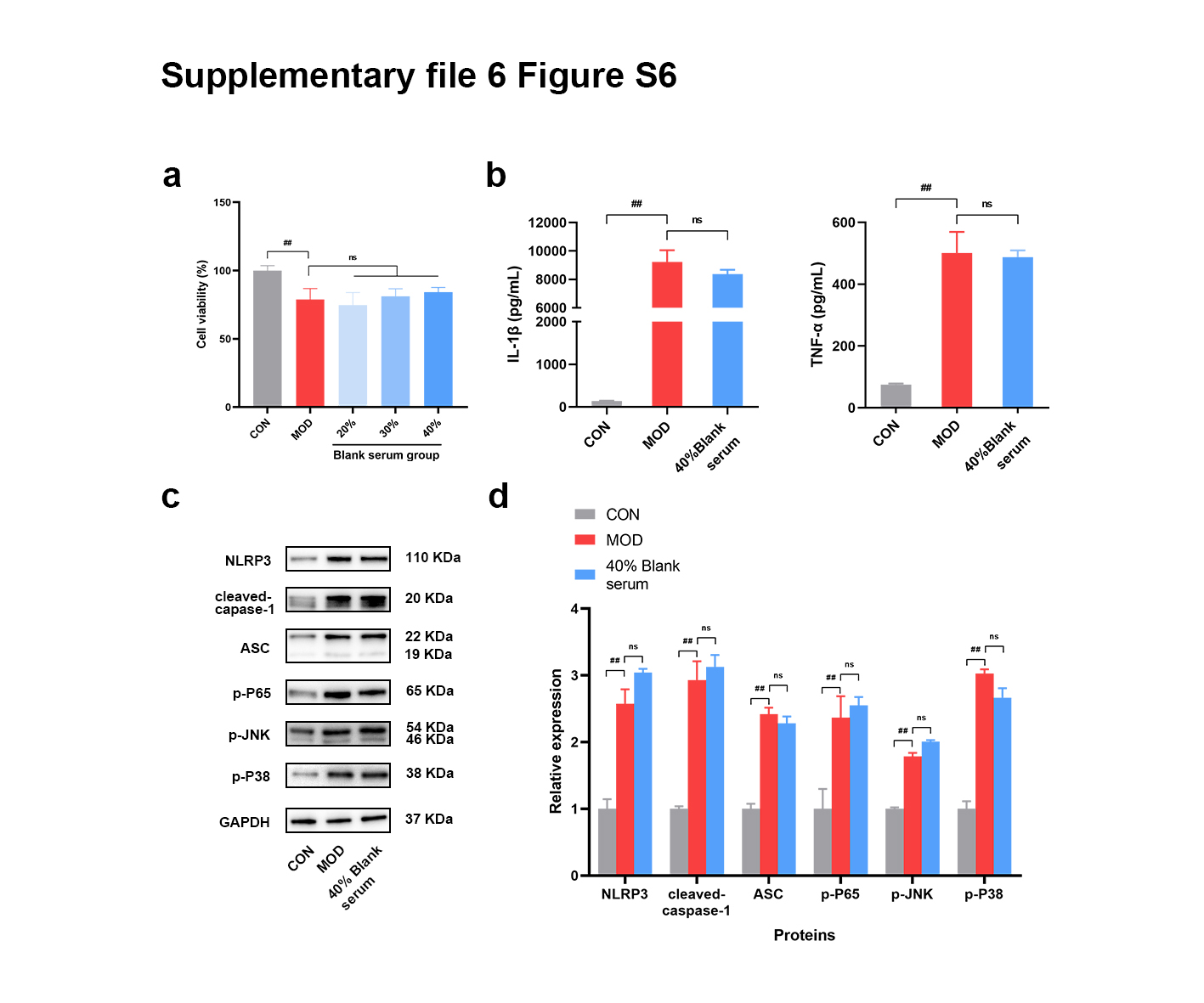


Figure S6. The effect of blank serum. (a) Viability of MSU-activated THP-1 macrophages was evaluated by CCK-8 assay. Values are mean ± SD (n = 6). ^##^*P* ﹤0.01 vs. CON group; ns means *P* ﹥0.05 vs. MOD group. (b) Levels of IL-1β and TNF-α in supernatant of MSU-activated THP-1 macrophages measured by ELISA. Values are mean ± SD (n = 3). ^##^*P* ﹤0.01 vs. CON group; ns means *P* ﹥0.05 vs. MOD group. (c) Levels of NLRP3, cleaved-caspase 1, ASC, p-P65, p-JNK, and p-P38 in MSU-activated THP-1 macrophages were analyzed by western blot (n=3).
